# Time matters! Developmental shift in gene expression between the head and the trunk region of the cichlid fish *Astatotilapia burtoni*

**DOI:** 10.1186/s12864-018-5321-6

**Published:** 2019-01-14

**Authors:** Athimed El Taher, Nicolás Lichilín, Walter Salzburger, Astrid Böhne

**Affiliations:** Zoological Institute, University of Basel, Vesalgasse 1, 4051 Basel, Switzerland

**Keywords:** Cichlid, Gene expression, Developmental shift, Fin development, Adaptation, Phenotypic diversity

## Abstract

**Background:**

Differential gene expression can be translated into differing phenotypic traits. Especially during embryogenesis, specific gene expression networks regulate the development of different body structures. Cichlid fishes, with their impressive phenotypic diversity and propensity to radiate, are an emerging model system in the genomics era. Here we set out to investigate gene expression throughout development in the well-studied cichlid fish *Astatotilapia burtoni*, native to Lake Tanganyika and its affluent rivers.

**Results:**

Combining RNA-sequencing from different developmental time points as well as integrating adult gene expression data, we constructed a new genome annotation for *A. burtoni* comprising 103,253 transcripts (stemming from 52,584 genomic loci) as well as a new reference transcriptome set. We compared our transcriptome to the available reference genome, redefining transcripts and adding new annotations. We show that about half of these transcripts have coding potential. We also characterize transcripts that are not present in the genome assembly. Next, using our newly constructed comprehensive reference transcriptome, we characterized differential gene expression through time and showed that gene expression is shifted between different body parts. We constructed a gene expression network that identified connected genes responsible for particular phenotypes and made use of it to focus on genes under potential positive selection in *A. burtoni*, which were implicated in fin development and vision.

**Conclusions:**

We provide new genomic resources for the cichlid fish *Astatotilapia burtoni*, which will contribute to its further establishment as a model system. Tracing gene expression through time, we identified gene networks underlying particular functions, which will help to understand the genetic basis of phenotypic diversity in cichlids.

**Electronic supplementary material:**

The online version of this article (10.1186/s12864-018-5321-6) contains supplementary material, which is available to authorized users.

## Background

Variation in the expression of an invariant genome to produce diverse cell types during embryogenesis is crucial for animal development [1]. During that time, the spatial and temporal coordination of gene expression is necessary for cell specification and cell differentiation [2]. In the last decades, many studies have probed the relationship between the spatiotemporal regulation of gene expression and cell differentiation and fate [3–5]. Although numerous aspects of development have been discovered through the study of model organisms, in-depth analyses of full transcriptome gene expression profiles for different developmental stages of non-model organisms are largely lacking [6]. This hinders a comparative view on the evolution of developmental gene expression patterns across the animal kingdom.

In this study, we present a novel approach to constructing a comprehensive transcriptome from RNA-sequencing (RNA-seq) of specific developmental stages post hatching and provide a first insight into spatiotemporal gene expression changes for an emerging fish model system, the cichlid *Astatotilapia burtoni* [7]. This fish inhabits East African Lake Tanganyika and its affluent rivers [8] and belongs to the most species-rich lineage of East African cichlids, the haplochromines. Over the past decades, *A. burtoni* has been established as a model system to study behaviour [9], neuronal processes [10, 11], sex determination [12–15], pigmentation [16] as well as genomics and speciation [8, 17, 18]. As a consequence, *A. burtoni* is also an emerging system in developmental biology [19, 20], which is greatly facilitated by the availability of a reference genome [17]. This genome, however, remains fragmented (scaffold level assembly) and with poorer annotations as compared to the most widely used cichlid reference genome, the one of the Nile tilapia *Oreochromis niloticus*, which has been assembled at the chromosome level [17, 21].

To provide an expression catalogue of *A. burtoni* development, we sequenced in-depth the transcriptome of *A. burtoni* embryos and larvae at three important developmental time points: 8 days post fertilization (dpf), 14 dpf and 20 dpf (for fish images of these stages see Additional file 1: Figure S1). At 8 dpf the embryos are just hatched but already start to swim actively [20], and have roughly developed the rudiments of all organs [22]. At 14 dpf, the larvae have attained their adult body plan, all structures of the adult body are present [20] (“direct” mode of development with no prolonged larval period) and sex is likely already determined [12]. It is also around that time that juveniles are released from their mother’s mouth and start feeding on their own [20]. At 20 dpf the early juvenile fishes have finished their embryonic development and become sexually dimorphic [12].

Combining these new developmental RNA-seq data with further available transcriptome information for *A. burtoni*, we first aimed to construct the most comprehensive reference transcriptome possible, to generate a new resource for this emerging fish model system. Next, we compared our transcriptomic data to the reference genome as well as other cichlid genomes to identify transcripts lacking from the genome assembly, thereby functionally annotating the transcriptome. Using our developmental data, we then profiled expression changes at important developmental time points and constructed a gene expression network. This network will serve as yet another resource and constitutes the basis for studies of gene-gene interactions at the expression level towards revelation of functional relationships.

## Results

### An improved reference transcriptome for *Astatotilapia burtoni*

The developmental tissue samples were derived from a previous study [12] that comprised male-only samples from 7 until 48 dpf focusing on the development after hatching. In this experiment, head and trunk of the hatchlings were split to separate brain and gonad into their proxies, head and trunk, following the standard methods in the field (e.g. [23–25]). Starting at 8 dpf, sufficient RNA could be extracted from each body part, which here allowed us to perform in-depth RNA-seq of single samples instead of pools (resulting in a total of 18 libraries, Additional file 2: Table S1, stranded RNA-protocol Illumina Next-Seq PE 75). However, a dissection of single organs for individual RNA-libraries is not possible at these stages.

In order to generate a comprehensive expression catalogue, we used all our developmental samples (9 individuals) to identify expressed regions not yet annotated as such in the existing *A. burtoni* genome assembly (RefSeq assembly version GCF_000239415.1 AstBur1.0, [17]).

**Fig. 1:**
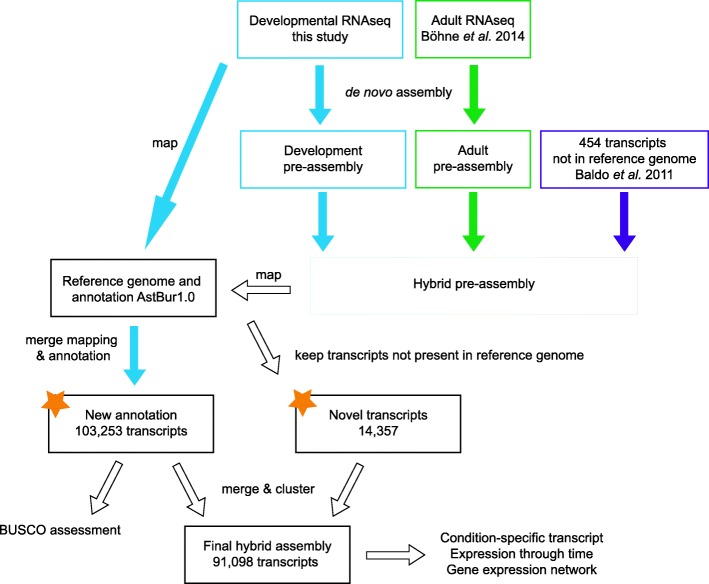
Comprehensive transcriptome construction for *A. burtoni*. A new transcriptome resource was generated with two approaches; the available genome annotation for *A. burtoni* was redefined by mapping RNA-seq data from *A. burtoni* developmental stages to the reference genome (resource 1, new annotation; Additional file 3: Data S1). In a second step we generated de novo assemblies from developmental and adult [26] data and combined them with a previously published transcriptome dataset [27]. From this hybrid pre-assembly, we kept transcripts that were missing from the genome reference (resource 2, novel transcripts; Additional file 4: Data S2). Novel transcripts were combined with transcripts from the novel annotation. To study transcript expression through time, this combined dataset was clustered to collapse splicing variants to a minimum and resulted in a final hybrid assembly of 91,098 transcripts. For further workflow details see also Additional file 1: Figures S2 and S4. Orange stars: resources generated in this study provided as Additional files 3 and 4: Data S1 and S2

We used two different approaches to assign RNA-seq reads to transcripts (for an overview of the workflow see Fig. 1). We first mapped our new RNA-seq reads onto the available *A. burtoni* reference genome to identify transcripts and potentially gene loci not yet present in the *A. burtoni* genome annotation (RefSeq assembly version GCF_000239415.1 [17], Additional file 1: Figure S2). This resulted in an annotation for a total of 103,253 transcripts, which contained 4560 out of 4584 core genes conserved across actinopterygii (Additional file 2: Table S2 and Additional file 3: Data S1). With these transcripts we could confirm all previously annotated gene loci (26,776 loci with 48,667 annotated transcripts) present in the current genome annotation. We further added expression data for 20,903 new loci containing 51,637 novel exons (Additional file 1: Figure S3, Additional file 2: Table S2). Interestingly, a substantial portion of these (14,726 transcripts) were located in regions previously defined as intergenic (Additional file 1: Figure S3).

In a second approach, we de novo assembled all our developmental RNA reads as well as already published *A. burtoni* RNA-seq data from adult individuals [26, 27] (Fig. 1 and Additional file 1: Figure S4). We compared these transcripts to the reference genome to identify transcripts not yet assembled in the current reference genome. This resulted in an additional 14,357 potentially novel transcripts not present in the current genome release (Additional file 2: Tables S3 and S4 and Additional file 4: Data S2), which can be grouped into 13,116 genes according to the Trinity gene classification.

**Fig. 2:**
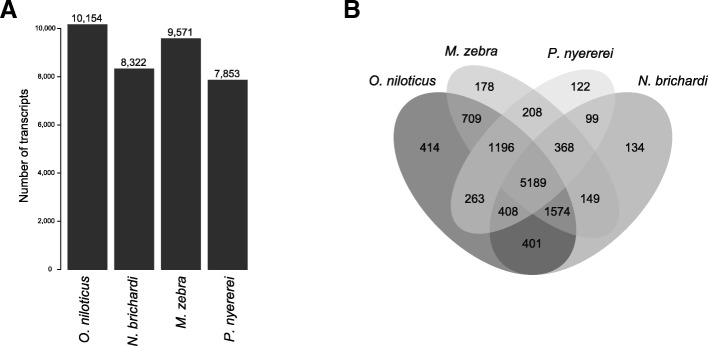
De novo assembled transcripts missing in the *A. burtoni* reference genome but present in other cichlid genomes. **a** Number of transcripts that could be identified in the other available cichlid genomes. **b** Intersection of the transcripts found in other available cichlid genomes but absent from the *A. burtoni* reference genome

**Fig. 3:**
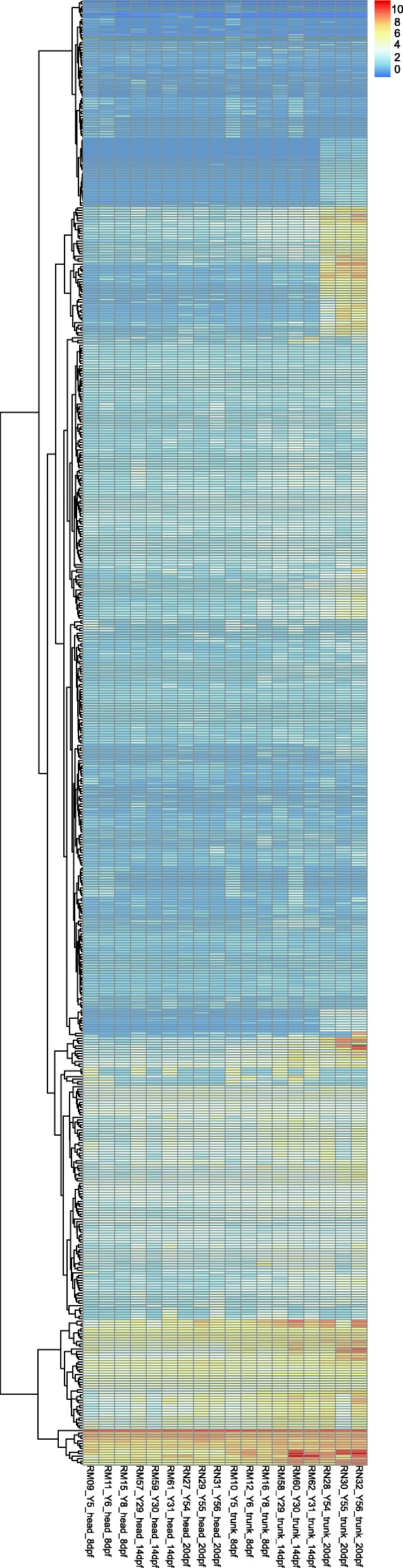
Expression profiles of novel transcripts in *A. burtoni*. Heatmap of the *rlog* normalized count for the 2944 identified novel transcripts that are neither present in the *A. burtoni* reference genome nor in any of the four available cichlid genomes

To proceed with functional annotation and comparative expression analyses across development, we combined these novel transcripts with the transcripts resulting from the reference-mapping and obtained, after a final clustering approach, a “hybrid” assembly containing 91,098 transcripts (composed of 34% transcripts identical to the previous reference, 50% newly defined transcripts present in the genome, 16% transcripts missing from the genome release). Next, we assigned putative functions to the transcriptome by comparing it to available sequence data (all GO annotations per transcript are provided in Additional file 5: Data S3). We could retrieve sequence identities indicative of homology to known nucleotide sequences for 73% of the transcripts in the hybrid transcriptome assembly (96% of the transcripts present in the reference transcriptome; 69% of the newly annotated transcripts; and for 35% of the novel transcripts). For 94% of the transcripts with an annotation, this annotation stemmed from sequence similarities to actinopterygian sequences (summary for taxon annotation in Additional file 2: Table S5, for detailed functional annotation see Additional file 5: Data S3). In addition to this annotation, we identified open reading frames (ORFs) in 92% of the transcripts stemming from the reference transcriptome, in 50% of the newly annotated transcripts and in 10% of the novel transcripts. To further characterize these novel transcripts, we aligned them to all available cichlid genomes [17]. We found that 79% of these transcripts were present in at least one other cichlid genome and 36% of them were found in all four cichlid genomes strongly suggesting that the novel transcripts are indeed lacking from the current *A. burtoni* genome assembly due to assembly quality (Fig. 2). We further investigated the transcripts with no hit to any cichlid genome (2944 of the novel transcripts) for their expression and function. We found that, compared to the rest of the transcriptome, these transcripts were enriched in particular gene ontology (GO) categories (Additional file 1: Figure S5), many of which are regulatory such as “positive regulation of telomerase activity” or “negative regulation of transcription by polymerase I” The expression profile of those 2944 transcripts in all 18 developmental samples is represented with a heatmap in Fig. 3 and shows several highly expressed transcript clusters.

### Spatiotemporal gene expression variation

**Fig. 4:**
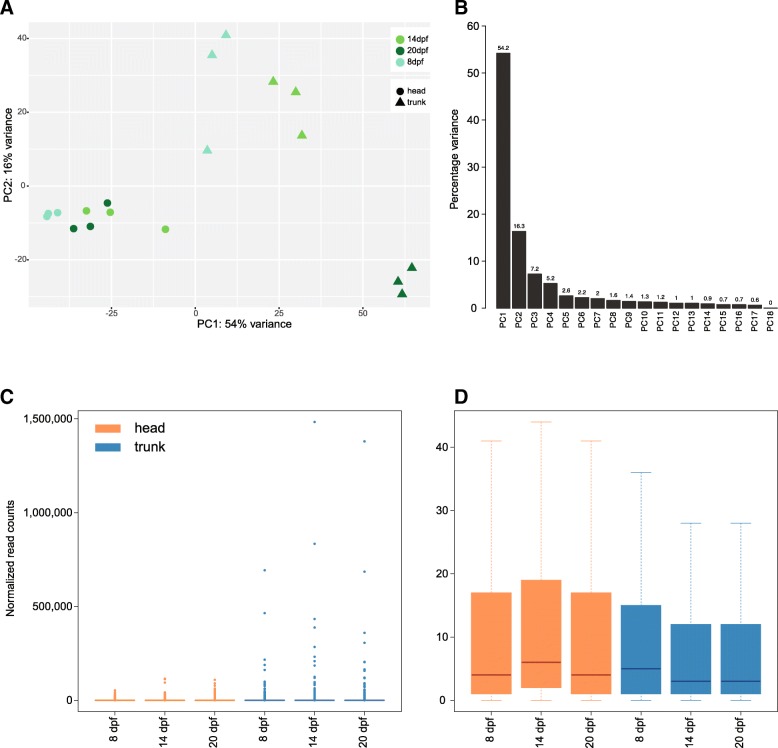
Gene expression variation throughout time and between body parts**. a** Main variation of transcript expression between the two embryonic body parts (head and trunk) at three different developmental stages (8 dpf, 14 dpf and 20 dpf) illustrated by a PCA. The axes represent the percentage of variance explained by PC1 (x-axis) and PC2 (y-axis). **b** Principal components of the transcript expression PCA shown in panel (**a**). **c** Distribution of median transcript expression within each sample type. **d** Distribution of median transcript expression within each sample type after outlier removal. Orange: head, blue: trunk

To obtain an overview of gene expression variation throughout development and between head and trunk, we performed a principal-component analysis (PCA). The variance was separated along axes correlated with body parts but also substantially with developmental time points. There was more separation between the different time points in the trunk than in the head. Especially the trunk samples at 20 dpf were clearly different from the samples at 8 and 14 dpf. In addition, in the trunk the variance in gene expression among replicates was lower for the late developmental stages (20 dpf replicates clustered closer together in the PCA than samples at 8 or 14 dpf). The opposite trend was found for the head (strongest overlap of replicates at 8 dpf). When excluding extremely highly expressed transcripts, the variance of expression was actually a bit higher within the head samples (Fig. 4d), suggesting that these extreme gene expression outliers in the trunk likely drove the initial pattern. The most extremely expressed transcripts were annotated as housekeeping genes with a muscular function such as *myosin heavy chain* (Fig. 4c, Additional file 2: Table S6). A GO analysis of tissue-specific outliers (Fig. 4c) revealed global functional differences between the two body parts; the head-specific outliers showed enrichment for head related functions (e.g. “glutamate secretion”, “phototransduction”, “*Wnt* signalling pathway”, Additional file 1: Figure S6A) and the trunk group in trunk related functions (e.g. “fin regeneration”, “cardiac muscle contraction”, “muscle attachment”; Additional file 1: Figure S6B).

### Differences in gene expression between head and trunk throughout developmental time

**Fig. 5:**
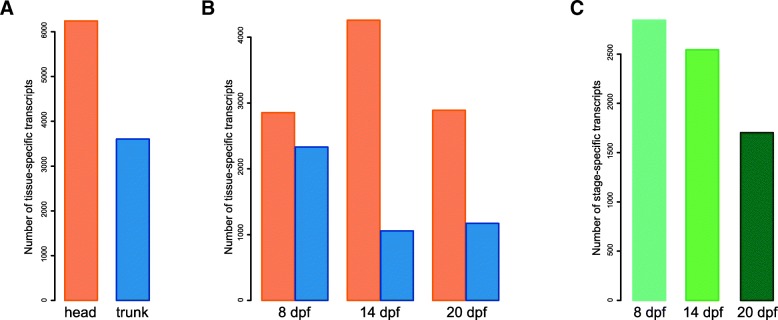
Specificity of transcript expression in head and trunk and across developmental time**. a** Number of tissue-specific transcripts expressed in the head (orange) and in the trunk (blue). **b** Number of tissue-specific transcripts expressed in each tissue at each time point. **c** Number of stage-specific transcripts for both, the head and trunk samples combined

We identified 6246 transcripts exclusively expressed in the head and 3603 transcripts exclusively expressed in the trunk (Fig. 5a): The head expressed a maximum number of tissue-specific transcripts at 14 dpf, while the trunk expressed a maximum number of tissue-specific transcripts at 8 dpf (Fig. 5b). Grouping together both body parts, we detected a decrease of stage-specific transcripts throughout developmental time: 2844 transcripts were only expressed at 8 dpf, 2545 transcripts only at 14 dpf and 1703 transcripts at 20 dpf. The number of stage-specific transcripts was similar between 8 dpf and 14 dpf while being much smaller at 20 dpf (Fig. 5c). A GO analysis for the tissue-specific genes found – as expected – an enrichment in head-related functions (e.g. “pyramidal neuron differentiation”, “Schwann cell development”, “cone photoresponse recovery”, “phototransduction”, Additional file 1: Figure S7A) in the head, while the trunk showed enrichment in trunk-related functions (e.g. “swimming”, “swim bladder morphogenesis”, “heart rudiment development”; Additional file 1: Figure S7B). GO enrichment analysis for stage-specific genes showed that early stages were enriched in more functions and in more general developmental processes while the latest stage was enriched in only 12 GO categories with five of them involved in heart development (Additional file 1: Figure S7C-E).

**Fig. 6:**
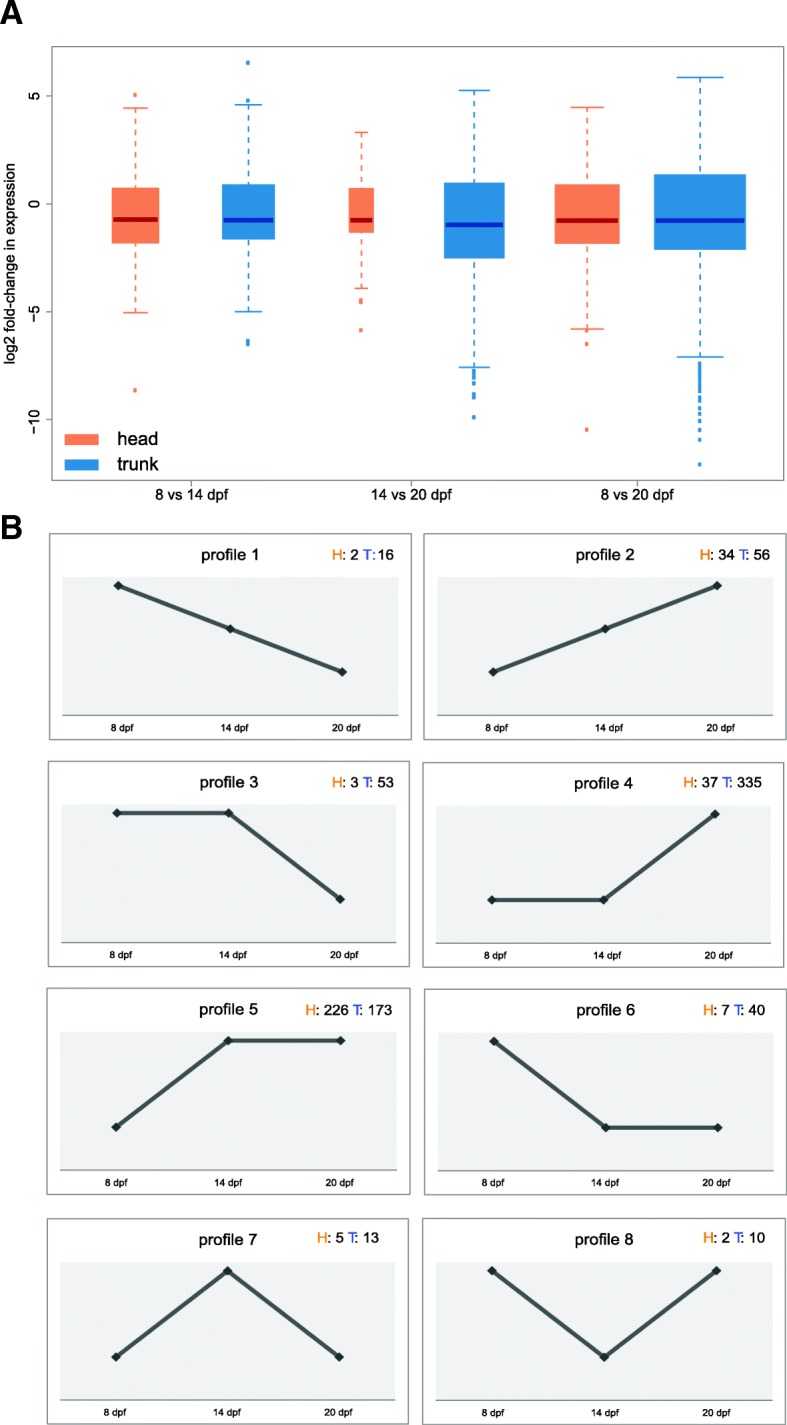
Differential transcript expression through developmental time**. a** Variance of transcript expression changes for all pairwise comparisons in the head (orange) and in the trunk (blue). **b** Detected transcript expression changes across time can be represented with eight different expression profiles. Transcripts were grouped into these main expression patterns by intersecting the list of transcripts differentially expressed between the different time points. The number of transcripts in each profile for head (H) and trunk (T) are indicated

To investigate gene expression changes throughout time, we next assessed patterns of differential transcript expression within each body part across the three time points (Additional file 2: Table S7). The magnitude of the variation difference between the head and the trunk can be illustrated with the log2 fold-changes (Fig. 6a). In addition to more transcripts that are differentially expressed through time in the trunk, the magnitude of variation of expression changes was larger in the trunk than in the head. This pattern was already evident from the PCA (Fig. 4a).

These expression changes can be visualized with eight expression profiles (Fig. 6b). The number of genes belonging to each profile varied between the two body parts (Additional file 2: Table S8); in general, we observed more up-regulation of expression than down-regulation (fewer transcripts assigned to profiles 1, 3 and 6 than to profiles 2, 4 and 5). Again, profile 4 was the most common in the trunk transcripts (up-regulation at 20 days compared to 8 and 14), followed by profile 5 (the most common in the head), which grouped transcripts up-regulated at 14 and 20 dpf compared to 8 dpf. GO analyses for the gene sets assigned to the eight different profiles (for each body part) revealed functional differences between the sets (Additional file 1: Figures S8-S15). The profiles that matched a down-regulation of expression throughout developmental time were enriched in more general functions (e.g. “DNA recombination” in profile 1, Additional file 1: Figure S8B), whereas the profiles showing an increase in gene expression over time were enriched in more specific developmental processes (e.g. “detection of external stimulus” and “detection of light stimulus” for profile 2, Additional file 1: Figure S9A).

### Transcriptional network in *A. burtoni* developmental stages

**Fig. 7:**
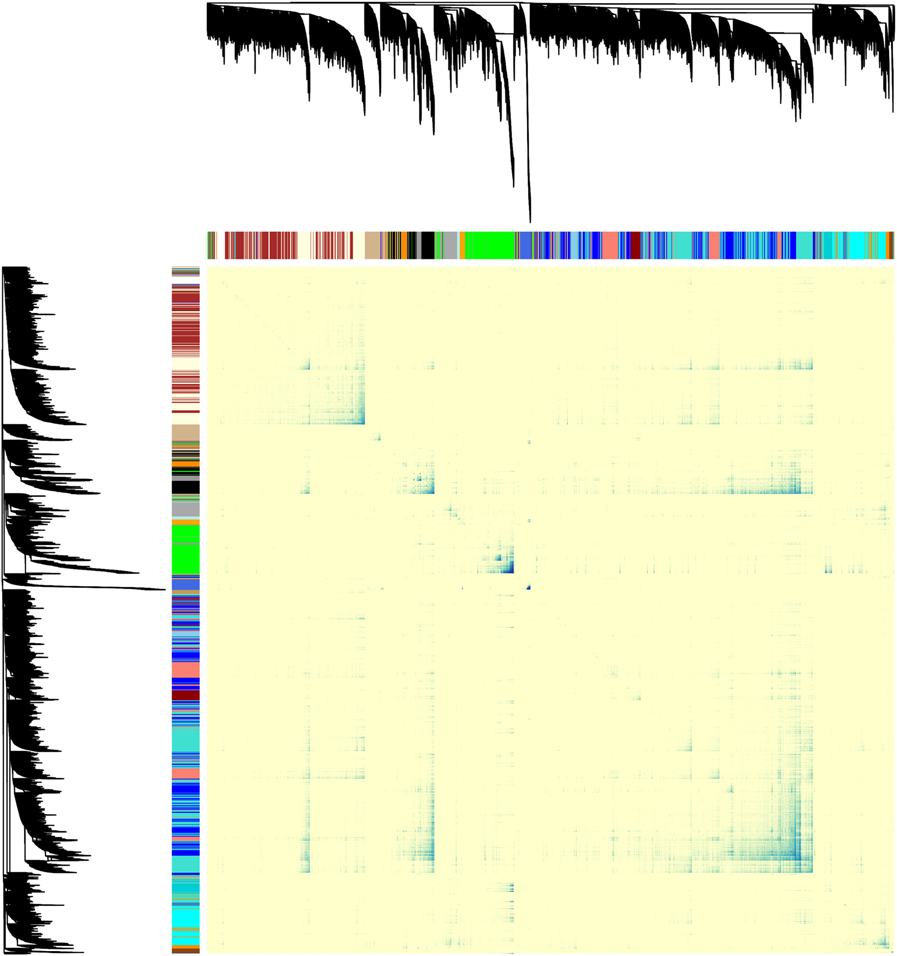
Network heatmap plot. After applying a re-clustering approach, a stable network of 21 modules was constructed including 28,560 transcripts. The topological overlap values in the matrix are colored light blue for low overlap and progressively darker blue to higher overlaps. The transcript module assignment dendrogram is shown along both sites of the heatmap

Using an iterative reclustering approach we constructed a stable network consisting of 21 modules, which retained 28,560 transcripts (of the initial 91,098 transcripts), representing 23,857 gene loci. The module sizes varied from 148 to 5283 transcripts (Additional file 2: Table S9, complete list of transcripts and their module association Additional file 5: Data S3). The scale-free topology model fit stabilized at an R^2^ = 0.88 and power of 16 (Additional file 1: Figure S16). The network heatmap showed a particularly strong topological overlap for genes within the royal blue, green and black module (dark blue colour Fig. 7).

Next, we checked for positive correlations between modules and traits to reveal trait-specific functions within modules (Additional file 1: Figure S17). We indeed detected three modules that were in general correlated with a particular time point: royal blue with 14 dpf, 14 dpf head and 14 dpf trunk; cyan with 8 dpf, 8 dpf head and 8 dpf trunk and orange with 20 dpf, 20 dpf head and 20 dpf trunk.

The royal blue module was highly enriched for biological processes related to brain features such as “amygdala development”, “layer formation in cerebral cortex” “regulation of timing of neuron differentiation” and “aggressive behavior”. In addition, “swimming” was also an enriched function in this module. The cyan module was enriched for transferase and kinase functions (Additional file 1: Figure S18C). The orange module was also enriched for kinase functions and membrane transport as well as DNA integration. Other modules seemed to be strongly correlated with a certain body part. For example, the blue module correlated with head samples in general, 14 dpf head and 20 dpf head. Matching this pattern, this module showed a functional enrichment for vision components (Additional file 1: Figure S18). The black module was highly correlated to trunk samples in general and trunk 14 dpf and had an enriched annotation for muscular and swimming functions. The turquoise module was highly correlated to head in general and also to head 8 dpf; module dark orange was correlated to trunk 8 dpf; finally module green was correlated to trunk 20 dpf and had oxygen-blood functions.

All modules had an overrepresentation for the GOs belonging to the categories “biological process” and “molecular function” (Additional file 1: Figures S19 and S20), while not all modules had an enrichment for GOs belonging to category “cellular component” (i.e., dark red, orange, salmon and white modules). “Metabolism”, “development”, “cell organization”, “biogenesis” and “biosynthesis” were the most important GO categories within biological processes that appeared within over (brown, light yellow) as well as underrepresented (green, dark red, blue) GO terms. Within the GO class “molecular function”, the most abundant terms within over as well as underrepresented GOs were “catalytic activity”, “binding” and “transferase activity”.

### *A. burtoni* novel transcripts in gene expression network modules

From the 2944 novel transcripts, 451 were successfully integrated into the network within mainly the modules green (190 transcripts), black (79 transcripts), saddle brown (50 transcripts), and dark grey (26 transcripts) (Additional file 2: Table S10). The green module showed a GO enrichment for blood related functions (e.g., “oxygen transport”) as well as “protein-binding” (Additional file 1: Figure S18E). The black module was related to muscle function (Additional file 1: Figure S18A). The saddle brown module seemed to be related to extracellular and cellular components (Additional file 1: Figure S21A), and the dark grey module had a wide diversity of functions such as “aerobic respiration” and “extracellular matrix structural constituent” (Additional file 1: Figure S21B).

### Gene expression of genes evolving under positive selection

In order to identify new potentially selectively advantageous genetic variants, we investigated our transcriptome for transcripts evolving under positive selection. Compared to the Nile tilapia and the zebrafish as an outgroup (Additional file 1: Figure S22), we identified 31 transcripts under positive selection in the branch leading to *A. burtoni*, 28 of which originated from the reference transcriptome and three from the newly annotated transcripts (Additional file 2: Table S11). Among the transcripts under positive selection, we identified one gene (*A. burtoni* GeneID 102309059 *red-sensitive opsin-like,* new reference rna3827) belonging to the opsin gene family. Opsins are key to visual adaptions in new habitats [28]. The opsin gene was only expressed in the head (Additional file 1: Figure S23), concordant with its function in vision, and its expression cannot be detected before 14 dpf. We investigated GOs of all transcripts under positive selection, and detected enrichment in 21 GO categories. Interestingly, three of them (“fin morphogenesis”, “pectoral fin development”, “fin regeneration”) are implicated in fin development, two in glutamate signalling, and several other metabolic categories, whereas the others are rather broad functional categories such as “DNA repair” (the most abundant GO category) (Additional file 1: Figure S24).

### Tracing candidate genes in the expression catalogue and network

In order to demonstrate the applicability of our generated resources, we decided to perform a case study based on four different genes: *fhl2a*, *fhl2b*, *col1a1* and the opsin gene. We chose to investigate them in more detail due to their potential functions in swimming, vision and pigmentation. Among those four genes, two were identified as being positively selected in our dataset (*col1a1* rna38941 and the opsin gene rna3827). Opsins are key to visual adaptions in new habitats [28] and *col1a1* was annotated for all the GO categories with a function in fin development and also retained in the gene expression network. *Fhl2a* (MSTRG.32833.1) and *fhl2b* (rna9150) have been implicated with the ontogenetic development of a key colour innovation in haplochromine cichlids, the anal fin egg-spots [16]. Within our new reference transcriptome, the *fhl2a* gene boundaries have been redefined, whereas the three other genes remained identical to the existing reference. The opsin gene and *fhl2a* were specifically expressed in the head in our set-up, *fhl2b* was expressed at very low levels in both body parts (below our threshold applied in differential expression analysis), and *co1la1* was highly expressed in both body parts. None of the genes was stage-specific; The opsin gene and *col1a1* were both differentially expressed; The opsin gene was up-regulated in head at 20 dpf, while *col1a1* was up-regulated in the trunk at 14 dpf congruent with their respective functions in vision and fin development (see Additional file 1: Figure S23 for expression profiles).

Within the expression network, *fhl2b* and the opsin gene were assigned into the large blue module (4032 transcripts), which is functionally enriched for the GO terms related to vision (“visual perception”, “structural constituent of eye lense” and “cone photoresponse recovery”, Additional file 1: Figure S18B). However, *fhl2a* was clustered into the white module (with 2476 other transcripts) that showed functional enrichment in transcription and immune system GO categories (Additional file 1: Figure S25A). *Col1a1* was clustered in the light yellow module (2884 transcripts), which showed enrichment for “fin regeneration” among other functions (Additional file 1: Figure S25B). Focusing on this GO class, we identified nine transcripts with GO annotations for “fin development”, four for “fin morphogenesis”, five for “pectoral fin development” and 13 for “fin regeneration”, with a total of 20 unique transcripts associated to “fin development” (Additional file 2: Table S12). *Col1a1* (transcript ID rna38941) is the only transcript annotated for all fin-related GOs.

An intramodular analysis (Additional file 1: Figure S26) for modules containing the four genes (i.e., white, blue, and light yellow) revealed a significant correlation between each of the four genes within a trait (i.e., gene significance GS) and the module membership (i.e., how correlated a gene is to a module). The four selected genes were found to occupy a relatively central position, highlighting them as important features for the given trait within the selected module.

## Discussion

### *A. burtoni* genome annotation improvement

The annotated number of genes in the *A. burtoni* reference genome (26,776 gene loci including 24,094 protein-coding genes) is certainly an underestimation when comparing it to the closest related species with a chromosome level genome assembly (the Nile tilapia *Oreochromis niloticus*, annotated gene loci 42,622 annotation release 104). Through our extensive set of novel transcript expression data, we provided support for the expression of all these loci during development and evidence for the expression of an additional 25,808 loci, which are likely missing from the annotation since the original annotation process did not include extensive developmental data. ORF prediction indicated that about 50% of the newly defined transcripts have coding potential. This suggests that especially the annotated non-coding part of the genome is incomplete. Improving the current genome annotation allows a more accurate quantification of gene expression when using the reference genome by attributing more sequencing information to gene features. We provide access to this resource to the research community in form of an annotation file in standard gtf file format.

In addition to extending the available genome resource, we applied a reference free approach, which resulted in the construction of 14,356 transcripts that are not only missing from the annotation but also from the *A. burtoni* genome assembly. About 10% of those contained ORFs. One explanation why these transcripts are not present in the reference genome assembly might be because we used a different source population for our experiments compared to the reference genome. The *A. burtoni* reference genome was derived from a laboratory stock, which goes back to a natural population of the Northern part of Lake Tanganyika [29]. Although also belonging to the northern/southwestern haplotype clade described by Pauquet et al. [8], our laboratory stock population belongs to a different mitochondrial haplotype (haplotype 2) than the one of the laboratory source stock of the reference genome (individuals of the same stock belong to haplotypes 9 and 3 [8]) and has originally been collected at a different site [29]. However, since most of the newly assembled transcripts were found in at least one other cichlid genome, a second explanation, which is that these transcripts are missing from the *A. burtoni* reference genome due to assembly or sequencing artefacts, seems more likely to us. Still, the reference genome is from a female specimen while our *A. burtoni* embryos are derived from a male-only developmental series [12]. We have previously shown that our laboratory stock has an XY sex-determining system on LG5 [12, 13] so that assembled transcripts not found in the reference genome may well be Y-specific.

The rather low number of ORFs (~ 50% for the transcripts defined by StringTie and 10% for the transcripts missing form the genome assembly) found in the novel transcripts suggests that the majority of them are non-coding and thus might be regulatory. It has previously been shown that many genomic novelties in cichlids are based on UTRs and non-coding RNAs, which are potentially involved in regulatory changes of expression profiles [17, 27].

When inspecting the 2944 newly assembled transcripts specific to our *A. burtoni* developmental dataset (i.e. transcripts not present in any other cichlid reference genome), we indeed found a functional enrichment in transcriptional processes. This result is not surprising as it has become increasingly evident during the past few years that gene regulation plays an important role in adaptation and speciation [18, 30]. Gene expression levels play a key part in the diversification of phenotypes [30–32]. *A. burtoni* transcriptional novelties might be modulators of gene expression and hence interact with phenotypic diversity. From those genomic novelties, 451 transcripts were present in the network construction. The modules including those transcripts, especially the green and black modules, showed GO enrichment for physiological processes, such as blood and muscle functions, that might contribute to species divergence [33].

### Developmental time shift for the head and the trunk

We identified 6246 transcripts exclusively expressed in the head and 3603 transcripts exclusively expressed in the trunk during the first 20 days of development post fertilization. When looking at all data, the number of differentially expressed transcripts between the different stages and the overall expression variation over time was higher in the trunk. These differences suggest that the timing of development for the two body parts is shifted. On the one hand, this could indicate that the tissues originating in the trunk are not yet as developed as the ones originating in the head and therefore more expression differences between the different stages are observed in the trunk compared to the head. On the other hand, the number and the diversity of organs originating in the trunk (heart, spleen, liver, stomach, intestine, gonads, kidney, swim bladder, urinary bladder, pancreas, spinal cord) is higher compared to the head (brain, eyes, gills, nose, ears) [34]. This could mean that, overall, more genes have to be differentially regulated over time leading to an overall higher expression variation across time in the trunk. It has previously been shown that tissues do not develop at the same time during embryogenesis [34]. In human embryogenesis, for example, the organs near the main neural area (typically the head) develop earlier than areas of the body that will be in the posterior part of the body (cephalocaudal development). In addition, the hypothalamus originates in the head. This structure is the command centre of the endocrine system and secretes various hormones that directly provoke responses in target tissues and therefore, the development of the head may be necessary to trigger the development of trunk specific tissues. That there are development shifts between body parts has already been demonstrated in the Nile tilapia where sexually dimorphic aromatase activity can be detected in the brain even before any ovarian differentiation [35]. The strong separation of gene expression in the trunk between 20 dpf and 8/14 dpf in the PCA could reflect the fact that at stage 20 dpf the fish have finished embryogenesis and the majority of organs have finished developing. Given that the trunk contains more varying organs than the head, this could also explain why the pattern is so pronounced in the trunk but not in the head.

When focusing on genes expressed only at one specific time point, regardless of the tissue of expression, we found that there are functional differences between the stages: More stage-specific genes with more general functions are expressed during early embryogenesis compared to later stages probably indicating overall high levels of transcription and metabolism during earlier stages where more tissues are still developing. It could also reflect the necessity for the early stages to express many genes in charge of triggering important developmental pathways needed only for a short period of time while the later stages are in charge of expressing genes with more general functions and needed constantly.

In order to provide a further functional categorization and transcriptomic resource for *A. burtoni*, we constructed a gene expression network consisting of 21 modules, in which especially the smaller modules could be attributed to particular functions. The usefulness of our transcriptome data and the network was illustrated by our focus on four candidate genes.

We focused on the placement of two particular genes that we first showed to evolve under positive selection, an opsin gene and a fin development gene (*col1a1*). In agreement with their function, these two genes showed over-expression in the head and the trunk, respectively. The co-expression module that contained the opsin gene was enriched for functions involved in vision and highly correlated to head tissue (blue module), while the light yellow module that contained the fin development gene was correlated with trunk tissue and enriched for “fin regeneration” and muscle functions. The light yellow module grouped transcripts together that were annotated with several other GO terms related to fin development and functioning. This illustrates that the gene expression network indeed recapitulates functional relatedness on the expression level. The network modules can serve as a starting point for in-depth studies of particular functions such as the development of vision or body plan establishment and maintenance or to investigate the role of genes that are accumulating potentially advantageous mutations and thus be adaptive.

We also investigated the expression and placement of two genes that have been implicated with a novel pigmentation pattern in haplochromine cichlids, *fhl2a* and *b*. Whereas sequence changes in the b-copy are probably linked to the emergence of the egg-spot pigmentation trait, the a-copy was suggested to play a more downstream function in the establishment of the pigmentation pattern [16]. Our expression and network data suggested that the b-copy might also be connected to vision since it is over-expressed in head tissue and placed in the same module than the opsin gene (blue module), which showed functional enrichment for vision. The different expression patterns and placement in different gene expression modules of these duplicated genes suggested that they have distinct functions in development.

## Conclusion

With an integration of our new sequencing data from different developmental time points we could improve the genomic resources for the cichlid fish *Astatotilapia burtoni* and provide a multitude of novel transcripts for this fish. We showed that transcriptome sequencing can reveal novel transcripts with putative regulatory functions. Focusing on gene expression through time, we established gene expression modules, which help to reveal functions of novel transcripts in important physiological processes.

*A burtoni* is a member of the most species-rich lineage of cichlids and hence our approach and data will be beneficial to a large community. Expression data from controlled developmental time points are largely missing from current transcriptome projects, which could result in an underestimation of expression divergence and dynamics in cichlids.

Besides serving as a new resource for the scientific community, the *A. burtoni* transcriptome will allow us to focus on key steps in the development and study the interactions of genes at the expression level. Our transcript catalogue revealed a substantial number of novel genes, with a potential function in transcription. This first developmental transcriptome sets the basis to study the evolutionary origin of new genes as well as their function across, cichlids, one of the most species-rich families among vertebrates.

## Methods

### Samples and RNA-sequencing

The RNA samples were taken from a male-only developmental series of *A. burtoni*, (laboratory strain, Zoological Institute, University of Basel, Switzerland; all experiments involving animals were performed in accordance with public regulations under the permits no. 2317 and no. 2620 issued by the cantonal veterinary office of the canton Basel Stadt, Switzerland), previously generated to study sexual development [12]. In this previous study, eggs derived from fertilization of a YY-supermale were collected within an hour after fertilization and incubated in the same fish facility as the adult fish in an Erlenmeyer at 24 °C with constant airflow in a 12 h dark–light cycle [12]. They were subsequently transferred to aquaria. Samples were taken exactly 8, 14 and 20 days after fertilization. Total RNA was extracted separately from head and trunk of three male *A. burtoni* embryos, for the three different time points. Individual sequencing libraries were constructed for each of the 18 samples at the D-BSSE (Department of Biosystem Science and Engineering, ETH Zurich) after ribo-depeletion using the Illumina TruSeq stranded-protocol. Libraries were pooled and sequenced on an Illumina NextSeq in PE 75 bp mode.

### De novo assemblies

We ran a de novo assembly of all the reads of the 18 individual libraries together as well as an adult assembly on brain and gonad samples of a previously published dataset of *A. burtoni* (females and dominant males [26], 12 libraries, Additional file 1: Figure S4). Illumina sequences of all 30 libraries were filtered and adaptors were trimmed with Trimmomatic version 0.33 [36] (Additional file 2: Table S1) with a four bp window size, a required window quality of 15 and a read minimum length of 40 bp for the strand-specific paired-end development reads and 30 bp for the single-end adult reads. For the development assembly, reads for which both mates of a pair survived the quality filtering were assembled in PE mode of Trinity version 2.4 [37]. We also assembled the adult data in SE mode of Trinity version 2.4 (Additional file 2: Table S3) using default settings [37].

For each assembly, sequences with high similarity were clustered together using CD-HIT-EST (CD-HIT version 4.6.4, [38]) with an identity threshold of 0.95. De novo assembled transcripts that had no blast hit against the *A. burtoni* reference transcriptome (blastn within BLAST+ version 2.6.0 [39], percentage identity threshold 0.95 and minimum query coverage of 0.8) nor against the *O. niloticus* transcriptome (same settings) or the UniprotKB database (blastx within BLAST+ version 2.6.0 [39], default settings) were blasted against bacteria, archea, virus and fungi NCBI databases (September 2017) with an identity threshold of 0.95 and a query coverage cut-off of 0.5 to identify potential contaminants (blastx within BLAST+ version 2.6.0 [39]).

We also included transcripts assembled in a previous study [27]. From those, we identified transcripts not already present in the reference genome annotation by blasting them against the reference *A. burtoni* transcriptome with a blastn identity threshold of 0.95 and a query coverage of 0.8 (BLAST+ version 2.6.0 [39]). These transcripts were then blasted (blastx within BLAST+ 2.6.0 [39]) against the bacteria, the archea, the virus and the fungi NCBI database (September 2017) with an identity threshold of 0.95 and query coverage of 0.5 to remove potential contaminant transcripts. The de novo developmental assembly, the de novo adult assembly [26] and the transcripts absent from the *A. burtoni* reference transcriptome from [27] were then combined into a hybrid assembly (Additional file 2: Table S4). Sequences with high similarity were clustered together using CD-HIT-EST (CD-HIT version 4.6.4 [38]) with an identity threshold of 95%. In order to identify transcripts that are not present in the already existing *A. burtoni* genome annotation, we mapped the de novo transcripts to the reference genome with GMAP under default settings (GMAP-GSNAP version 2017-08-15, [40]). Transcripts that did not map to the genome (Fig. 1) were subsequently mapped against the four other available cichlid genomes [16] with GMAP (GMAP-GSNAP version 2017-08-15 [40]) under default settings (Fig. 2).

### Genome annotation improvement and transcript abundance

We identified transcripts missing from the reference genome annotation by using StringTie version 1.3.3 [41] as described in the following: We first mapped the developmental series trimmed reads to the reference *A. burtoni* genome with STAR version 2.5.2a [42] (−-outFilterMultimapxNmax 1 --outFilterMatchNminOverLread 0.4 --outFilterScoreMinOverLread 0.4). We then used the BAM outputs as input for StringTie version 1.3.3 [41] under default settings and the existing *A. burtoni* annotation file (GCF_000239415.1_AstBur1.0_genomic.gff) as guideline. The improved GTF file produced by StringTie and the reference *A. burtoni* genome were then used to construct a new reference FASTA file with the function gffread within Cufflinks version 2.2.1 [43]. The resulting FASTA file was used as an improved version of the *A. burtoni* reference transcriptome as it grouped transcripts that were already present in the annotation file and transcripts that were newly annotated. To summarize the annotation changes, we used gffcompare within Cufflinks version 2.2.1 [43] (statistics in Additional file 2: Table S2). We assessed the completeness of this transcriptome annotation in comparison to the existing annotation with BUSCO version 3.2.2 [44, 45] using actinopterygii_odb9 as lineage dataset and zebrafish as reference species as integrated in BUSCO. We next combined this transcriptome file with our de novo assembly using CD-HIT-EST (CD-HIT version 4.6.4 [38]). All resulting transcripts were then blasted against the non-redundant (nr) blast database (October 2017) to assign putative gene identities based on homology with blastx BLAST+ version 2.6.0 [39] (default settings, e-value cut-off of 0.001). Transdecoder version 3.0.1 [46] was used to identify coding regions within the transcripts. Abundance estimation of each transcript was defined by mapping the reads of each developmental sample to the final custom reference transcripts with bowtie2 (version 2.2.9) within RSEM as part of Trinity version 2.4 [37].

### Transcriptome expression and transcript characterization

The global patterns of gene expression differences were represented with the plotPCA function from the DESeq2 (version 3.4.2) R package [47] based on the transcript count data generated with RSEM imported with tximport version 1.4.0 [48] into DESeq2 version 3.4.2 [47] following the developers suggestions for transcripts. Transcripts were considered as expressed if they had at least a count of three in a minimum of three conditions. ‘Regularized log’ (rlog) transformation was used to minimize differences between samples. In order to represent one transcript expression distribution per condition, the replicates of each condition were grouped by calculating the median of expression (Fig. 4c). For better visualization, outliers were removed (Fig. 4d) when generating boxplots by excluding data points, which lay beyond the extremes of the whiskers with upper whisker = min(max(x), upper quartile + 1.5 * inter quartile range) and lower whisker = max(min(x), lowerquartile – 1.5 * inter quartile range). Transcripts were functionally annotated with Blast2GO version 5.1 [49] based on the blast output against the nr database (see above) with default settings. All GO enrichment graphs and all GO enrichment tables were produced within Blast2GO version 5.1 [49]. Enrichment analyses were run within Blast2GO using a two-tailed Fisher’s Exact Test with the full transcriptome as background set. GO terms of enrichment test outcomes were reduced to the most specific GO terms except for the GO analysis of transcripts in gene expression profiles (Fig. 6b), which grouped few transcripts and where hence non-reduced GO terms were kept.

### Gene expression specificity

To investigate transcripts that are only expressed in a certain group of samples (e.g. all trunk samples or at a certain time point, Fig. 5), we compared the normalized RSEM counts (see above) for specific conditions. A transcript was considered as condition-specific if its median of expression within the condition of interest was of at least five counts and its median of expression in all other condition smaller than five counts.

### Differential gene expression analysis

We performed pairwise comparisons of gene expression for all time points within the two body parts (8 vs 14 dpf, 8 dpf vs 20 dpf and 14 dpf vs 20 dpf, Additional file 2: Tables S7 and S8) within DESeq2 version 3.4.2. As for the PCA, transcripts were only kept if they had at least a count of three in a minimum of three conditions (final number of expressed transcripts after this filtering was 41,264). We considered transcripts differentially expressed below an adjusted *p*-value (FDR) of 0.05. To generate an overview of drastic changes in expression, we kept only transcripts that had a log2folchange > 1. Next, we intersected these pairwise gene expression comparisons and subsequently classified transcripts into eight possible and occurring expression profiles (Additional file 2: Table S8).

### Positive selection

To identify genes putatively under positive selection we used PosiGene version 0.1 [50] on a phylogenetic tree including *A. burtoni*, the Nile tilapia *O. niloticus* and the zebrafish *Danio rerio* as outgroup (Additional file 1: Figure S22). The input sequences were the hybrid de novo assembly for *A. burtoni* and all RefSeq mRNA sequences for *O. niloticus* and *D. rerio* (February 2018). Due to the close relatedness of *A. burtoni* and *O. niloticus*, *O. nilotiucs* was chosen as the “anchor species” (−as) and as reference for ortholog assignment (−rs) for PosiGene. Positive selection was tested on the branch leading to *A. burtoni* (−ts). We applied a sequence identity threshold of minimum 60% and a blast threshold of 0.001. PosiGene could construct three-species alignments and test for positive selection for 2756 transcripts of *O. niloticus*, corresponding to 1151 gene IDs.

### Gene co-expression network construction and clustering analysis

To identify interactions between genes related to a particular developmental stage (8, 14 and 20 dpf) or a body part (head, trunk), we constructed weighted gene co-expression networks with the R package WGCNA version 1.61.88 [51] following the signed network procedure described by Hilliard et al. [52]. Prior to network construction, we removed transcripts with extremely low expression levels, which we considered as noise (present in less than three samples with less than three counts per sample, see above and Additional file 1: Figure S2B). We applied a WGCNA workflow that was previously developed for experiments with limited sample sizes and comparable to our own set-up [53]. In this workflow, an iterative re-clustering approach is applied to obtain stable gene clusters. The soft powers for all rounds of re-clustering were between 14 and 16 (with 16 being the most frequent). Transcripts within modules with a module membership threshold below 0.5 were removed after each iteration (Additional file 1: Figure S27).

To select the most stable transcript network and validate the effectiveness of the re-clustering process, we tested for transcript module preservation. Module preservation was calculated as the percentage of transcripts of the previous clusters retained in the best matching new cluster as described in Rodenas-Cuadrado et al. [53] (Additional file 1: Figure S28).

### Module-trait relationship

Associations between traits and modules were determined using the calculated module eigengenes and correlating them with traits. Modules with the highest Pearson correlation values and p-value < 0.05 were considered significantly related to traits (Additional file 1: Figure S17).

### Gene ontology of WGCNA modules enrichment

Gene ontology enrichment analysis was done for transcripts within modules against the full transcriptome as background within Blast2GO version 5.1 as described above. To access main functions, we reduced the obtained enriched GO terms to GO classes using the GO slim vocabulary in the web tool CateGOrizer [54]. Relative contribution of GO enrichment term was visualized for up- and down-regulated transcripts and calculated separately per each GO category (Additional file 1: Figures S19 and S20).

## Additional files

Additional file 1: **Figure S1.** Images of developmental stages. **Figure S2.** Worflows for A) new assembly; B) gene expression. **Figure S3.** Genome annotation redefinition using StringTie. Legend courtesy of Geo Pertea of the Center for Computational Biology, Johns Hopkins University. **Figure S4.** De novo assembly construction. **Figure S5-S7.** GO enrichment of novel transcripts absent from other cichlid genomes (S5), transcription expression outliers (S6, A) head; B) trunk); sample-specific transcripts (S7 A) head, B) trunk, C) 8dpf, D) 14dpf, E) 20dpf), of transcripts matching expression profiles of Figure 6B (S8-S15). S8 profile1, S9 profile2, S10 profile3, S11 profile4, S12 profile5, S13 profile6, S14 profile7, S15 profile8. A) head; B) trunk. No enrichment was found for head for profile3. **Figure S16.** Analysis of network topology. A) Scale-free fit index as function of soft-thresholding power (x-axis). B) Network connectivities. **Figure S17.** Module-trait associations. Cells contain the corresponding Pearson correlation and *p*-value. **Figure S18.** GO enrichment in modules with a high positive correlation to traits. **Figure S19-S20.** GO classes per module for over- (S19) and under-represented (S20) GO categories. A), B) Biological Process; C), D) Cellular Component; E), F) Molecular Function. GOs were mapped to 127 slim GO ancestors. **Figure S21.** GO enrichment for modules with novel transcripts (green and black modules: Figure S18). **Figure S22.** Species-tree used for positive selection analysis. **Figure S23.** Expression of candidates. x-axis: condition; y-axis: read counts; dashed line: minimum threshold. **Figure S24-S25.** GO enrichment for transcripts under positive selection (S24), white and lightyellow modules (S25). **Figure S26.** Gene Significance (GS) versus module membership in modules containing candidates. A) White module, GS of 8dpf. B) Blue module, GS of head. C) Lightyellow module, GS of trunk. **Figure S27.** Iterative re-clustering of WGCNA modules. **Figure S28.** Module preservation during iterative re-clustering. Red points: outlier modules with poor percentage of module preservation. (PDF 2645 kb)

Additional file 2: **Table S1.** Sequencing data and processing. **Table S2.** Statistics for the improved genome annotation. **Table S3.** De novo assembly statistics. **Table S4.** Hybrid reference assembly summary statistics. **Table S5.** Species distribution in Blast annotation of the new transcriptome assembly. For each sequences, we exported the top blast hit and its species of origin. Species names as well as taxa are provided. **Table S6.** Transcript expression outliers in the trunk (Fig. 4c. **Table S7.** Number of differentially expressed transcripts for all pairwise comparisons. **Table S8.** Number of transcripts for each expression profile shown in Fig. 6b for each tissue type. **Table S9.** Number of transcripts in each gene expression network module. **Table S10.** Number of novel transcripts per gene expression module. **Table S11.** IDs of transcripts under positive selection. **Table S12.** GO annotations for fin development and genes within these categories for the lightyellow module. (XLSX 115 kb)

Additional file 3: **Data S1.** AstatotilapiaBurtoniNovelAnnotation.gtf improved annotation file to use with the current genome assembly. (GTF 153459 kb)

Additional file 4: **Data S2.** AstatotilapiaBurtoniNovelTranscripts.fasta, novel transcripts missing from the current *A. burtoni* reference genome in fasta format. (FASTA 7831 kb)

Additional file 5: **Data S3.** Gene Ontology, Blast, taxonomy and module annotation for hybrid reference assembly and corresponding gene/transcript IDs of the current *A. burtoni* reference genome. (TXT 48686 kb)

## Abbreviations

bp: Base pair
dpf: Day post-fertilization
GO: Gene ontology
ORF: Open reading frame
PCA: Principal component analysis
RNA-seq: RNA-sequencing
WGCNA: Weighted gene co-expression network analysis
