# Supplementary material for: Time matters! Developmental shift in gene expression between the head and the trunk region of the cichlid fish Astatotilapia burtoni
Source: BMC Genomics. 2019 Jan 14;20:39. doi: 10.1186/s12864-018-5321-6 (PMC6332847; doi:10.1186/s12864-018-5321-6)
Supplement: Supplementary file 1 — Figure S1. Images of developmental stages. Figure S2. Worflows for A) new assembly; B) gene expression. Figure S3. Genome annotation redefinition using StringTie. Legend courtesy of Geo Pertea of the Center for Computational Biology, Johns Hopkins University. Figure S4. De novo assembly construction. Figure S5-S7. GO enrichment of novel transcripts absent from other cichlid genomes (S5), transcription expression outliers (S6, A) head; B) trunk); sample-specific transcripts (S7 A) head, B) trunk, C) 8dpf, D) 14dpf, E) 20dpf), of transcripts matching expression profiles of Figure 6B (S8-S15). S8 profile1, S9 profile2, S10 profile3, S11 profile4, S12 profile5, S13 profile6, S14 profile7, S15 profile8. A) head; B) trunk. No enrichment was found for head for profile3. Figure S16. Analysis of network topology. A) Scale-free fit index as function of soft-thresholding power (x-axis). B) Network connectivities. Figure S17. Module-trait associations. Cells contain the corresponding Pearson correlation and p-value. Figure S18. GO enrichment in modules with a high positive correlation to traits. Figure S19-S20. GO classes per module for over- (S19) and under-represented (S20) GO categories. A), B) Biological Process; C), D) Cellular Component; E), F) Molecular Function. GOs were mapped to 127 slim GO ancestors. Figure S21. GO enrichment for modules with novel transcripts (green and black modules: Figure S18). Figure S22. Species-tree used for positive selection analysis. Figure S23. Expression of candidates. x-axis: condition; y-axis: read counts; dashed line: minimum threshold. Figure S24-S25. GO enrichment for transcripts under positive selection (S24), white and lightyellow modules (S25). Figure S26. Gene Significance (GS) versus module membership in modules containing candidates. A) White module, GS of 8dpf. B) Blue module, GS of head. C) Lightyellow module, GS of trunk. Figure S27. Iterative re-clustering of WGCNA modules. Figure S28. Module preservation during iterative r [file 12864_2018_5321_MOESM1_ESM.pdf]

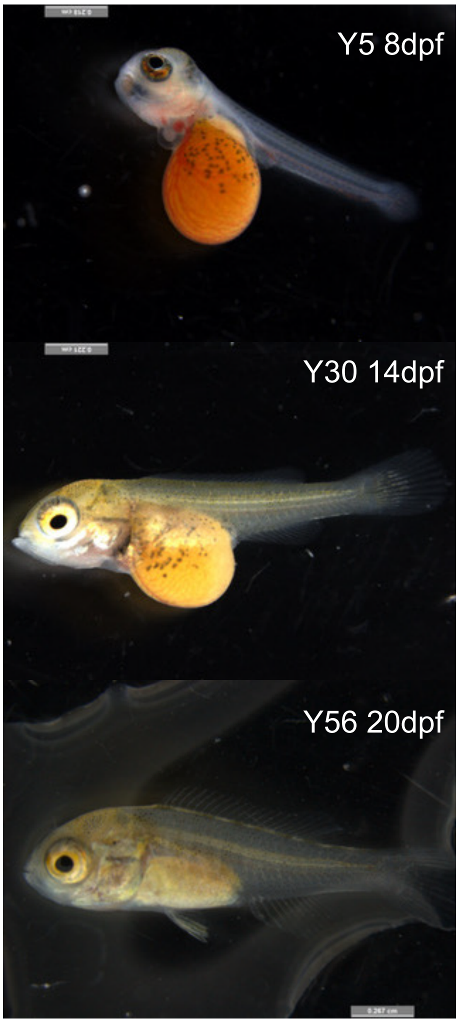

Figure S1

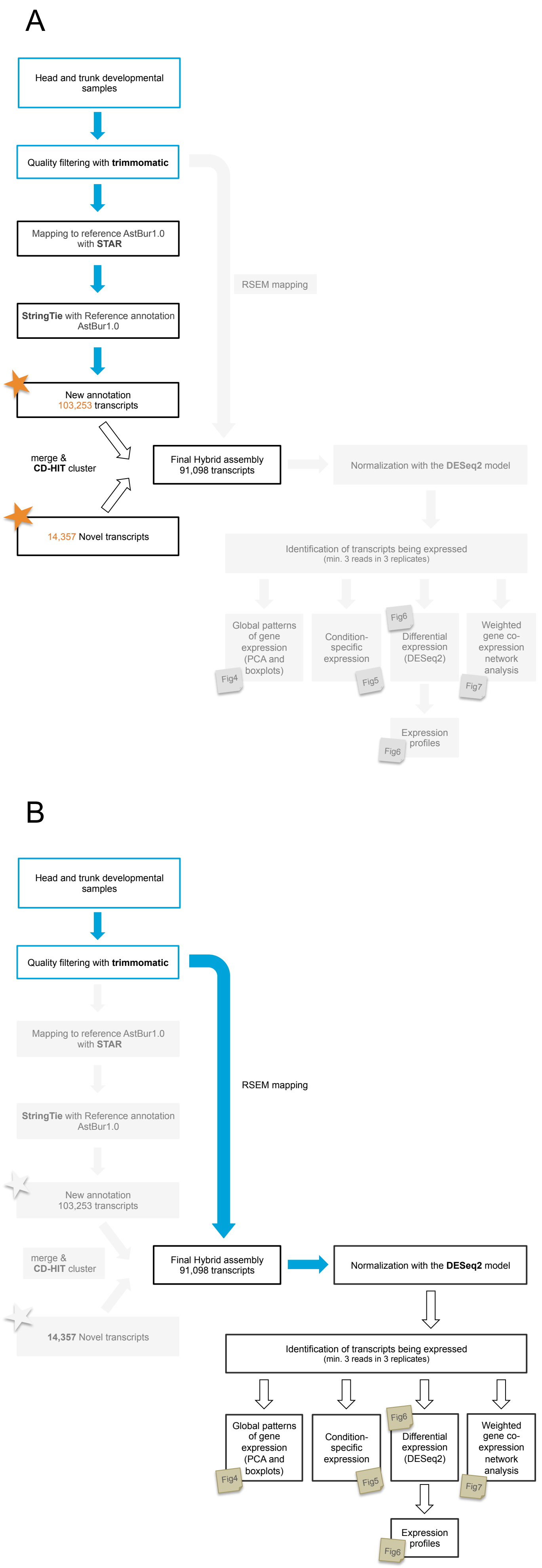
A
Head and trunk developmental samples
Quality filtering with trimmomatic
Mapping to reference AstBur1.0 with STAR
StringTie with Reference annotation AstBur1.0
New annotation 103,253 transcripts
merge & CD-HIT cluster
14,357 Novel transcripts
RSEM mapping
Final Hybrid assembly 91,098 transcripts
Normalization with the DESeq2 model
Identification of transcripts being expressed (min. 3 reads in 3 replicates)
Global patterns of gene expression (PCA and boxplots)
Fig4
Condition-specific expression
Fig5
Differential expression (DESeq2)
Fig6
Weighted gene co-expression network analysis
Fig7
Expression profiles
Fig6
B
Head and trunk developmental samples
Quality filtering with trimmomatic
Mapping to reference AstBur1.0 with STAR
StringTie with Reference annotation AstBur1.0
New annotation 103,253 transcripts
merge & CD-HIT cluster
14,357 Novel transcripts
RSEM mapping
Final Hybrid assembly 91,098 transcripts
Normalization with the DESeq2 model
Identification of transcripts being expressed (min. 3 reads in 3 replicates)
Global patterns of gene expression (PCA and boxplots)
Fig4
Condition-specific expression
Fig5
Differential expression (DESeq2)
Fig6
Weighted gene co-expression network analysis
Fig7
Expression profiles
Fig6

Figure S2

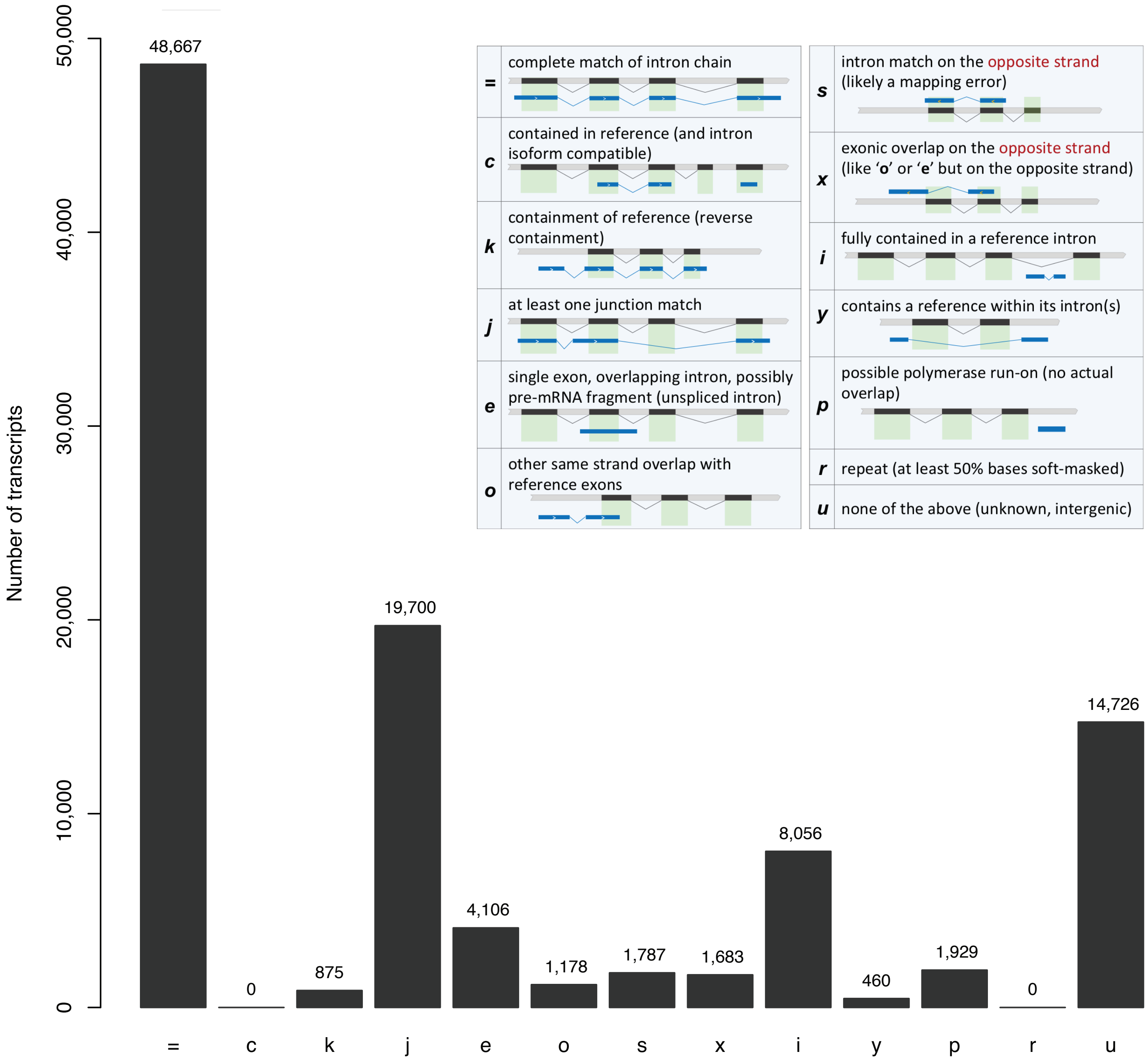

Number of transcripts
0
10,000
20,000
30,000
40,000
50,000
48,667
0
875
19,700
4,106
1,178
1,787
1,683
8,056
460
1,929
0
14,726
=
c
k
j
e
o
s
x
i
y
p
r
u
= complete match of intron chain
c contained in reference (and intron isoform compatible)
k containment of reference (reverse containment)
j at least one junction match
e single exon, overlapping intron, possibly pre-mRNA fragment (unspliced intron)
o other same strand overlap with reference exons
s intron match on the opposite strand (likely a mapping error)
x exonic overlap on the opposite strand (like 'o' or 'e' but on the opposite strand)
i fully contained in a reference intron
y contains a reference within its intron(s)
p possible polymerase run-on (no actual overlap)
r repeat (at least 50% bases soft-masked)
u none of the above (unknown, intergenic)

Figure S3

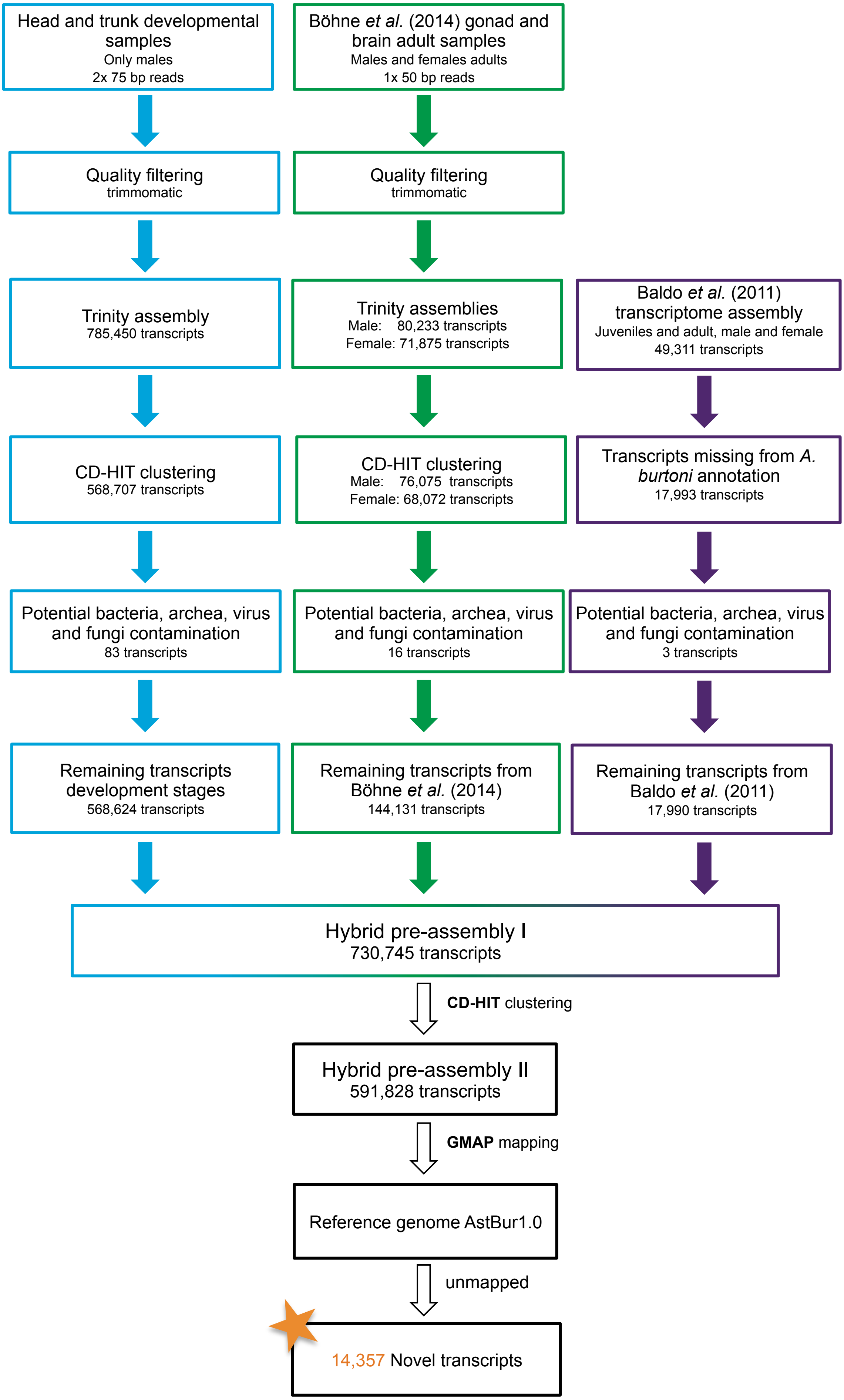
Head and trunk developmental samples
Only males
2x 75 bp reads
Böhne et al. (2014) gonad and brain adult samples
Males and females adults
1x 50 bp reads
Quality filtering
trimmomatic
Quality filtering
trimmomatic
Trinity assembly
785,450 transcripts
Trinity assemblies
Male: 80,233 transcripts
Female: 71,875 transcripts
Baldo et al. (2011) transcriptome assembly
Juveniles and adult, male and female
49,311 transcripts
CD-HIT clustering
568,707 transcripts
CD-HIT clustering
Male: 76,075 transcripts
Female: 68,072 transcripts
Transcripts missing from A. burtoni annotation
17,993 transcripts
Potential bacteria, archea, virus and fungi contamination
83 transcripts
Potential bacteria, archea, virus and fungi contamination
16 transcripts
Potential bacteria, archea, virus and fungi contamination
3 transcripts
Remaining transcripts development stages
568,624 transcripts
Remaining transcripts from Böhne et al. (2014)
144,131 transcripts
Remaining transcripts from Baldo et al. (2011)
17,990 transcripts
Hybrid pre-assembly I
730,745 transcripts
CD-HIT clustering
Hybrid pre-assembly II
591,828 transcripts
GMAP mapping
Reference genome AstBur1.0
unmapped
14,357 Novel transcripts

Figure S4

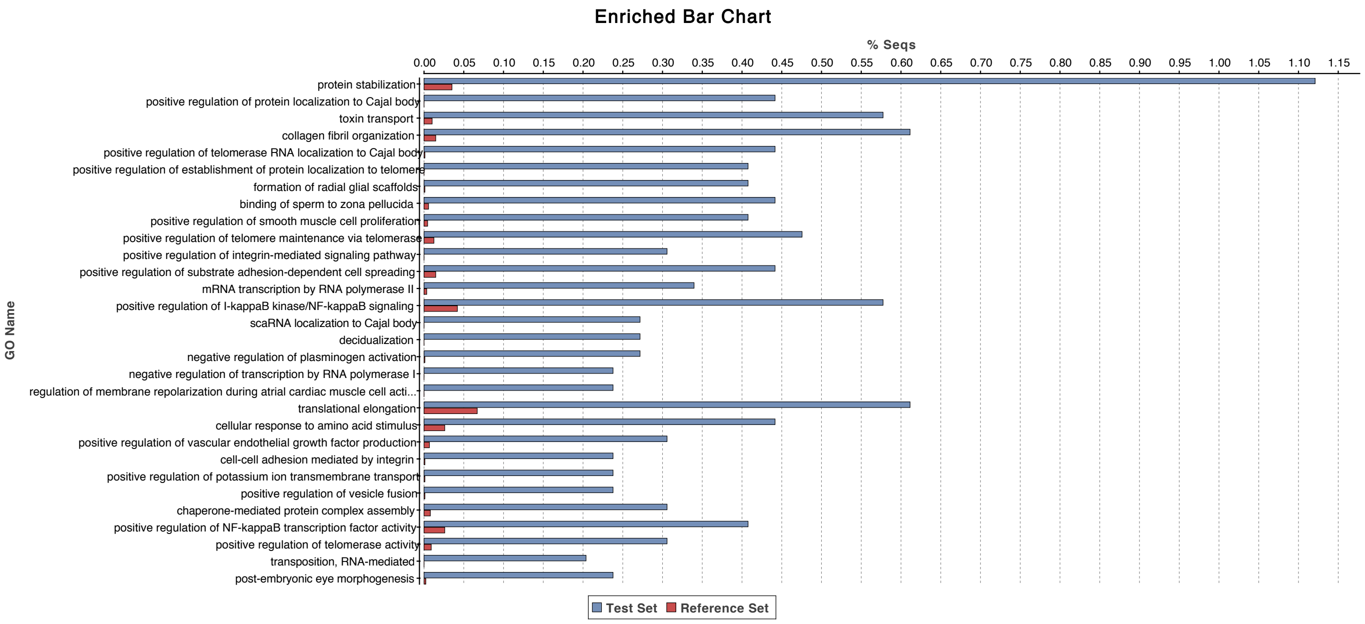

Enriched Bar Chart
% Seqs
0.00
0.05
0.10
0.15
0.20
0.25
0.30
0.35
0.40
0.45
0.50
0.55
0.60
0.65
0.70
0.75
0.80
0.85
0.90
0.95
1.00
1.05
1.10
1.15
GO Name
protein stabilization
positive regulation of protein localization to Cajal body
toxin transport
collagen fibril organization
positive regulation of telomerase RNA localization to Cajal body
positive regulation of establishment of protein localization to telomere
formation of radial glial scaffolds
binding of sperm to zona pellucida
positive regulation of smooth muscle cell proliferation
positive regulation of telomere maintenance via telomerase
positive regulation of integrin-mediated signaling pathway
positive regulation of substrate adhesion-dependent cell spreading
mRNA transcription by RNA polymerase II
positive regulation of I-kappaB kinase/NF-kappaB signaling
scaRNA localization to Cajal body
decidualization
negative regulation of plasminogen activation
negative regulation of transcription by RNA polymerase I
regulation of membrane repolarization during atrial cardiac muscle cell acti...
translational elongation
cellular response to amino acid stimulus
positive regulation of vascular endothelial growth factor production
cell-cell adhesion mediated by integrin
positive regulation of potassium ion transmembrane transport
positive regulation of vesicle fusion
chaperone-mediated protein complex assembly
positive regulation of NF-kappaB transcription factor activity
positive regulation of telomerase activity
transposition, RNA-mediated
post-embryonic eye morphogenesis
Test Set
Reference Set

Figure S5

A

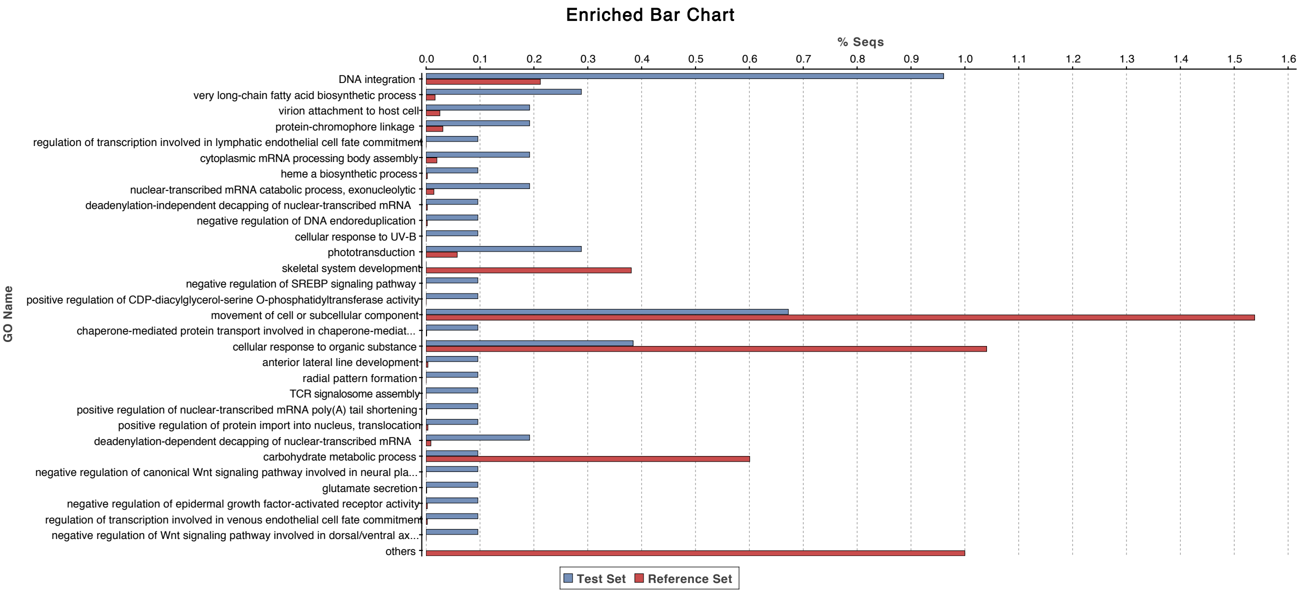

Enriched Bar Chart
% Seqs
GO Name
DNA integration
very long-chain fatty acid biosynthetic process
virion attachment to host cell
protein-chromophore linkage
regulation of transcription involved in lymphatic endothelial cell fate commitment
cytoplasmic mRNA processing body assembly
heme a biosynthetic process
nuclear-transcribed mRNA catabolic process, exonucleolytic
deadenylation-independent decapping of nuclear-transcribed mRNA
negative regulation of DNA endoreduplication
cellular response to UV-B
phototransduction
skeletal system development
negative regulation of SREBP signaling pathway
positive regulation of CDP-diacylglycerol-serine O-phosphatidyltransferase activity
movement of cell or subcellular component
chaperone-mediated protein transport involved in chaperone-mediat...
cellular response to organic substance
anterior lateral line development
radial pattern formation
TCR signalosome assembly
positive regulation of nuclear-transcribed mRNA poly(A) tail shortening
positive regulation of protein import into nucleus, translocation
deadenylation-dependent decapping of nuclear-transcribed mRNA
carbohydrate metabolic process
negative regulation of canonical Wnt signaling pathway involved in neural pla...
glutamate secretion
negative regulation of epidermal growth factor-activated receptor activity
regulation of transcription involved in venous endothelial cell fate commitment
negative regulation of Wnt signaling pathway involved in dorsal/ventral ax...
others
Test Set
Reference Set

B

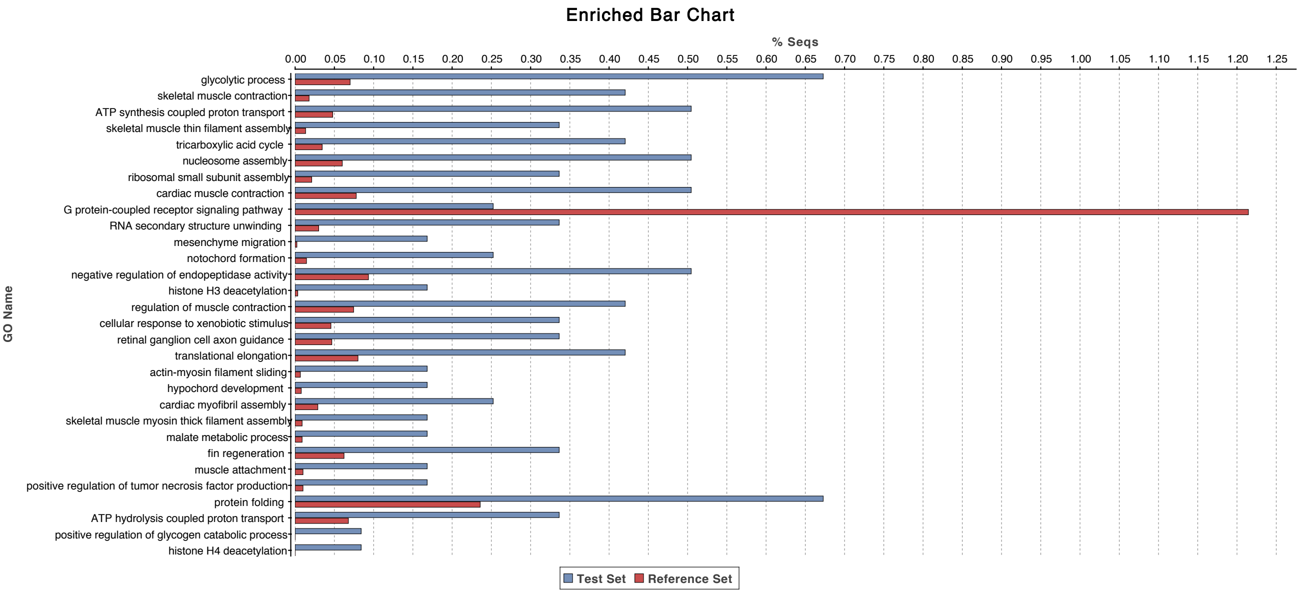

Enriched Bar Chart
% Seqs
GO Name
glycolytic process
skeletal muscle contraction
ATP synthesis coupled proton transport
skeletal muscle thin filament assembly
tricarboxylic acid cycle
nucleosome assembly
ribosomal small subunit assembly
cardiac muscle contraction
G protein-coupled receptor signaling pathway
RNA secondary structure unwinding
mesenchyme migration
notochord formation
negative regulation of endopeptidase activity
histone H3 deacetylation
regulation of muscle contraction
cellular response to xenobiotic stimulus
retinal ganglion cell axon guidance
translational elongation
actin-myosin filament sliding
hypochord development
cardiac myofibril assembly
skeletal muscle myosin thick filament assembly
malate metabolic process
fin regeneration
muscle attachment
positive regulation of tumor necrosis factor production
protein folding
ATP hydrolysis coupled proton transport
positive regulation of glycogen catabolic process
histone H4 deacetylation
Test Set
Reference Set

Figure S6

A

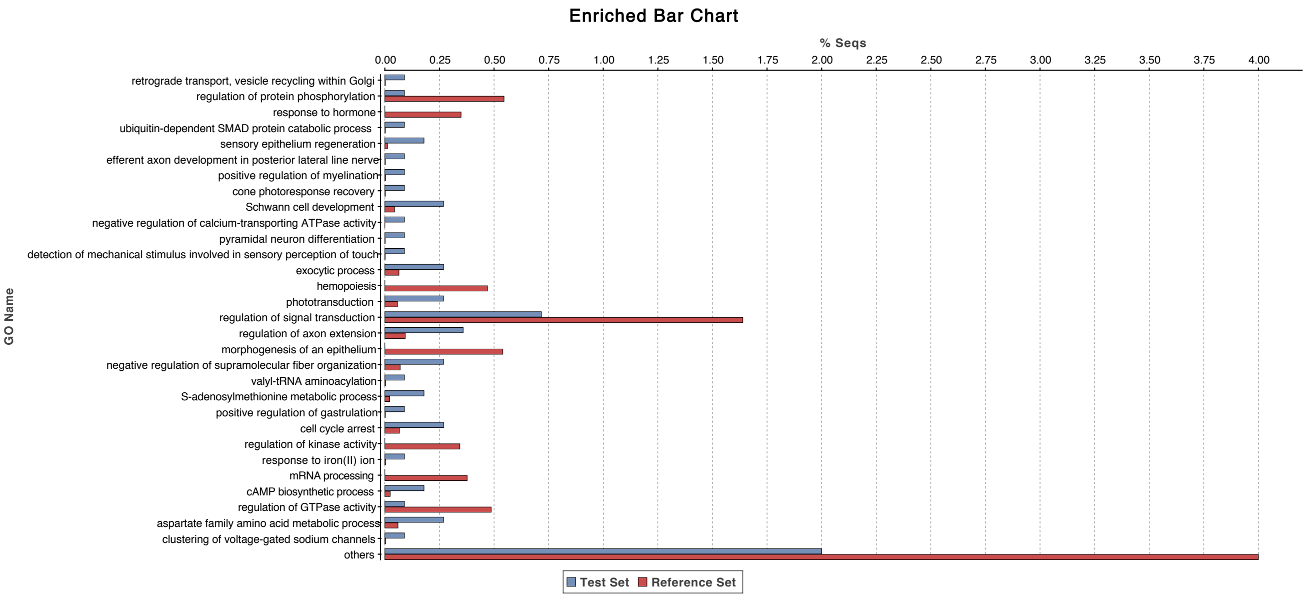
Enriched Bar Chart
% Seqs
0.00
0.25
0.50
0.75
1.00
1.25
1.50
1.75
2.00
2.25
2.50
2.75
3.00
3.25
3.50
3.75
4.00
GO Name
retrograde transport, vesicle recycling within Golgi
regulation of protein phosphorylation
response to hormone
ubiquitin-dependent SMAD protein catabolic process
sensory epithelium regeneration
efferent axon development in posterior lateral line nerve
positive regulation of myelination
cone photoresponse recovery
Schwann cell development
negative regulation of calcium-transporting ATPase activity
pyramidal neuron differentiation
detection of mechanical stimulus involved in sensory perception of touch
exocytic process
hemopoiesis
phototransduction
regulation of signal transduction
regulation of axon extension
morphogenesis of an epithelium
negative regulation of supramolecular fiber organization
valyl-tRNA aminoacylation
S-adenosylmethionine metabolic process
positive regulation of gastrulation
cell cycle arrest
regulation of kinase activity
response to iron(II) ion
mRNA processing
cAMP biosynthetic process
regulation of GTPase activity
aspartate family amino acid metabolic process
clustering of voltage-gated sodium channels
others
Test Set
Reference Set

B

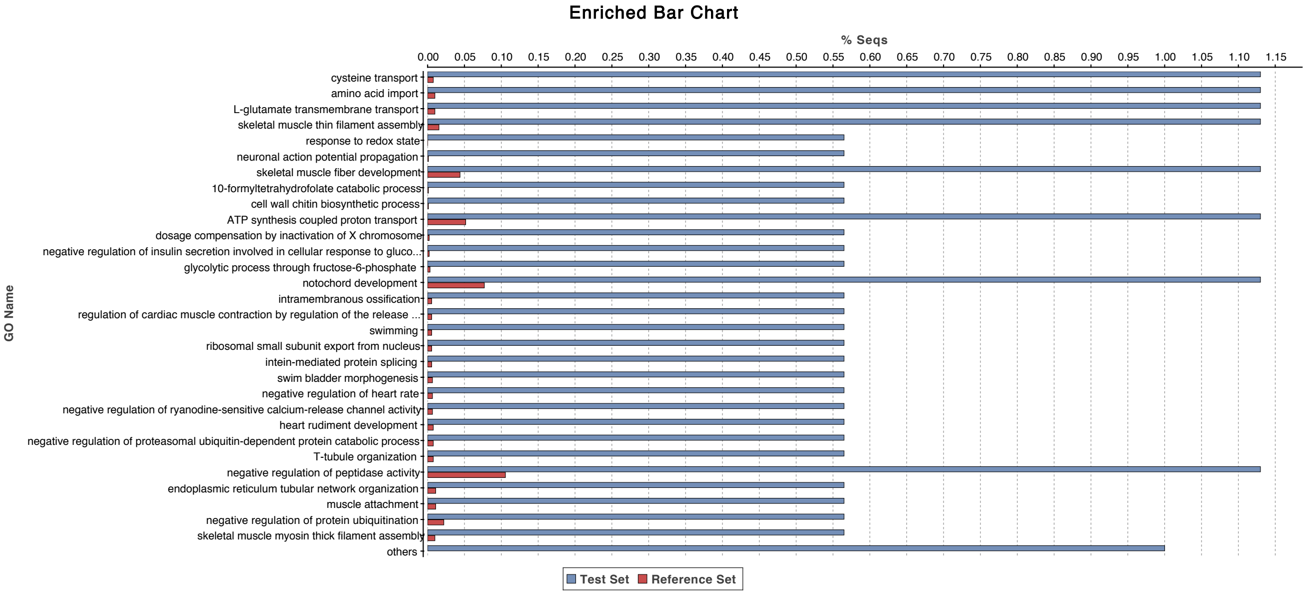
Enriched Bar Chart
% Seqs
0.00
0.05
0.10
0.15
0.20
0.25
0.30
0.35
0.40
0.45
0.50
0.55
0.60
0.65
0.70
0.75
0.80
0.85
0.90
0.95
1.00
1.05
1.10
1.15
GO Name
cysteine transport
amino acid import
L-glutamate transmembrane transport
skeletal muscle thin filament assembly
response to redox state
neuronal action potential propagation
skeletal muscle fiber development
10-formyltetrahydrofolate catabolic process
cell wall chitin biosynthetic process
ATP synthesis coupled proton transport
dosage compensation by inactivation of X chromosome
negative regulation of insulin secretion involved in cellular response to gluco...
glycolytic process through fructose-6-phosphate
notochord development
intramembranous ossification
regulation of cardiac muscle contraction by regulation of the release ...
swimming
ribosomal small subunit export from nucleus
intein-mediated protein splicing
swim bladder morphogenesis
negative regulation of heart rate
negative regulation of ryanodine-sensitive calcium-release channel activity
heart rudiment development
negative regulation of proteasomal ubiquitin-dependent protein catabolic process
T-tubule organization
negative regulation of peptidase activity
endoplasmic reticulum tubular network organization
muscle attachment
negative regulation of protein ubiquitination
skeletal muscle myosin thick filament assembly
others
Test Set
Reference Set

Figure S7

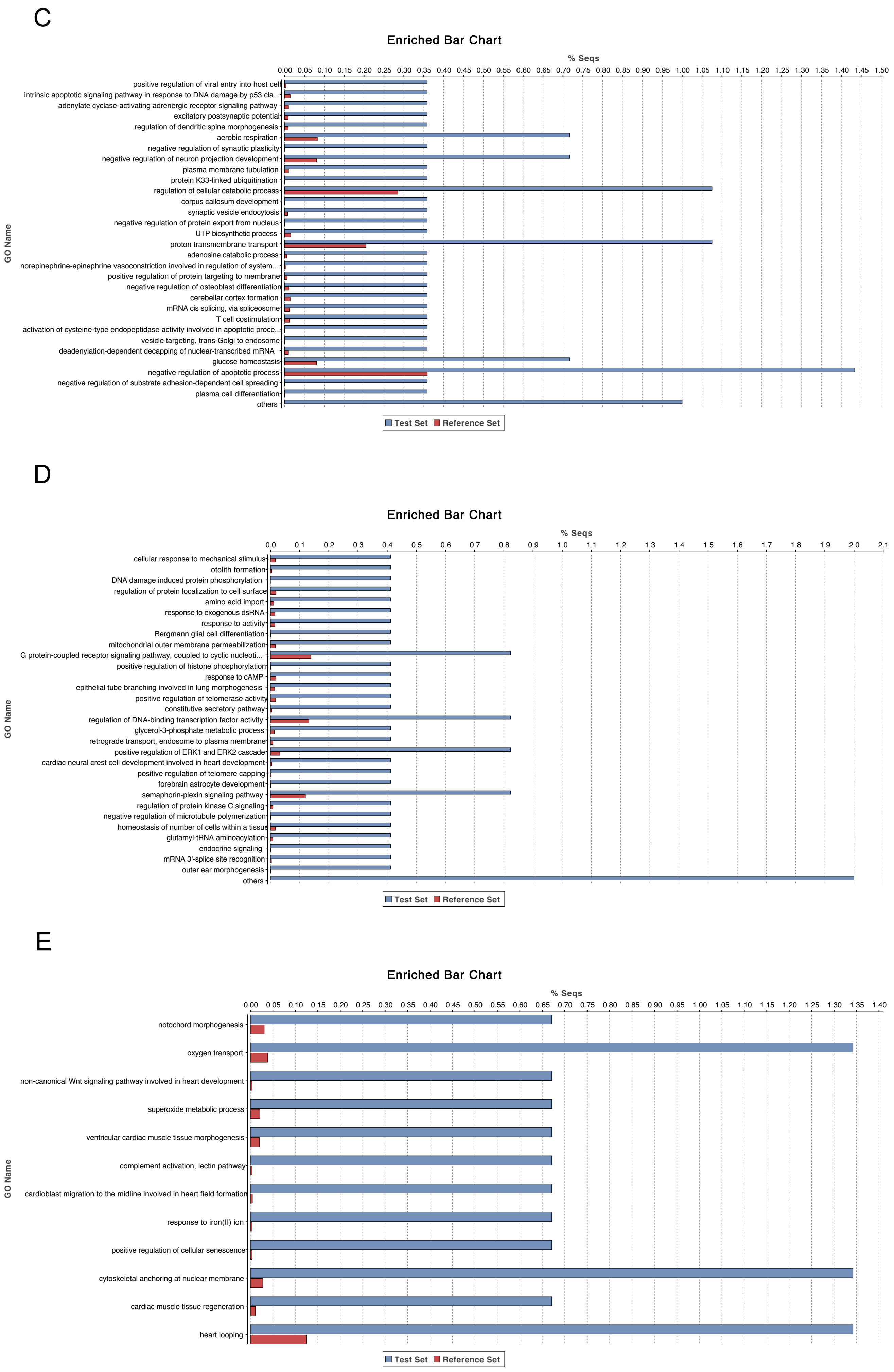
C
Enriched Bar Chart
% Seqs
0.00 0.05 0.10 0.15 0.20 0.25 0.30 0.35 0.40 0.45 0.50 0.55 0.60 0.65 0.70 0.75 0.80 0.85 0.90 0.95 1.00 1.05 1.10 1.15 1.20 1.25 1.30 1.35 1.40 1.45 1.50
GO Name
positive regulation of viral entry into host cell
intrinsic apoptotic signaling pathway in response to DNA damage by p53 cla...
adenylate cyclase-activating adrenergic receptor signaling pathway
excitatory postsynaptic potential
regulation of dendritic spine morphogenesis
aerobic respiration
negative regulation of synaptic plasticity
negative regulation of neuron projection development
plasma membrane tubulation
protein K33-linked ubiquitination
regulation of cellular catabolic process
corpus callosum development
synaptic vesicle endocytosis
negative regulation of protein export from nucleus
UTP biosynthetic process
proton transmembrane transport
adenosine catabolic process
norepinephrine-epinephrine vasoconstriction involved in regulation of system...
positive regulation of protein targeting to membrane
negative regulation of osteoblast differentiation
cerebellar cortex formation
mRNA cis splicing, via spliceosome
T cell costimulation
activation of cysteine-type endopeptidase activity involved in apoptotic proce...
vesicle targeting, trans-Golgi to endosome
deadenylation-dependent decapping of nuclear-transcribed mRNA
glucose homeostasis
negative regulation of apoptotic process
negative regulation of substrate adhesion-dependent cell spreading
plasma cell differentiation
others
Test Set
Reference Set
D
Enriched Bar Chart
% Seqs
0.0 0.1 0.2 0.3 0.4 0.5 0.6 0.7 0.8 0.9 1.0 1.1 1.2 1.3 1.4 1.5 1.6 1.7 1.8 1.9 2.0 2.1
GO Name
cellular response to mechanical stimulus
otolith formation
DNA damage induced protein phosphorylation
regulation of protein localization to cell surface
amino acid import
response to exogenous dsRNA
response to activity
Bergmann glial cell differentiation
mitochondrial outer membrane permeabilization
G protein-coupled receptor signaling pathway, coupled to cyclic nucleoti...
positive regulation of histone phosphorylation
response to cAMP
epithelial tube branching involved in lung morphogenesis
positive regulation of telomerase activity
constitutive secretory pathway
regulation of DNA-binding transcription factor activity
glycerol-3-phosphate metabolic process
retrograde transport, endosome to plasma membrane
positive regulation of ERK1 and ERK2 cascade
cardiac neural crest cell development involved in heart development
positive regulation of telomere capping
forebrain astrocyte development
semaphorin-plexin signaling pathway
regulation of protein kinase C signaling
negative regulation of microtubule polymerization
homeostasis of number of cells within a tissue
glutamyl-tRNA aminoacylation
endocrine signaling
mRNA 3'-splice site recognition
outer ear morphogenesis
others
Test Set
Reference Set
E
Enriched Bar Chart
% Seqs
0.00 0.05 0.10 0.15 0.20 0.25 0.30 0.35 0.40 0.45 0.50 0.55 0.60 0.65 0.70 0.75 0.80 0.85 0.90 0.95 1.00 1.05 1.10 1.15 1.20 1.25 1.30 1.35 1.40
GO Name
notochord morphogenesis
oxygen transport
non-canonical Wnt signaling pathway involved in heart development
superoxide metabolic process
ventricular cardiac muscle tissue morphogenesis
complement activation, lectin pathway
cardioblast migration to the midline involved in heart field formation
response to iron(II) ion
positive regulation of cellular senescence
cytoskeletal anchoring at nuclear membrane
cardiac muscle tissue regeneration
heart looping
Test Set
Reference Set

Figure S7

A

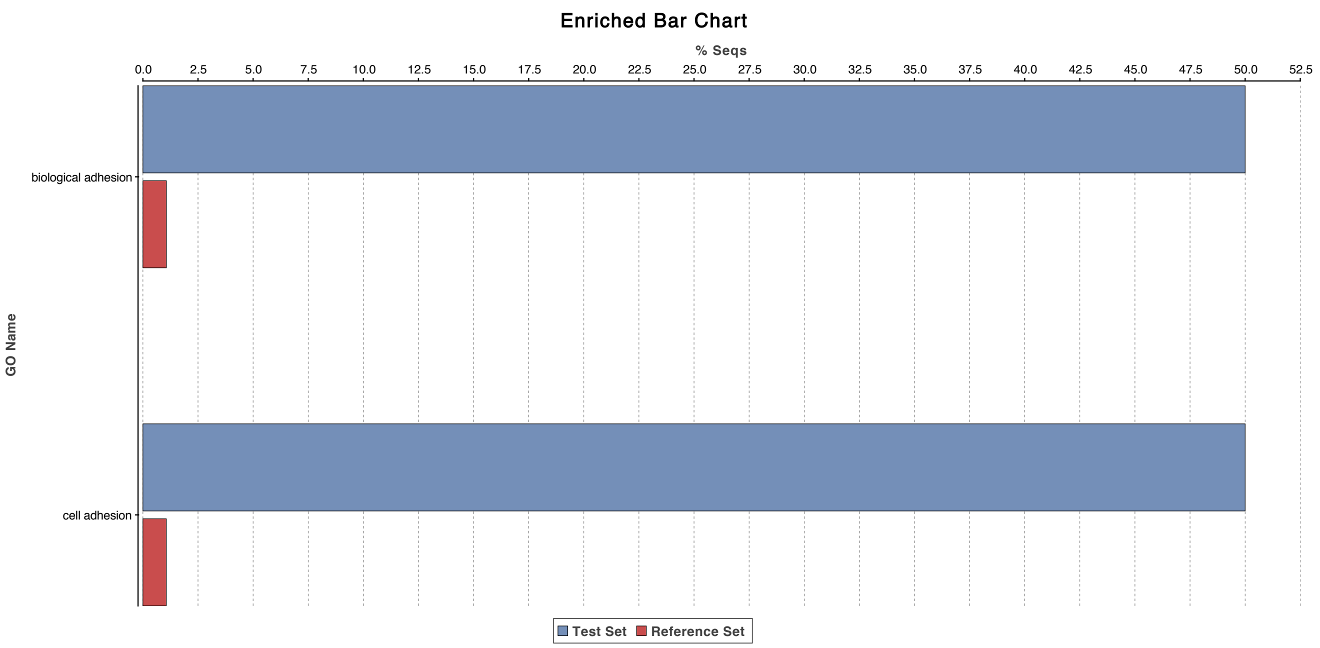

Enriched Bar Chart
% Seqs
0.0
2.5
5.0
7.5
10.0
12.5
15.0
17.5
20.0
22.5
25.0
27.5
30.0
32.5
35.0
37.5
40.0
42.5
45.0
47.5
50.0
52.5
GO Name
biological adhesion
cell adhesion
Test Set
Reference Set

B

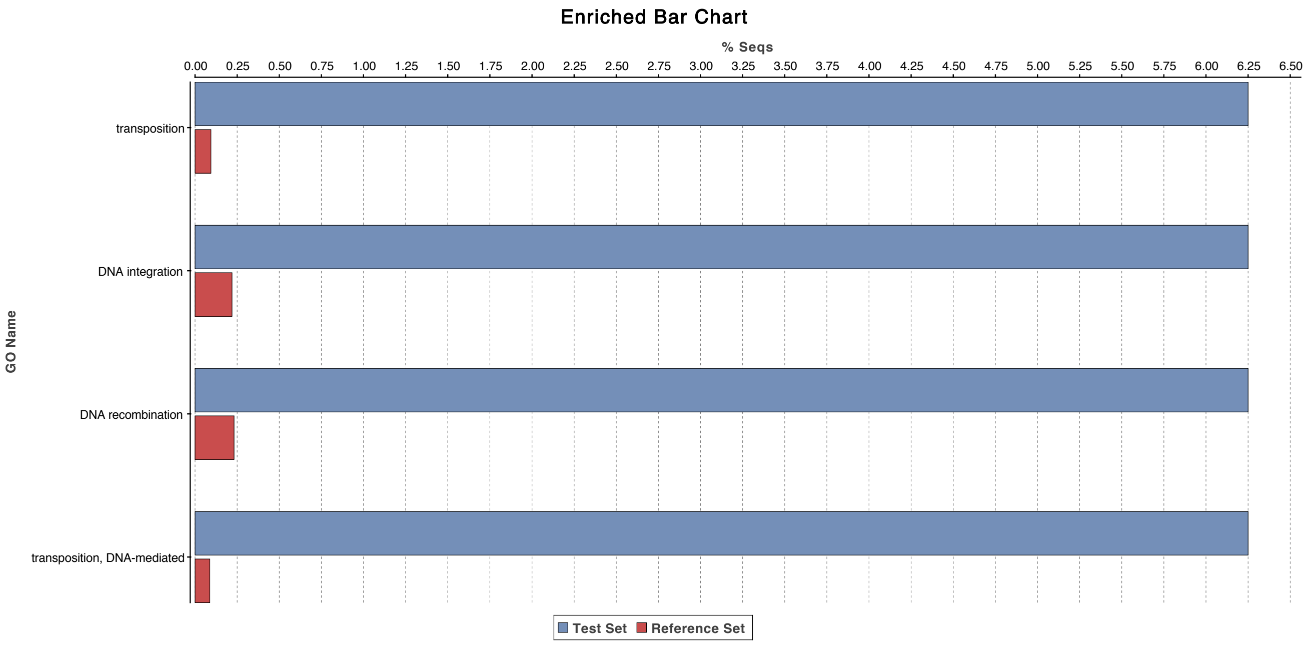

Enriched Bar Chart
% Seqs
0.00
0.25
0.50
0.75
1.00
1.25
1.50
1.75
2.00
2.25
2.50
2.75
3.00
3.25
3.50
3.75
4.00
4.25
4.50
4.75
5.00
5.25
5.50
5.75
6.00
6.25
6.50
GO Name
transposition
DNA integration
DNA recombination
transposition, DNA-mediated
Test Set
Reference Set

Figure S8

A

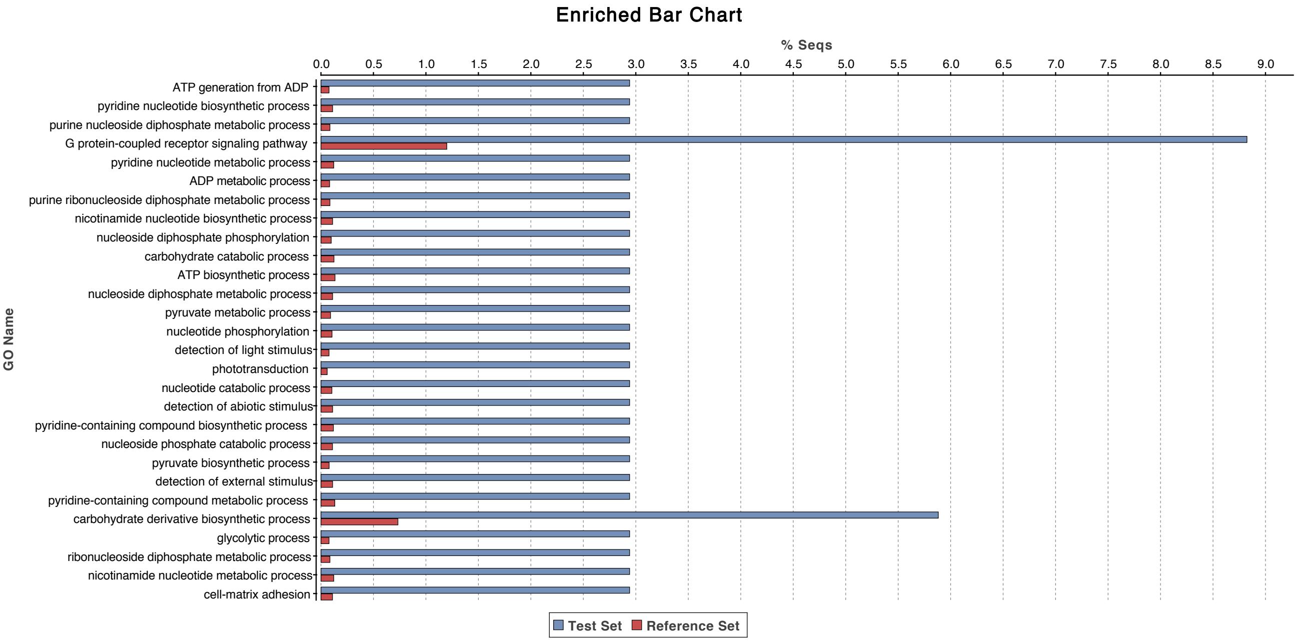

Enriched Bar Chart
% Seqs
0.0
0.5
1.0
1.5
2.0
2.5
3.0
3.5
4.0
4.5
5.0
5.5
6.0
6.5
7.0
7.5
8.0
8.5
9.0
GO Name
ATP generation from ADP
pyridine nucleotide biosynthetic process
purine nucleoside diphosphate metabolic process
G protein-coupled receptor signaling pathway
pyridine nucleotide metabolic process
ADP metabolic process
purine ribonucleoside diphosphate metabolic process
nicotinamide nucleotide biosynthetic process
nucleoside diphosphate phosphorylation
carbohydrate catabolic process
ATP biosynthetic process
nucleoside diphosphate metabolic process
pyruvate metabolic process
nucleotide phosphorylation
detection of light stimulus
phototransduction
nucleotide catabolic process
detection of abiotic stimulus
pyridine-containing compound biosynthetic process
nucleoside phosphate catabolic process
pyruvate biosynthetic process
detection of external stimulus
pyridine-containing compound metabolic process
carbohydrate derivative biosynthetic process
glycolytic process
ribonucleoside diphosphate metabolic process
nicotinamide nucleotide metabolic process
cell-matrix adhesion
Test Set
Reference Set

B

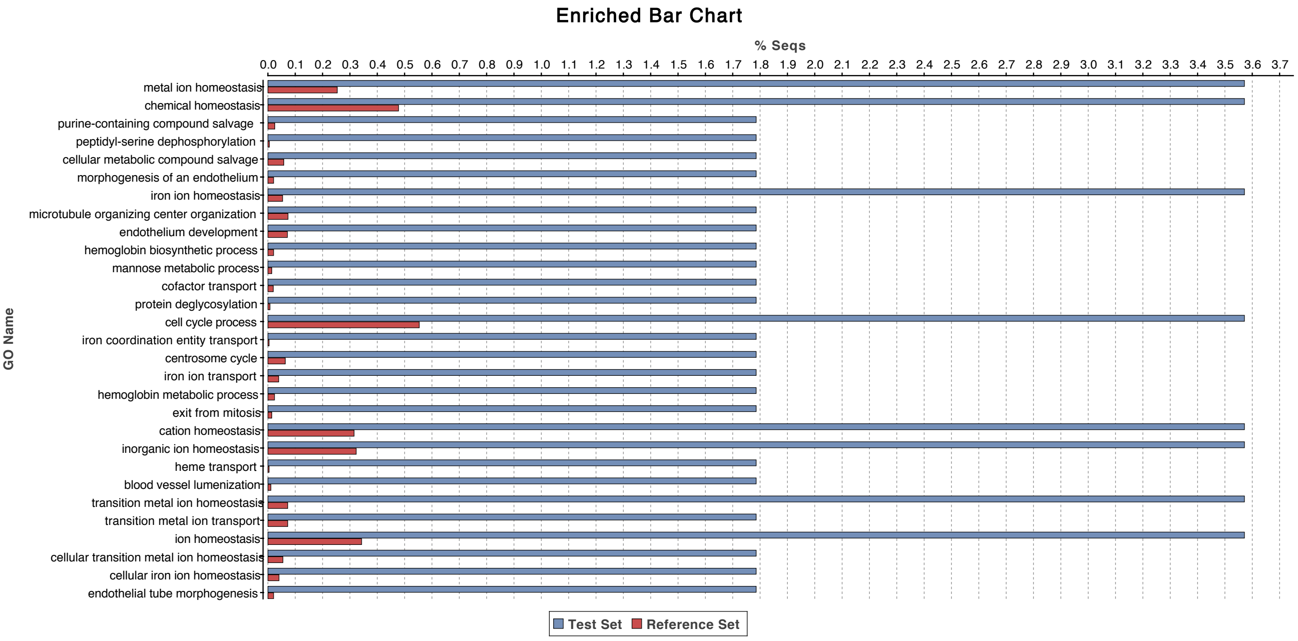

Enriched Bar Chart
% Seqs
0.0
0.1
0.2
0.3
0.4
0.5
0.6
0.7
0.8
0.9
1.0
1.1
1.2
1.3
1.4
1.5
1.6
1.7
1.8
1.9
2.0
2.1
2.2
2.3
2.4
2.5
2.6
2.7
2.8
2.9
3.0
3.1
3.2
3.3
3.4
3.5
3.6
3.7
GO Name
metal ion homeostasis
chemical homeostasis
purine-containing compound salvage
peptidyl-serine dephosphorylation
cellular metabolic compound salvage
morphogenesis of an endothelium
iron ion homeostasis
microtubule organizing center organization
endothelium development
hemoglobin biosynthetic process
mannose metabolic process
cofactor transport
protein deglycosylation
cell cycle process
iron coordination entity transport
centrosome cycle
iron ion transport
hemoglobin metabolic process
exit from mitosis
cation homeostasis
inorganic ion homeostasis
heme transport
blood vessel lumenization
transition metal ion homeostasis
transition metal ion transport
ion homeostasis
cellular transition metal ion homeostasis
cellular iron ion homeostasis
endothelial tube morphogenesis
Test Set
Reference Set

Figure S9

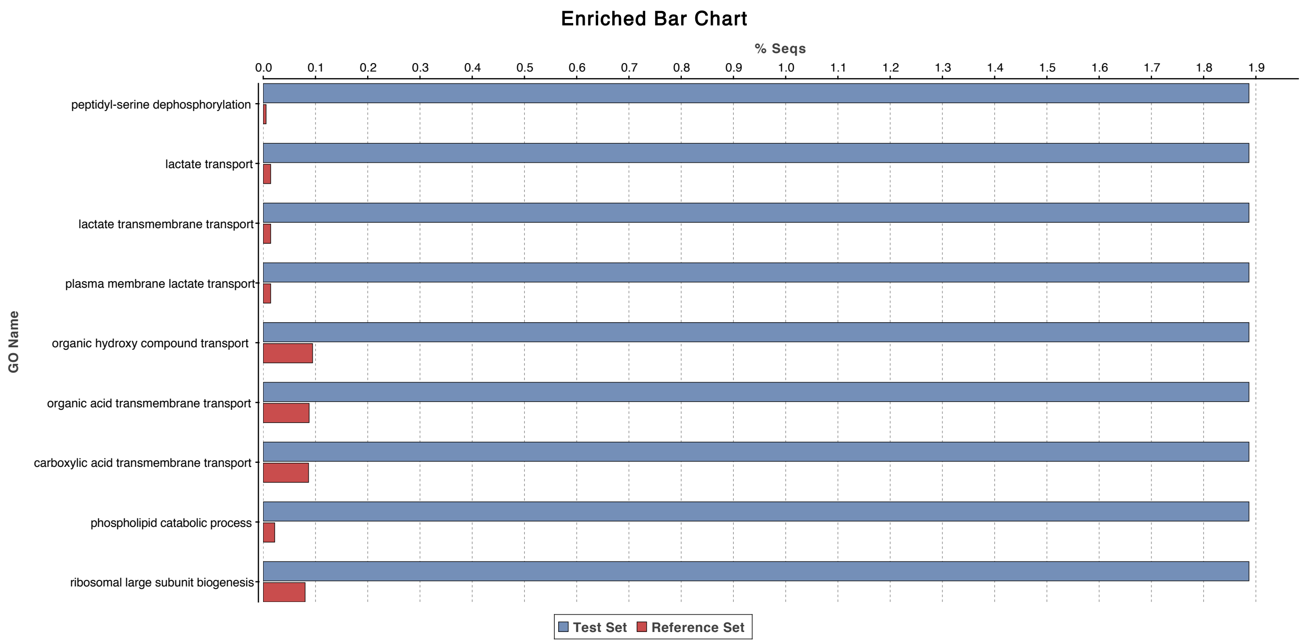

Enriched Bar Chart
% Seqs
0.0
0.1
0.2
0.3
0.4
0.5
0.6
0.7
0.8
0.9
1.0
1.1
1.2
1.3
1.4
1.5
1.6
1.7
1.8
1.9
GO Name
peptidyl-serine dephosphorylation
lactate transport
lactate transmembrane transport
plasma membrane lactate transport
organic hydroxy compound transport
organic acid transmembrane transport
carboxylic acid transmembrane transport
phospholipid catabolic process
ribosomal large subunit biogenesis
Test Set
Reference Set

Figure S10

A

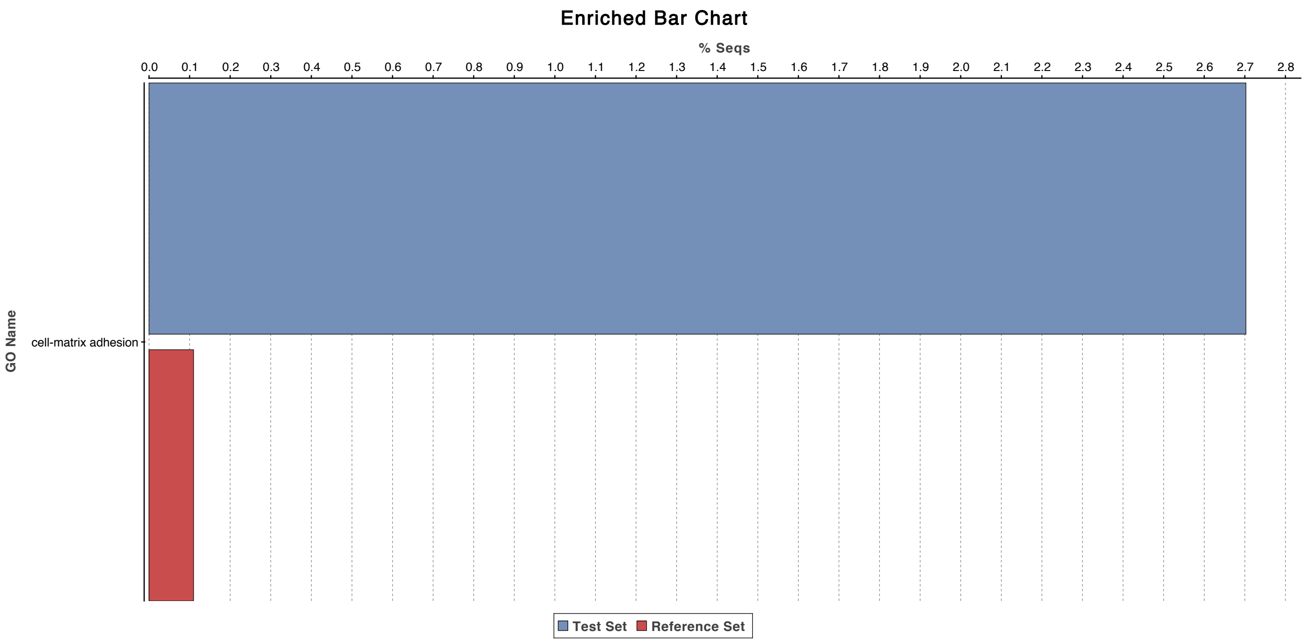

B

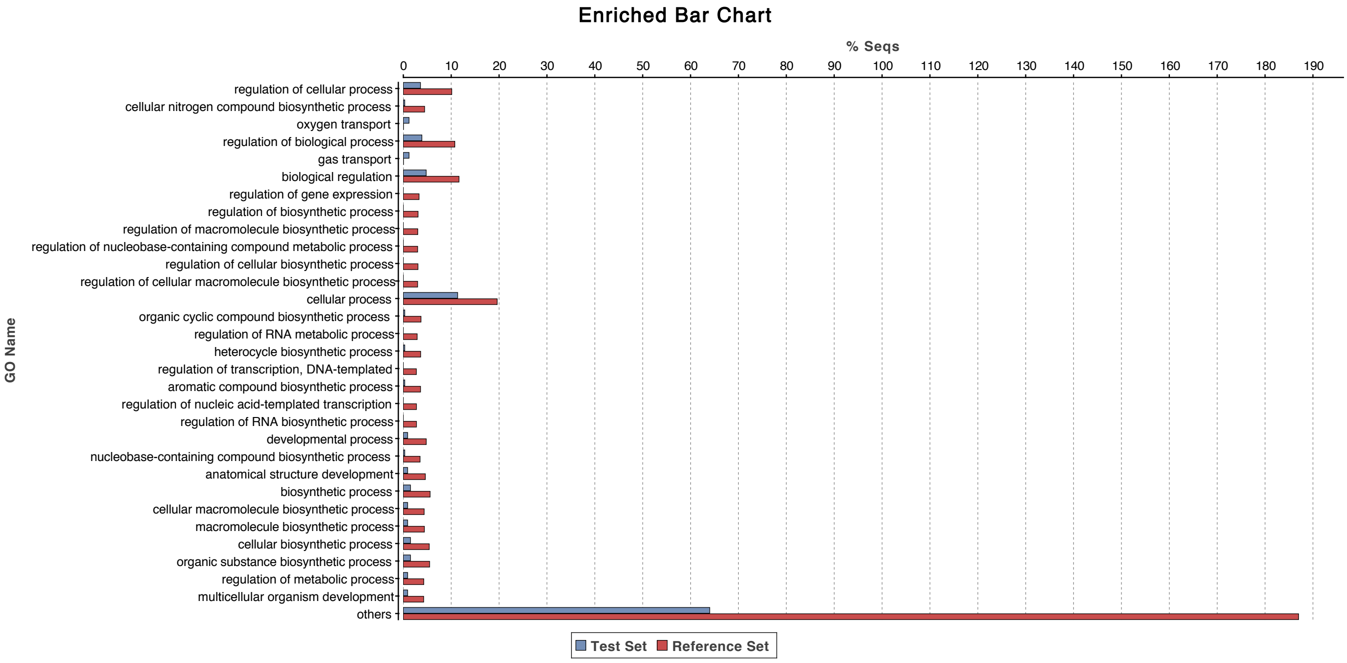

Figure S11

A

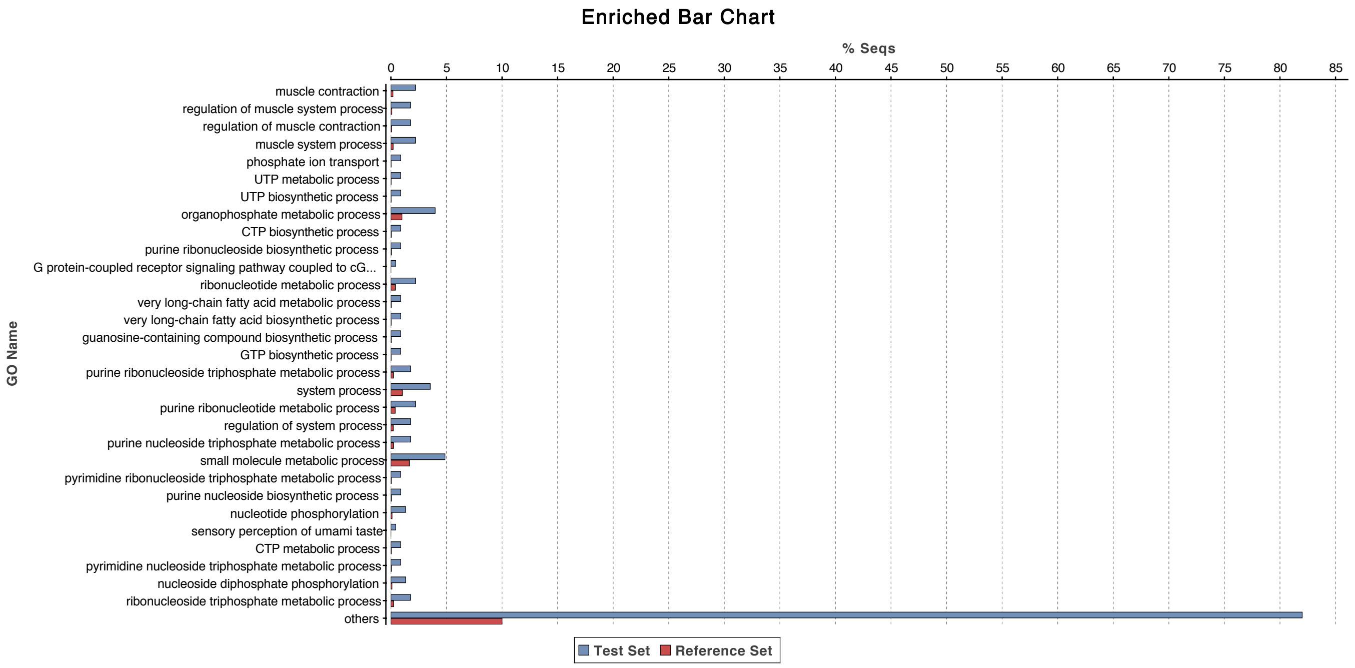

B

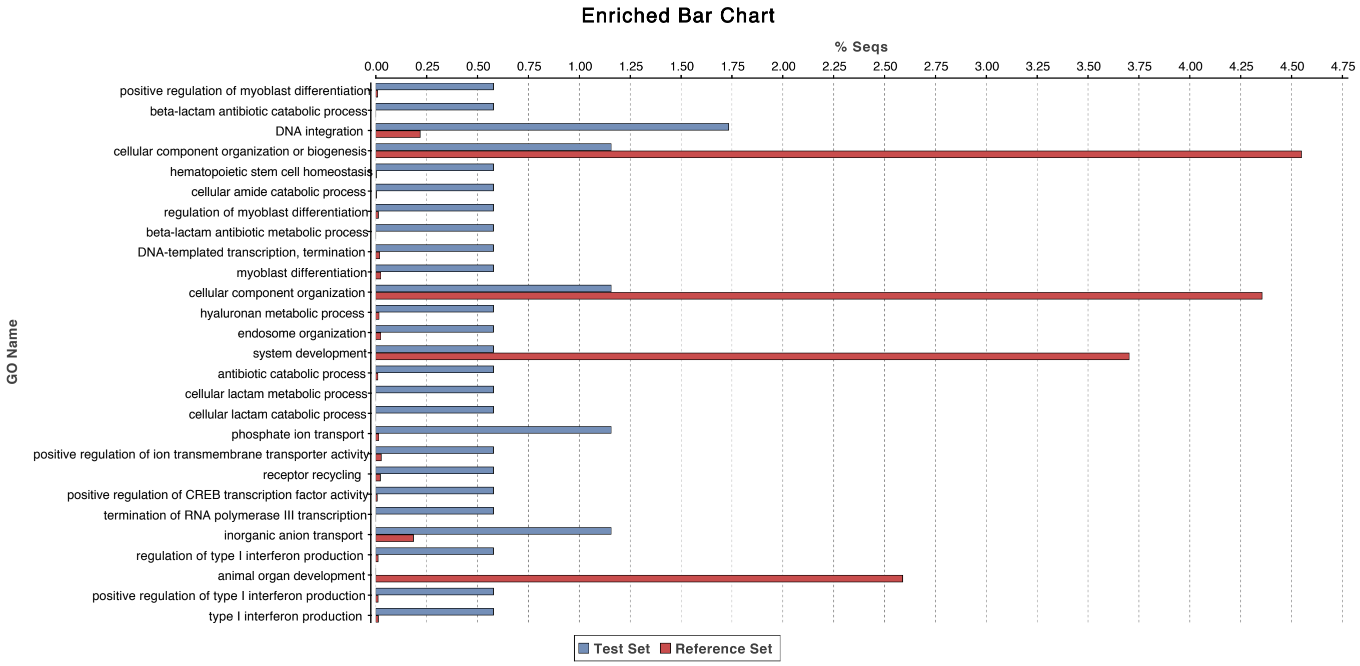

Figure S12

A

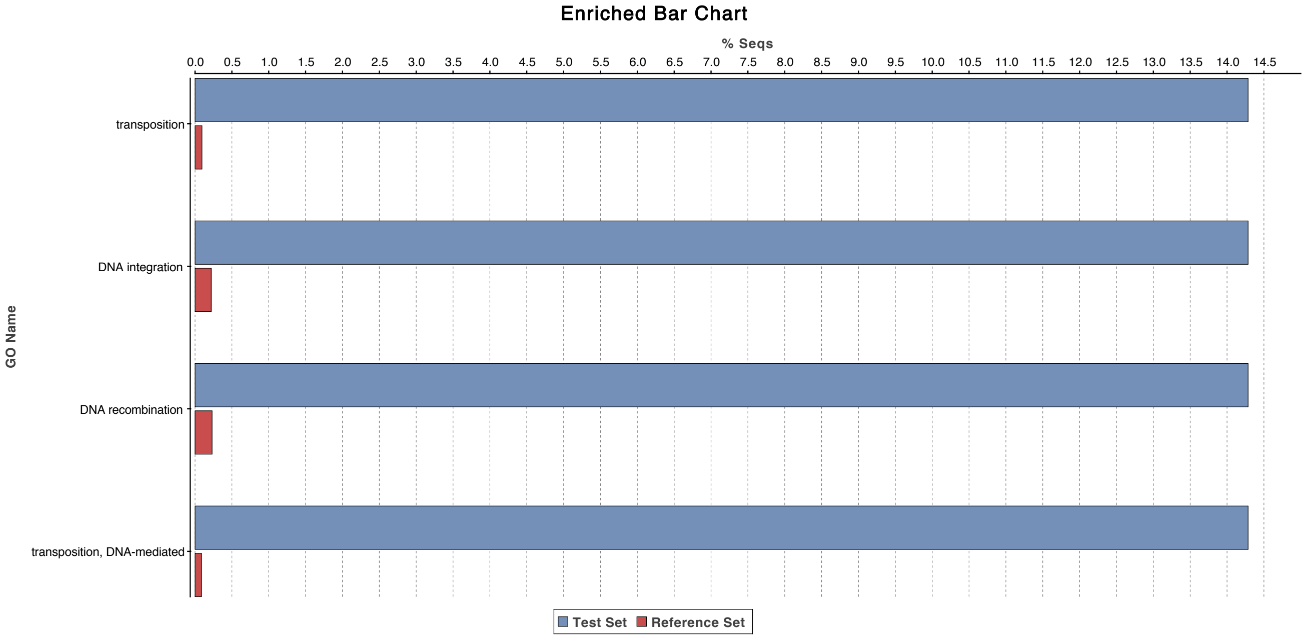

B

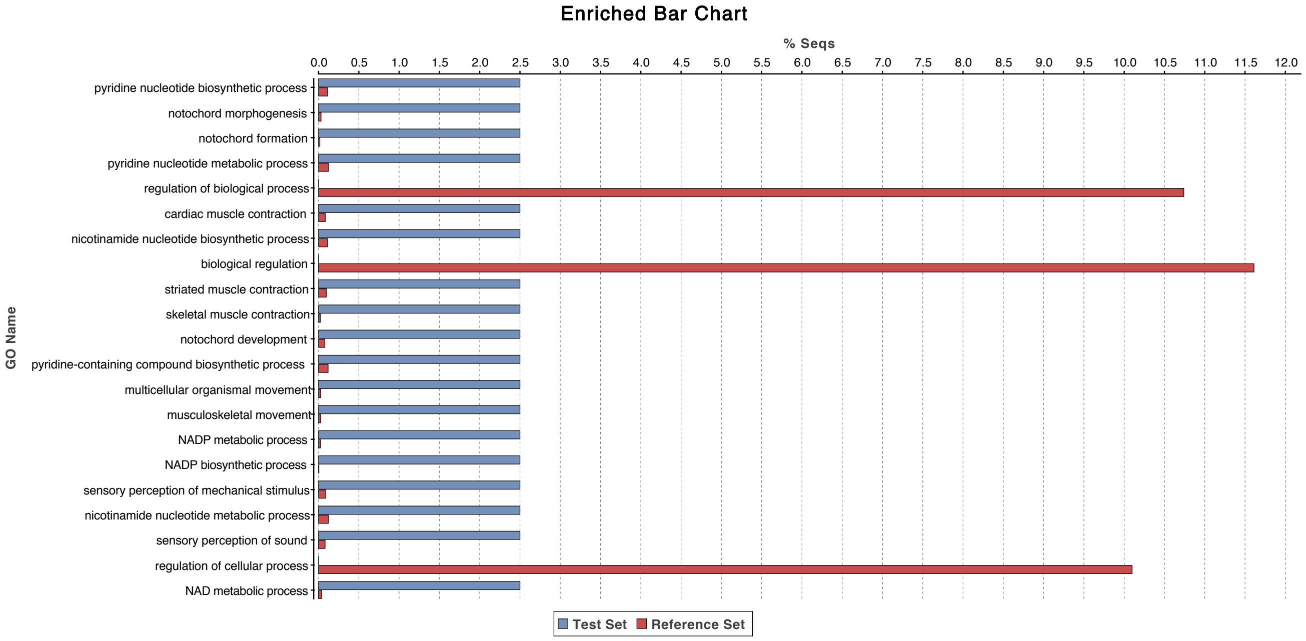

Figure S13

A

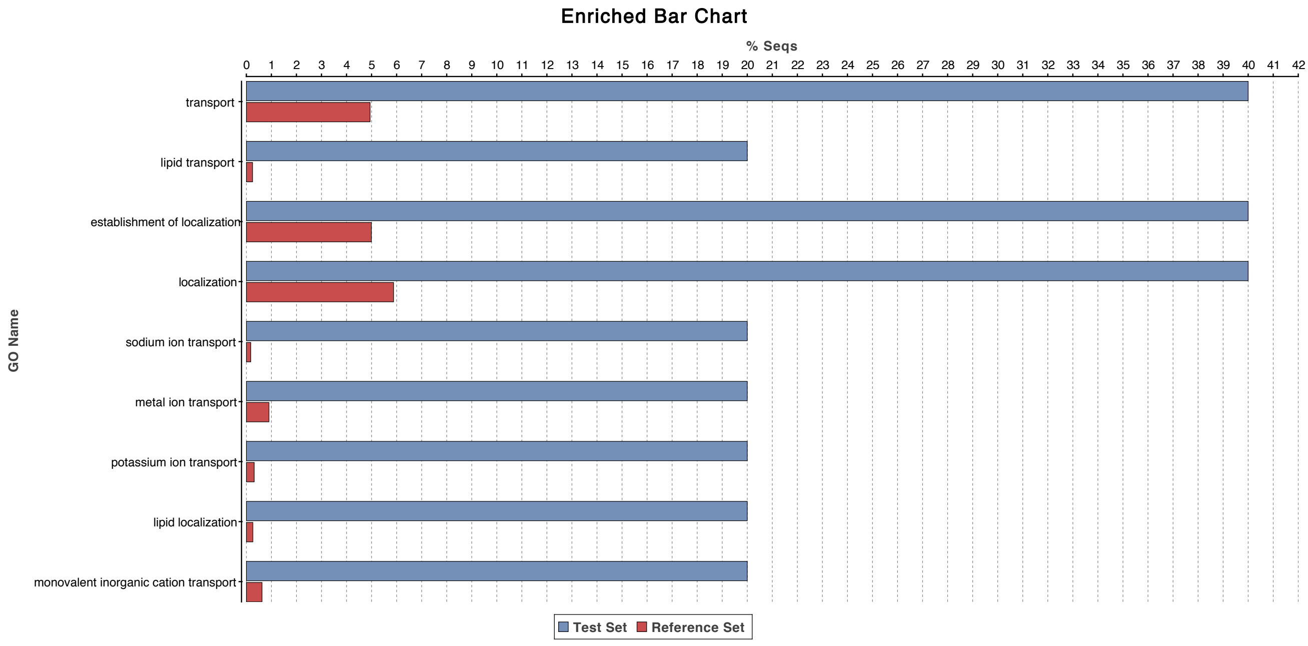

B

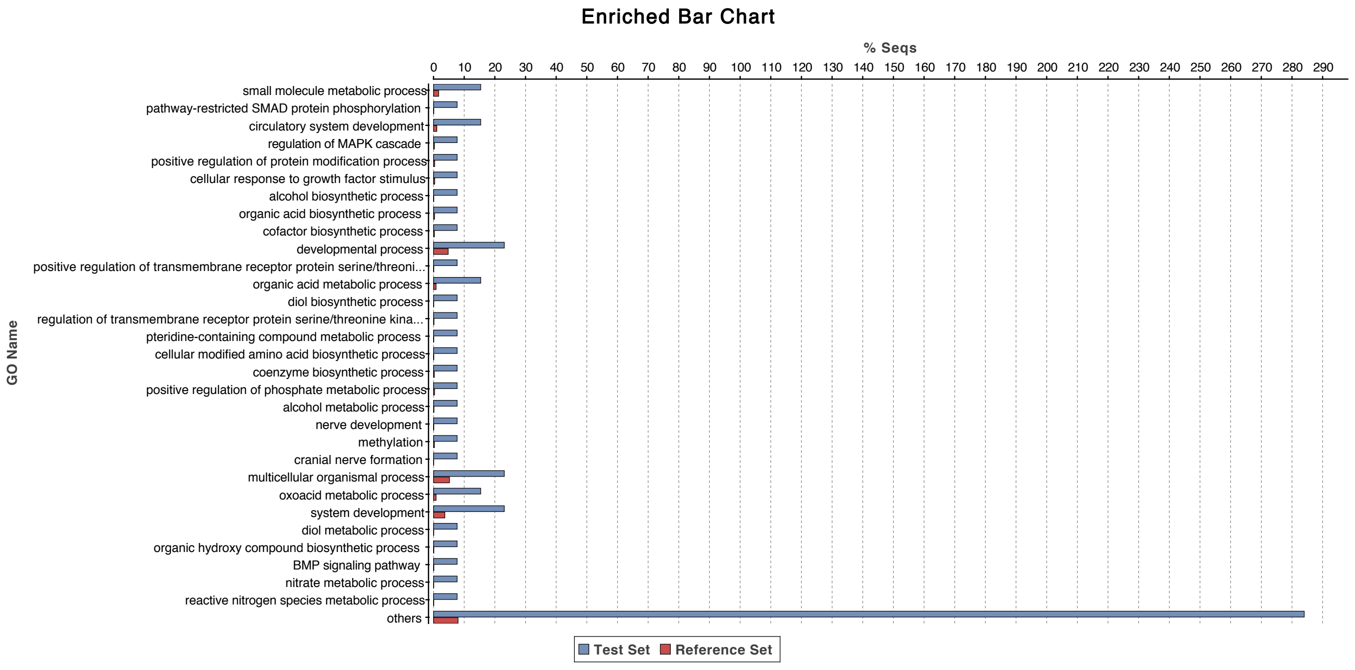

Figure S14

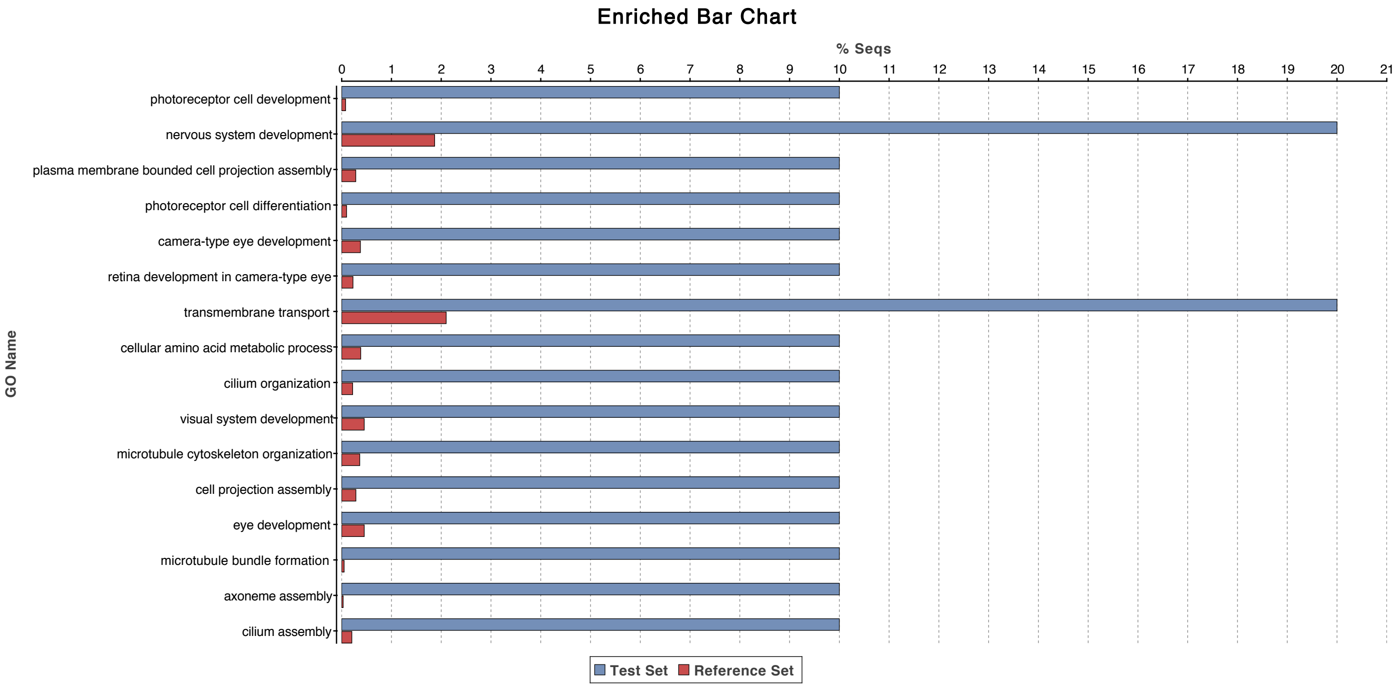

Enriched Bar Chart
% Seqs
0
1
2
3
4
5
6
7
8
9
10
11
12
13
14
15
16
17
18
19
20
21
GO Name
photoreceptor cell development
nervous system development
plasma membrane bounded cell projection assembly
photoreceptor cell differentiation
camera-type eye development
retina development in camera-type eye
transmembrane transport
cellular amino acid metabolic process
cilium organization
visual system development
microtubule cytoskeleton organization
cell projection assembly
eye development
microtubule bundle formation
axoneme assembly
cilium assembly
Test Set
Reference Set

Figure S15

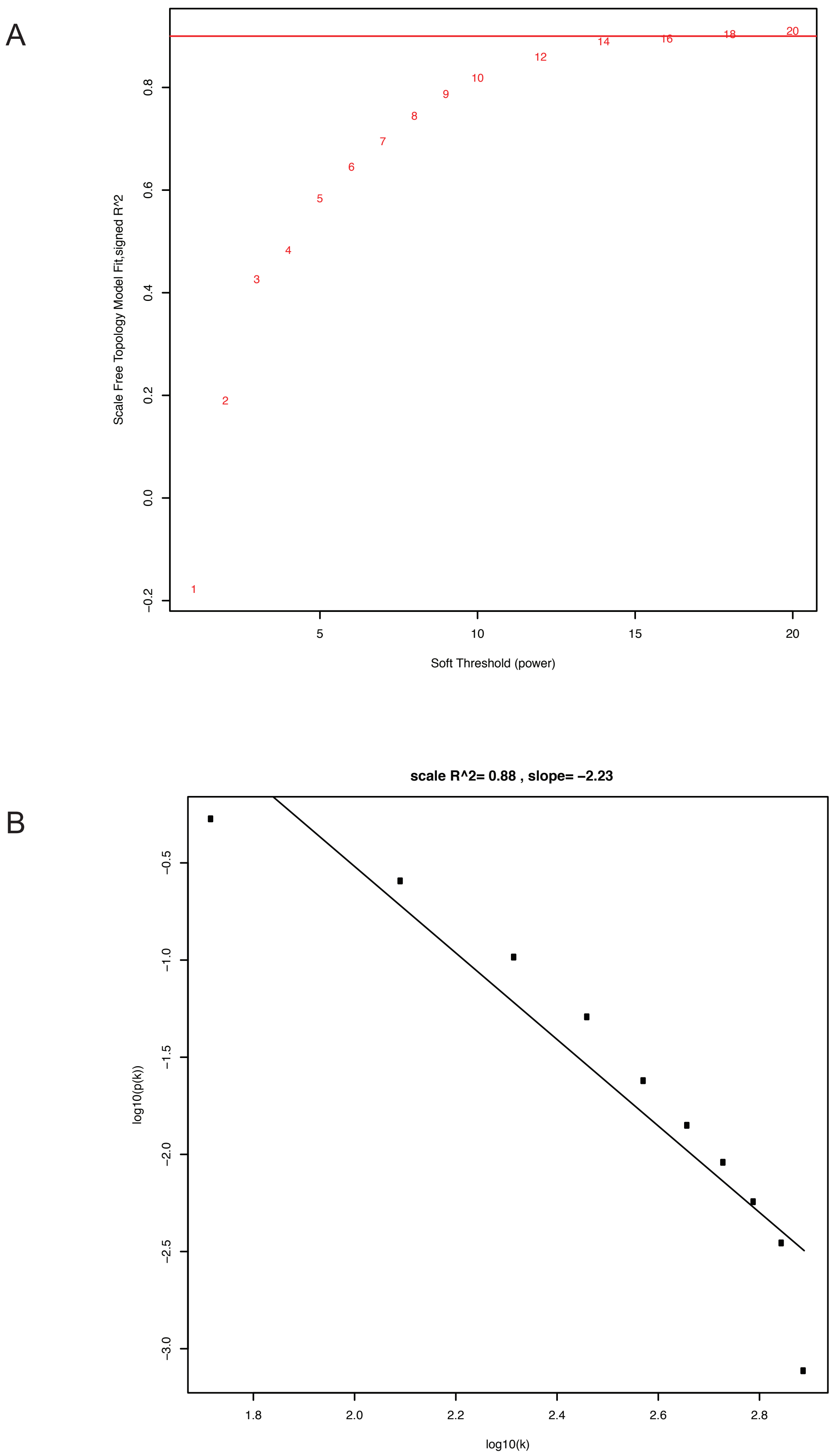
A
Scale Free Topology Model Fit,signed R^2
Soft Threshold (power)
B
scale R^2= 0.88 , slope= −2.23
log10(p(k))
log10(k)

Figure S16

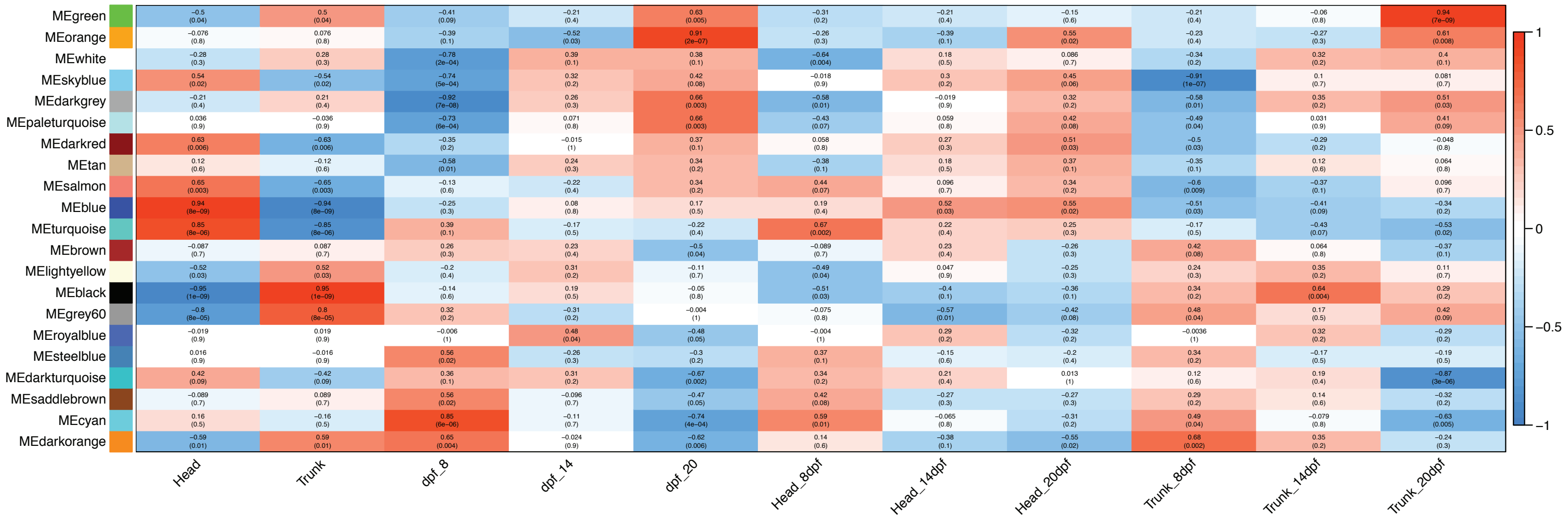
MEgreen
MEorange
MEwhite
MEskyblue
MEdarkgrey
MEpaleturquoise
MEdarkred
MEtan
MEsalmon
MEblue
MEturquoise
MEbrown
MElightyellow
MEblack
MEgrey60
MEroyalblue
MEsteelblue
MEdarkturquoise
MEsaddlebrown
MEcyan
MEdarkorange
Head
Trunk
dpf_8
dpf_14
dpf_20
Head_8dpf
Head_14dpf
Head_20dpf
Trunk_8dpf
Trunk_14dpf
Trunk_20dpf
1
0.5
0
−0.5
−1

Figure S17

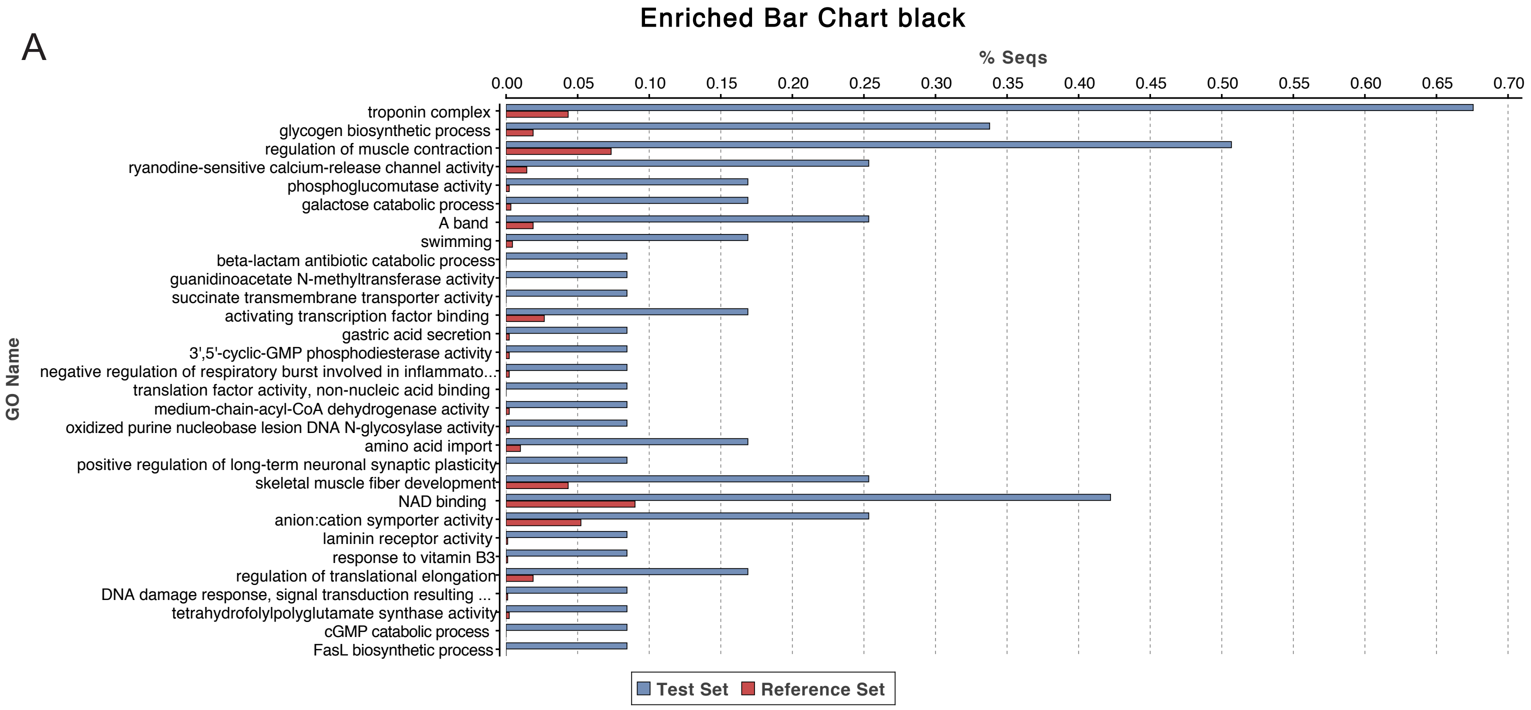
A
Enriched Bar Chart black
% Seqs
0.00 0.05 0.10 0.15 0.20 0.25 0.30 0.35 0.40 0.45 0.50 0.55 0.60 0.65 0.70
GO Name
troponin complex
glycogen biosynthetic process
regulation of muscle contraction
ryanodine-sensitive calcium-release channel activity
phosphoglucomutase activity
galactose catabolic process
A band
swimming
beta-lactam antibiotic catabolic process
guanidinoacetate N-methyltransferase activity
succinate transmembrane transporter activity
activating transcription factor binding
gastric acid secretion
3',5'-cyclic-GMP phosphodiesterase activity
negative regulation of respiratory burst involved in inflammato...
translation factor activity, non-nucleic acid binding
medium-chain-acyl-CoA dehydrogenase activity
oxidized purine nucleobase lesion DNA N-glycosylase activity
amino acid import
positive regulation of long-term neuronal synaptic plasticity
skeletal muscle fiber development
NAD binding
anion:cation symporter activity
laminin receptor activity
response to vitamin B3
regulation of translational elongation
DNA damage response, signal transduction resulting ...
tetrahydrofolylpolyglutamate synthase activity
cGMP catabolic process
FasL biosynthetic process
Test Set
Reference Set

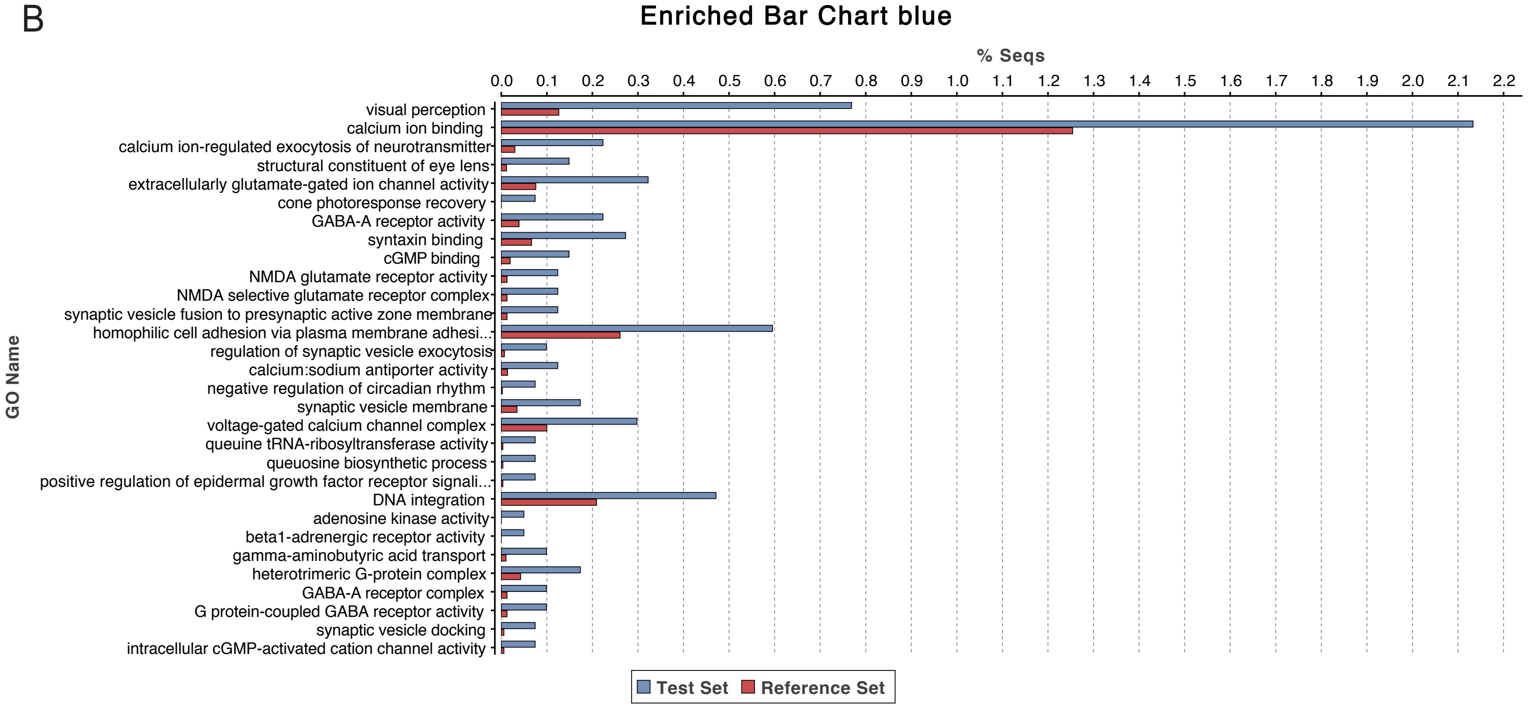
B
Enriched Bar Chart blue
% Seqs
0.0 0.1 0.2 0.3 0.4 0.5 0.6 0.7 0.8 0.9 1.0 1.1 1.2 1.3 1.4 1.5 1.6 1.7 1.8 1.9 2.0 2.1 2.2
GO Name
visual perception
calcium ion binding
calcium ion-regulated exocytosis of neurotransmitter
structural constituent of eye lens
extracellularly glutamate-gated ion channel activity
cone photoresponse recovery
GABA-A receptor activity
syntaxin binding
cGMP binding
NMDA glutamate receptor activity
NMDA selective glutamate receptor complex
synaptic vesicle fusion to presynaptic active zone membrane
homophilic cell adhesion via plasma membrane adhesi...
regulation of synaptic vesicle exocytosis
calcium:sodium antiporter activity
negative regulation of circadian rhythm
synaptic vesicle membrane
voltage-gated calcium channel complex
queuine tRNA-ribosyltransferase activity
queuosine biosynthetic process
positive regulation of epidermal growth factor receptor signali...
DNA integration
adenosine kinase activity
beta1-adrenergic receptor activity
gamma-aminobutyric acid transport
heterotrimeric G-protein complex
GABA-A receptor complex
G protein-coupled GABA receptor activity
synaptic vesicle docking
intracellular cGMP-activated cation channel activity
Test Set
Reference Set

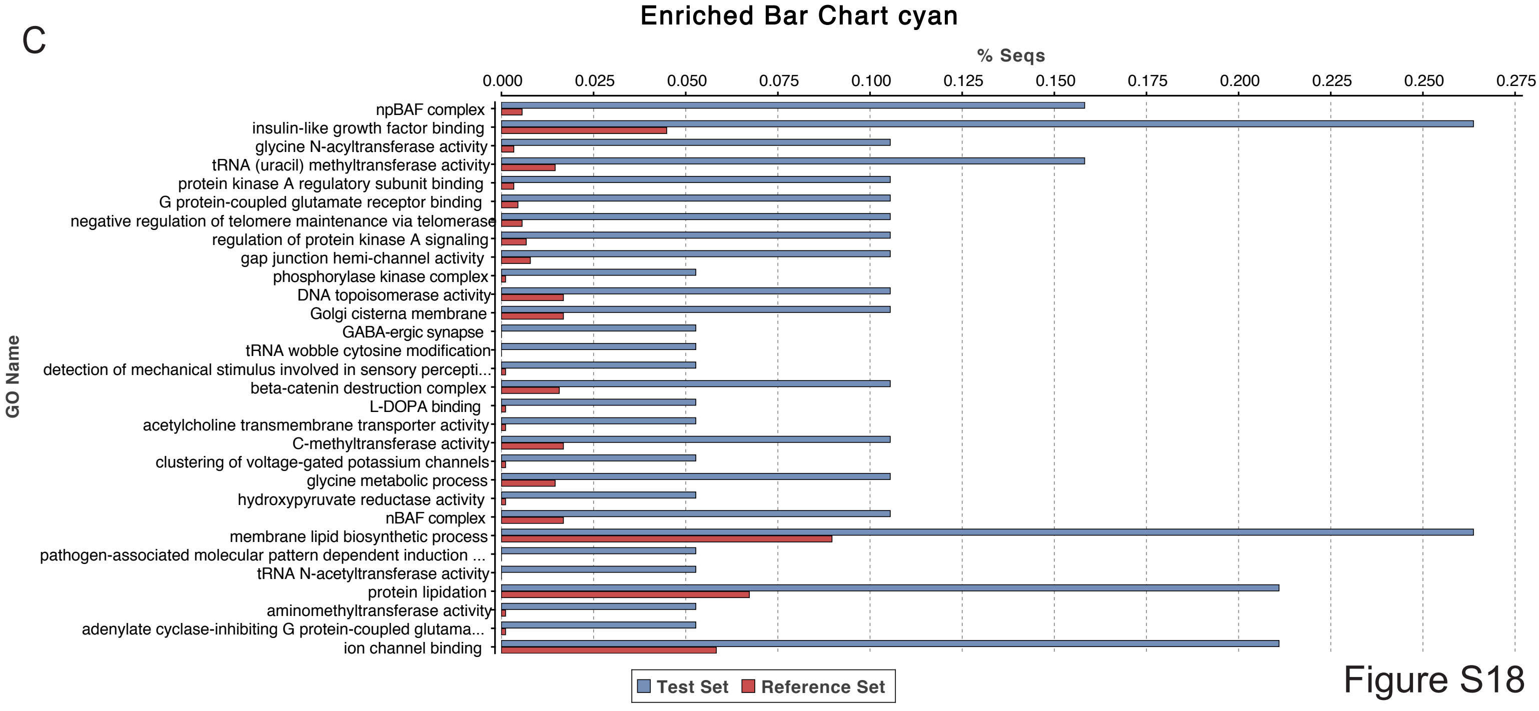
C
Enriched Bar Chart cyan
% Seqs
0.000 0.025 0.050 0.075 0.100 0.125 0.150 0.175 0.200 0.225 0.250 0.275
GO Name
npBAF complex
insulin-like growth factor binding
glycine N-acyltransferase activity
tRNA (uracil) methyltransferase activity
protein kinase A regulatory subunit binding
G protein-coupled glutamate receptor binding
negative regulation of telomere maintenance via telomerase
regulation of protein kinase A signaling
gap junction hemi-channel activity
phosphorylase kinase complex
DNA topoisomerase activity
Golgi cisterna membrane
GABA-ergic synapse
tRNA wobble cytosine modification
detection of mechanical stimulus involved in sensory percepti...
beta-catenin destruction complex
L-DOPA binding
acetylcholine transmembrane transporter activity
C-methyltransferase activity
clustering of voltage-gated potassium channels
glycine metabolic process
hydroxypyruvate reductase activity
nBAF complex
membrane lipid biosynthetic process
pathogen-associated molecular pattern dependent induction ...
tRNA N-acetyltransferase activity
protein lipidation
aminomethyltransferase activity
adenylate cyclase-inhibiting G protein-coupled glutama...
ion channel binding
Test Set
Reference Set

Figure S18

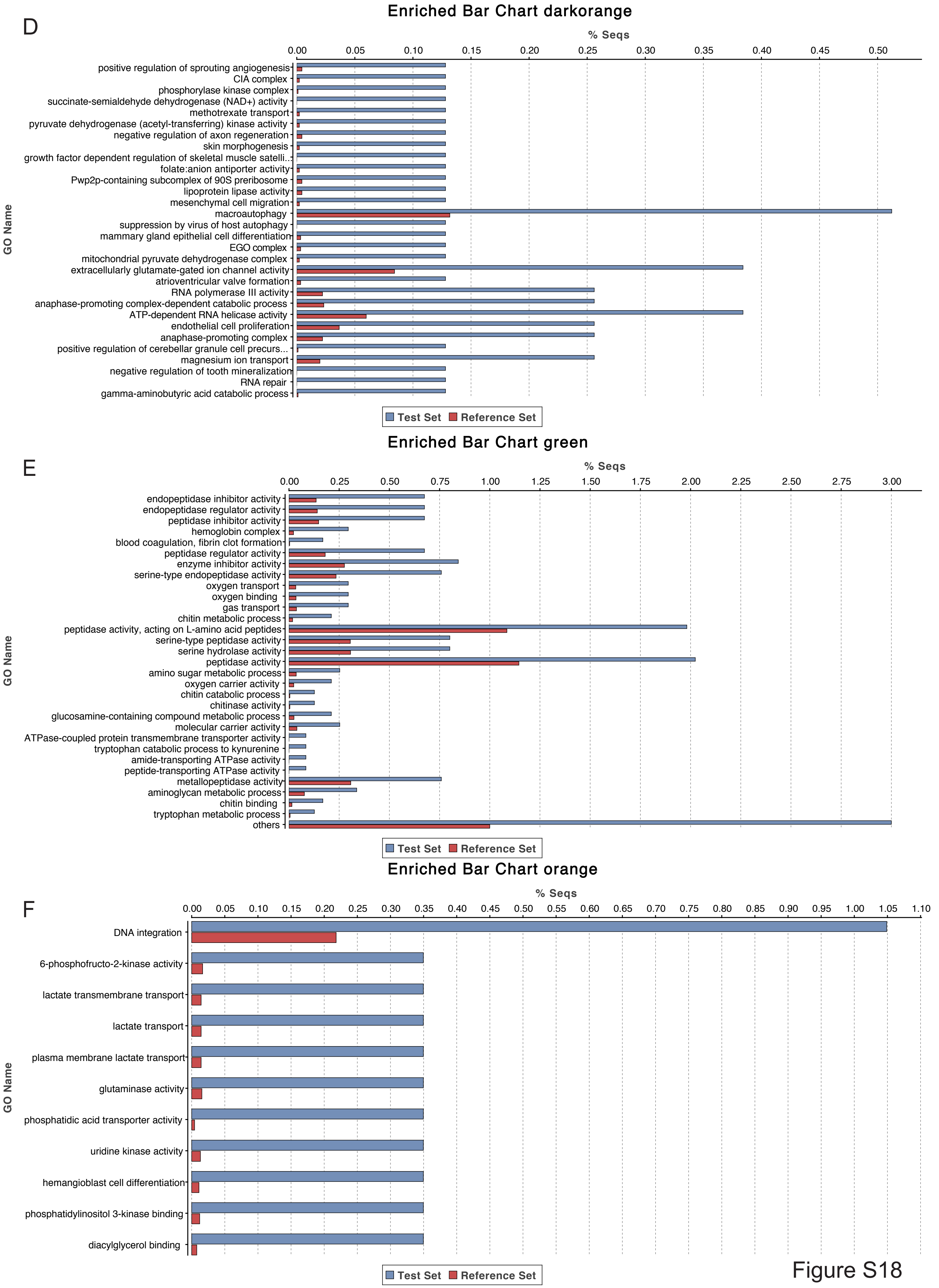
Enriched Bar Chart darkorange
D
% Seqs
0.00
0.05
0.10
0.15
0.20
0.25
0.30
0.35
0.40
0.45
0.50
GO Name
positive regulation of sprouting angiogenesis
CIA complex
phosphorylase kinase complex
succinate-semialdehyde dehydrogenase (NAD+) activity
methotrexate transport
pyruvate dehydrogenase (acetyl-transferring) kinase activity
negative regulation of axon regeneration
skin morphogenesis
growth factor dependent regulation of skeletal muscle satelli...
folate:anion antiporter activity
Pwp2p-containing subcomplex of 90S preribosome
lipoprotein lipase activity
mesenchymal cell migration
macroautophagy
suppression by virus of host autophagy
mammary gland epithelial cell differentiation
EGO complex
mitochondrial pyruvate dehydrogenase complex
extracellularly glutamate-gated ion channel activity
atrioventricular valve formation
RNA polymerase III activity
anaphase-promoting complex-dependent catabolic process
ATP-dependent RNA helicase activity
endothelial cell proliferation
anaphase-promoting complex
positive regulation of cerebellar granule cell precurs...
magnesium ion transport
negative regulation of tooth mineralization
RNA repair
gamma-aminobutyric acid catabolic process
Test Set
Reference Set
Enriched Bar Chart green
E
% Seqs
0.00
0.25
0.50
0.75
1.00
1.25
1.50
1.75
2.00
2.25
2.50
2.75
3.00
GO Name
endopeptidase inhibitor activity
endopeptidase regulator activity
peptidase inhibitor activity
hemoglobin complex
blood coagulation, fibrin clot formation
peptidase regulator activity
enzyme inhibitor activity
serine-type endopeptidase activity
oxygen transport
oxygen binding
gas transport
chitin metabolic process
peptidase activity, acting on L-amino acid peptides
serine-type peptidase activity
serine hydrolase activity
peptidase activity
amino sugar metabolic process
oxygen carrier activity
chitin catabolic process
chitinase activity
glucosamine-containing compound metabolic process
molecular carrier activity
ATPase-coupled protein transmembrane transporter activity
tryptophan catabolic process to kynurenine
amide-transporting ATPase activity
peptide-transporting ATPase activity
metallopeptidase activity
aminoglycan metabolic process
chitin binding
tryptophan metabolic process
others
Test Set
Reference Set
Enriched Bar Chart orange
F
% Seqs
0.00
0.05
0.10
0.15
0.20
0.25
0.30
0.35
0.40
0.45
0.50
0.55
0.60
0.65
0.70
0.75
0.80
0.85
0.90
0.95
1.00
1.05
1.10
GO Name
DNA integration
6-phosphofructo-2-kinase activity
lactate transmembrane transport
lactate transport
plasma membrane lactate transport
glutaminase activity
phosphatidic acid transporter activity
uridine kinase activity
hemangioblast cell differentiation
phosphatidylinositol 3-kinase binding
diacylglycerol binding
Test Set
Reference Set

Figure S18

G

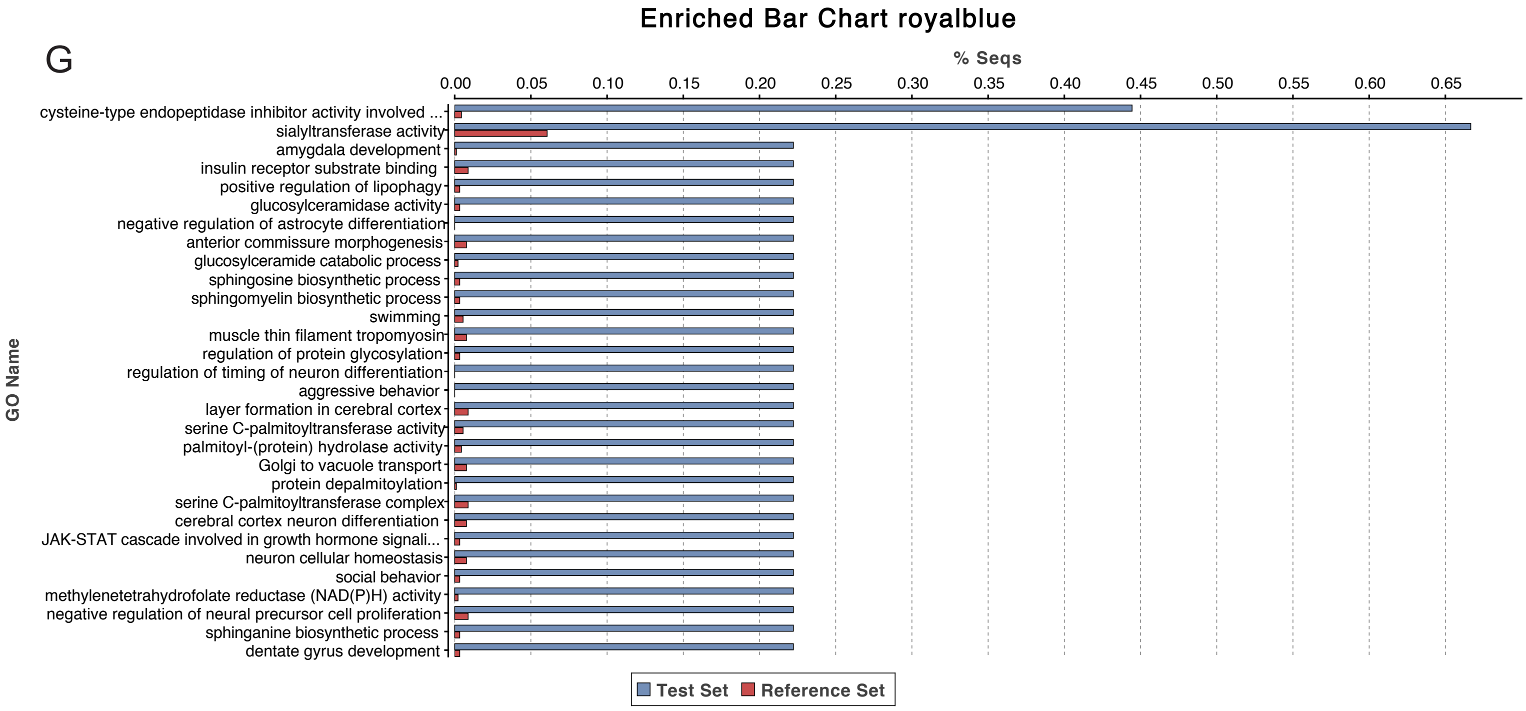

H

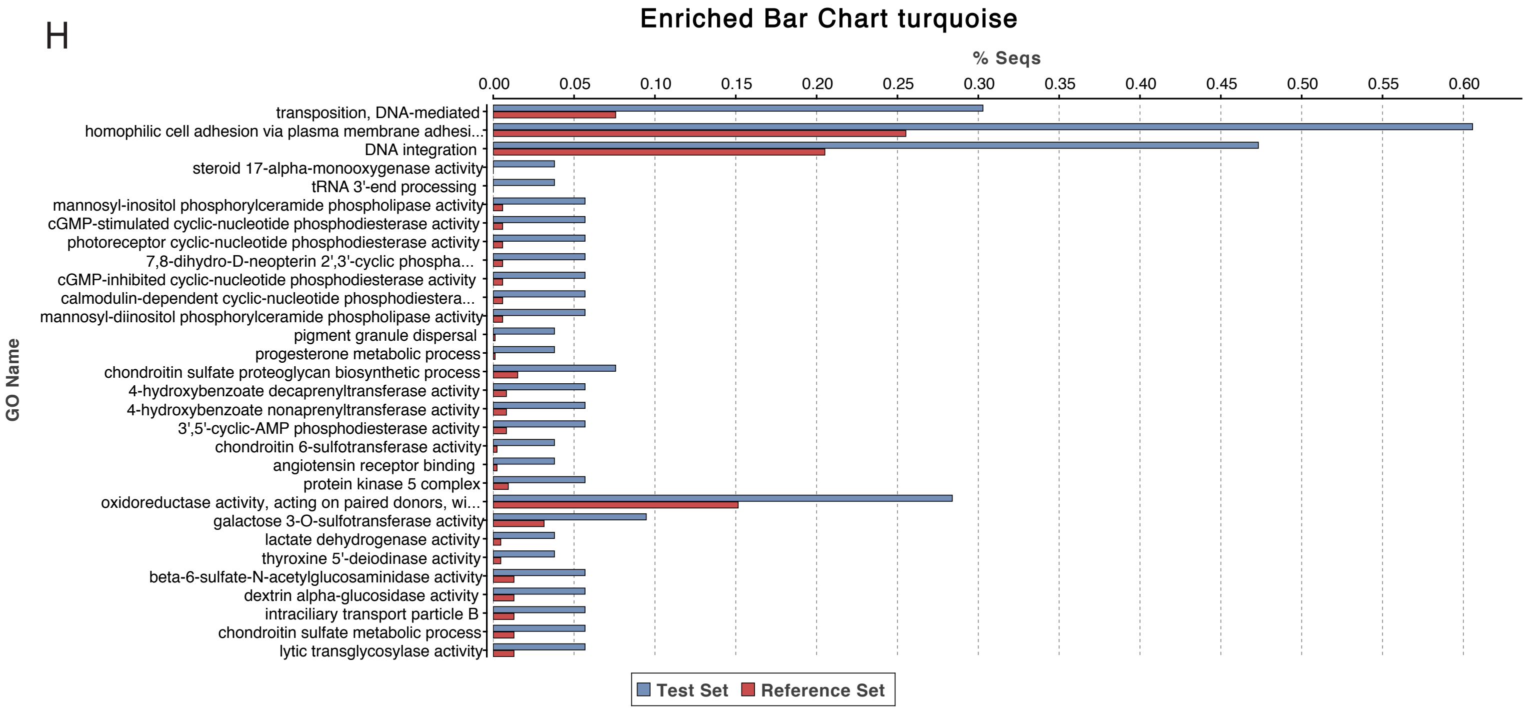

Figure S18

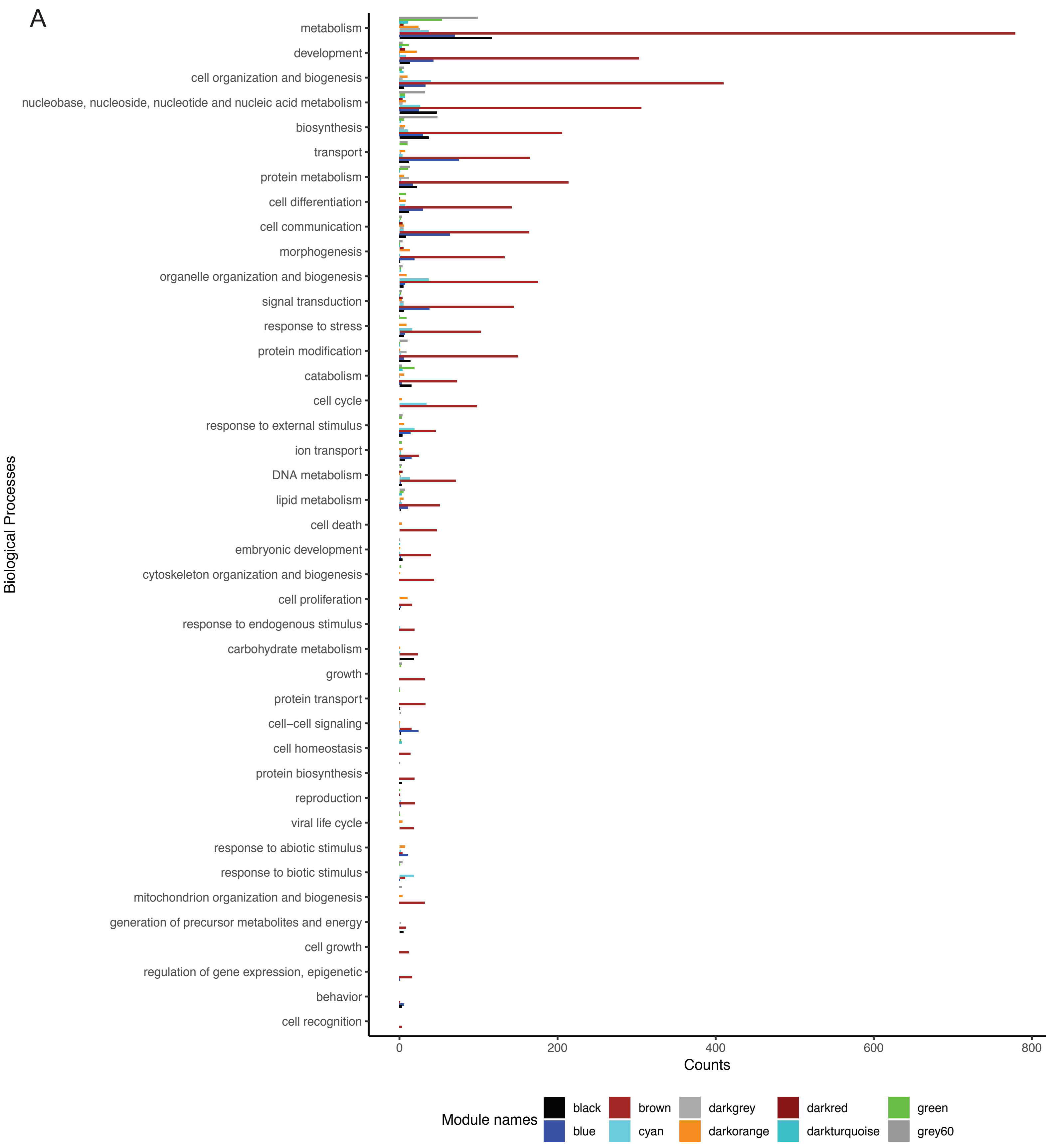
A
metabolism
development
cell organization and biogenesis
nucleobase, nucleoside, nucleotide and nucleic acid metabolism
biosynthesis
transport
protein metabolism
cell differentiation
cell communication
morphogenesis
organelle organization and biogenesis
signal transduction
response to stress
protein modification
catabolism
cell cycle
response to external stimulus
ion transport
DNA metabolism
lipid metabolism
cell death
embryonic development
cytoskeleton organization and biogenesis
cell proliferation
response to endogenous stimulus
carbohydrate metabolism
growth
protein transport
cell−cell signaling
cell homeostasis
protein biosynthesis
reproduction
viral life cycle
response to abiotic stimulus
response to biotic stimulus
mitochondrion organization and biogenesis
generation of precursor metabolites and energy
cell growth
regulation of gene expression, epigenetic
behavior
cell recognition
Biological Processes
0
200
400
600
800
Counts
Module names
black
blue
brown
cyan
darkgrey
darkorange
darkred
darkturquoise
green
grey60

Figure S19

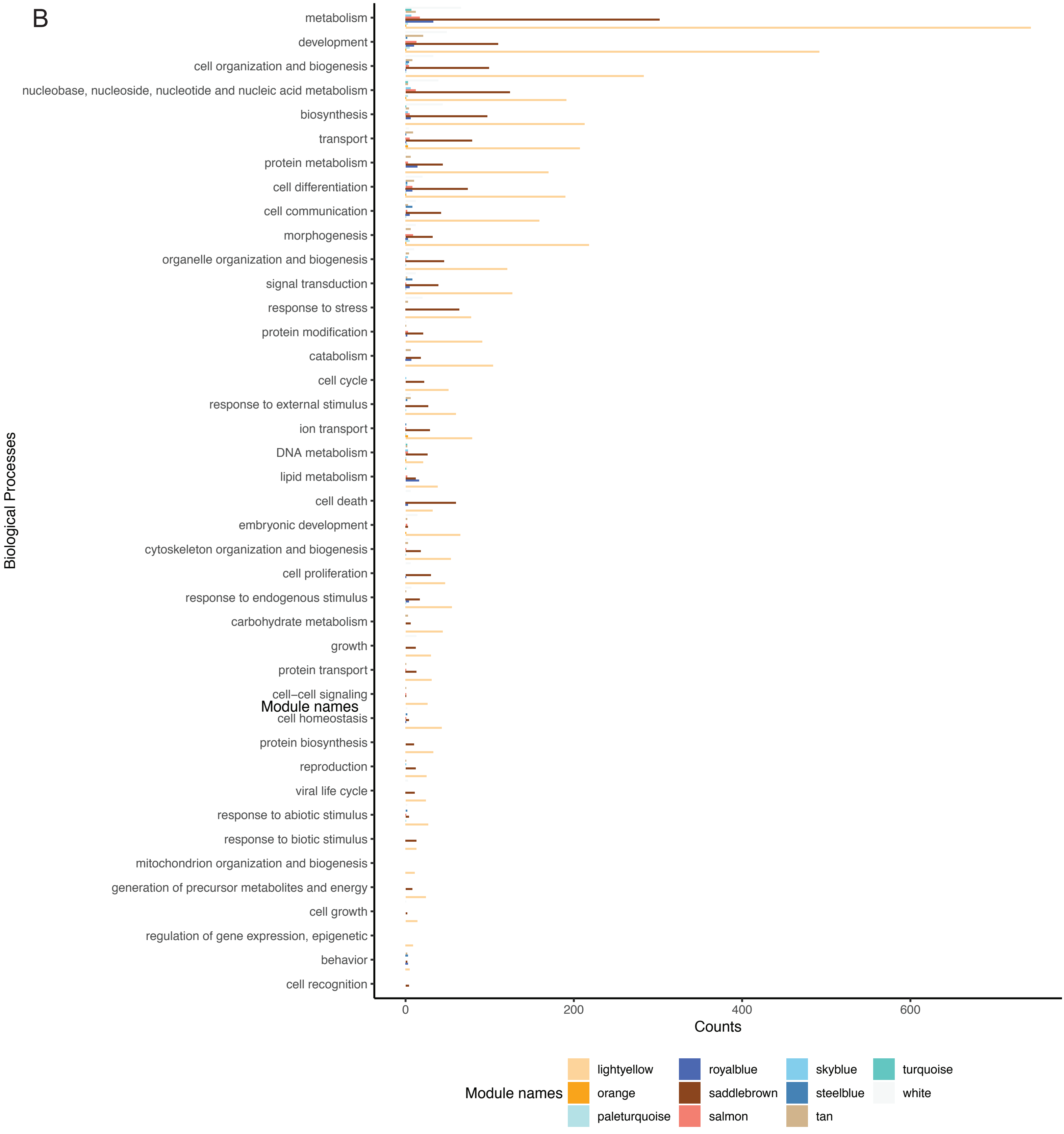
B
Biological Processes
metabolism
development
cell organization and biogenesis
nucleobase, nucleoside, nucleotide and nucleic acid metabolism
biosynthesis
transport
protein metabolism
cell differentiation
cell communication
morphogenesis
organelle organization and biogenesis
signal transduction
response to stress
protein modification
catabolism
cell cycle
response to external stimulus
ion transport
DNA metabolism
lipid metabolism
cell death
embryonic development
cytoskeleton organization and biogenesis
cell proliferation
response to endogenous stimulus
carbohydrate metabolism
growth
protein transport
cell−cell signaling
Module names
cell homeostasis
protein biosynthesis
reproduction
viral life cycle
response to abiotic stimulus
response to biotic stimulus
mitochondrion organization and biogenesis
generation of precursor metabolites and energy
cell growth
regulation of gene expression, epigenetic
behavior
cell recognition
0
200
400
600
Counts
Module names
lightyellow
orange
paleturquoise
royalblue
saddlebrown
salmon
skyblue
steelblue
tan
turquoise
white

Figure S19

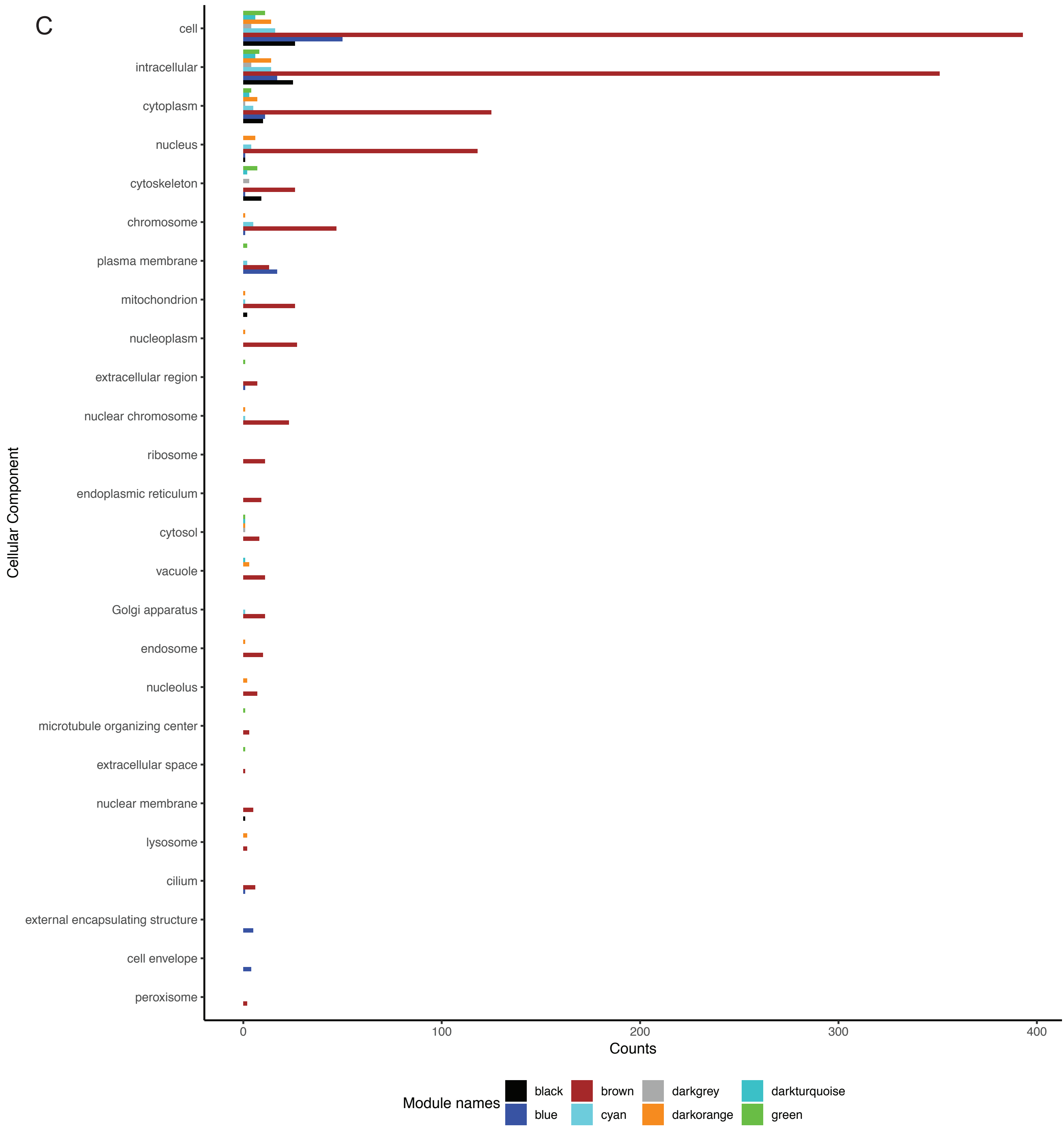
C
cell
intracellular
cytoplasm
nucleus
cytoskeleton
chromosome
plasma membrane
mitochondrion
nucleoplasm
extracellular region
nuclear chromosome
ribosome
endoplasmic reticulum
cytosol
vacuole
Golgi apparatus
endosome
nucleolus
microtubule organizing center
extracellular space
nuclear membrane
lysosome
cilium
external encapsulating structure
cell envelope
peroxisome
Cellular Component
0
100
200
300
400
Counts
Module names
black
brown
darkgrey
darkturquoise
blue
cyan
darkorange
green

Figure S19

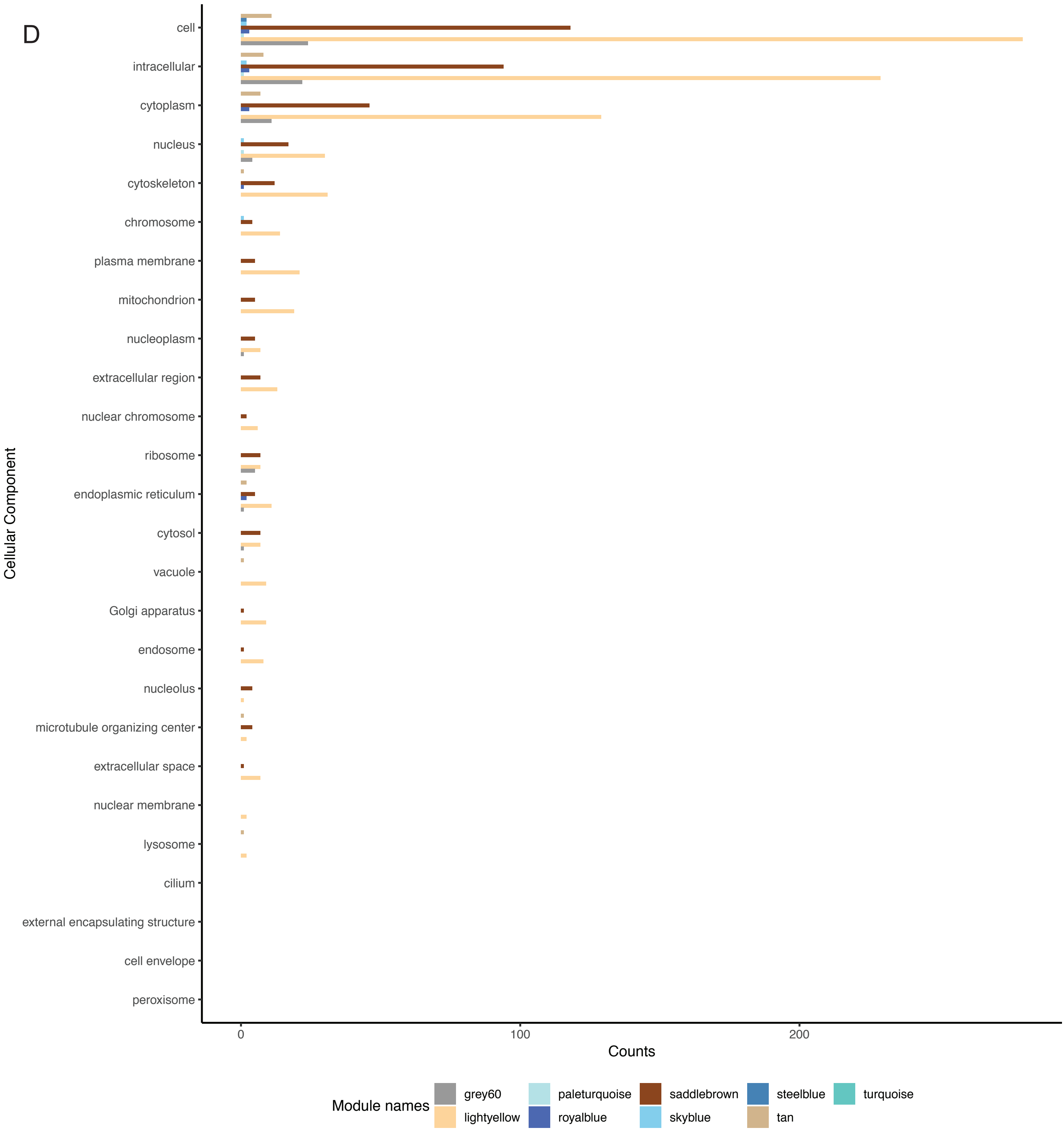
D
Cellular Component
cell
intracellular
cytoplasm
nucleus
cytoskeleton
chromosome
plasma membrane
mitochondrion
nucleoplasm
extracellular region
nuclear chromosome
ribosome
endoplasmic reticulum
cytosol
vacuole
Golgi apparatus
endosome
nucleolus
microtubule organizing center
extracellular space
nuclear membrane
lysosome
cilium
external encapsulating structure
cell envelope
peroxisome
0
100
200
Counts
Module names
grey60
lightyellow
paleturquoise
royalblue
saddlebrown
skyblue
steelblue
tan
turquoise

Figure S19

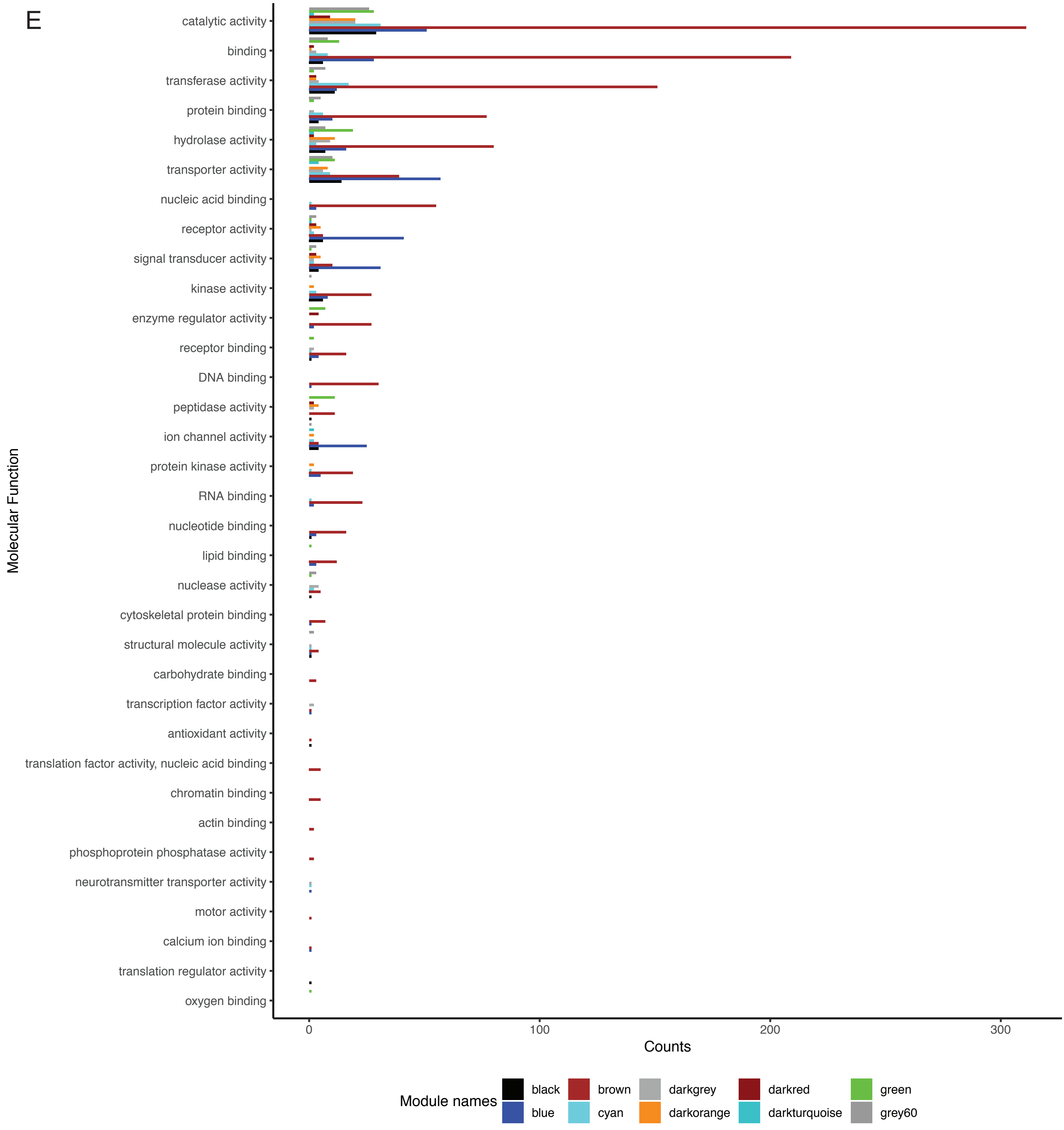
E
catalytic activity
binding
transferase activity
protein binding
hydrolase activity
transporter activity
nucleic acid binding
receptor activity
signal transducer activity
kinase activity
enzyme regulator activity
receptor binding
DNA binding
peptidase activity
ion channel activity
protein kinase activity
RNA binding
nucleotide binding
lipid binding
nuclease activity
cytoskeletal protein binding
structural molecule activity
carbohydrate binding
transcription factor activity
antioxidant activity
translation factor activity, nucleic acid binding
chromatin binding
actin binding
phosphoprotein phosphatase activity
neurotransmitter transporter activity
motor activity
calcium ion binding
translation regulator activity
oxygen binding
Molecular Function
0
100
200
300
Counts
Module names
black
blue
brown
cyan
darkgrey
darkorange
darkred
darkturquoise
green
grey60

Figure S19

F

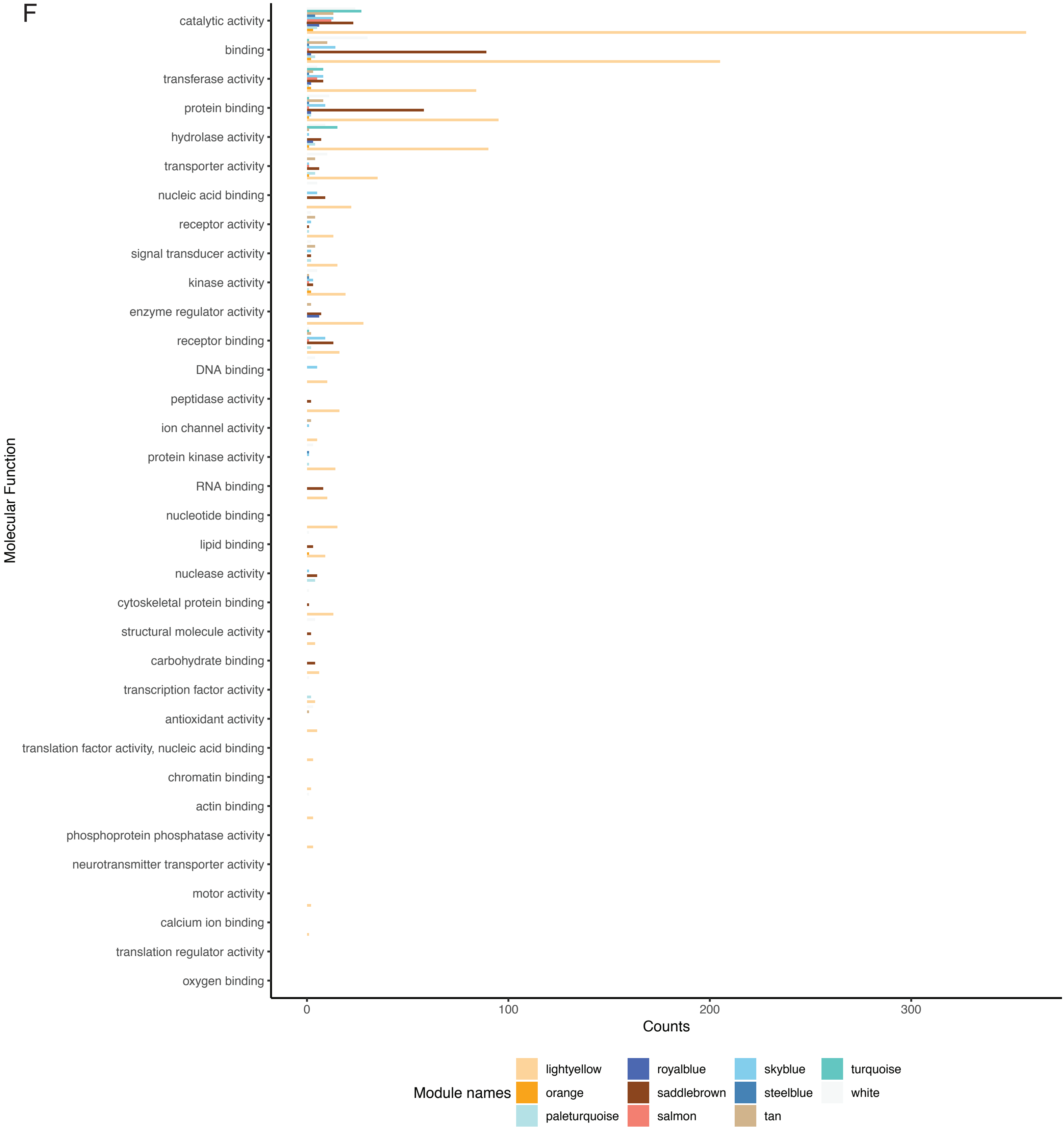
catalytic activity
binding
transferase activity
protein binding
hydrolase activity
transporter activity
nucleic acid binding
receptor activity
signal transducer activity
kinase activity
enzyme regulator activity
receptor binding
DNA binding
peptidase activity
ion channel activity
protein kinase activity
RNA binding
nucleotide binding
lipid binding
nuclease activity
cytoskeletal protein binding
structural molecule activity
carbohydrate binding
transcription factor activity
antioxidant activity
translation factor activity, nucleic acid binding
chromatin binding
actin binding
phosphoprotein phosphatase activity
neurotransmitter transporter activity
motor activity
calcium ion binding
translation regulator activity
oxygen binding
Molecular Function
0
100
200
300
Counts
Module names
lightyellow
orange
paleturquoise
royalblue
saddlebrown
salmon
skyblue
steelblue
tan
turquoise
white

Figure S19

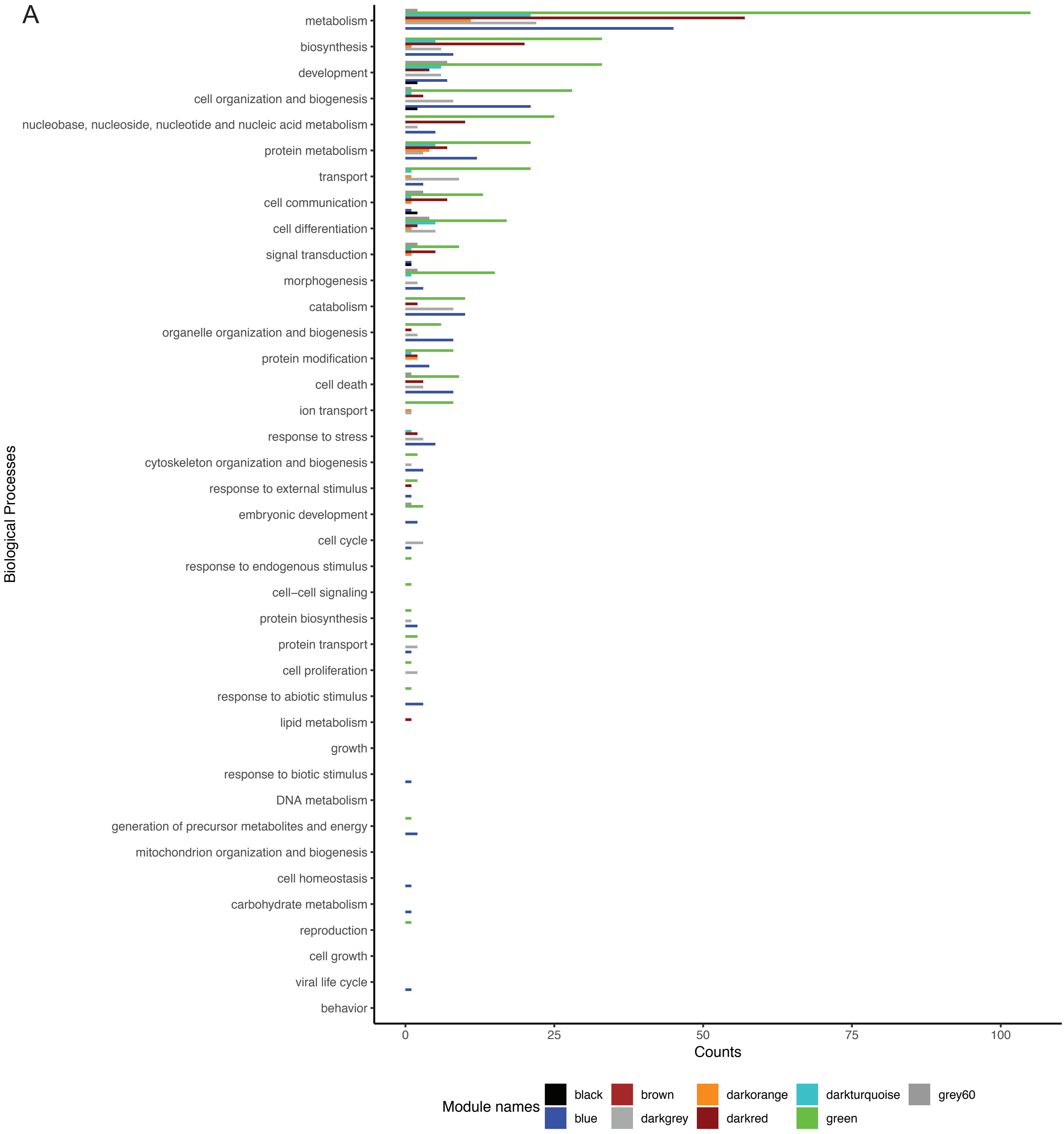
A
Biological Processes
metabolism
biosynthesis
development
cell organization and biogenesis
nucleobase, nucleoside, nucleotide and nucleic acid metabolism
protein metabolism
transport
cell communication
cell differentiation
signal transduction
morphogenesis
catabolism
organelle organization and biogenesis
protein modification
cell death
ion transport
response to stress
cytoskeleton organization and biogenesis
response to external stimulus
embryonic development
cell cycle
response to endogenous stimulus
cell–cell signaling
protein biosynthesis
protein transport
cell proliferation
response to abiotic stimulus
lipid metabolism
growth
response to biotic stimulus
DNA metabolism
generation of precursor metabolites and energy
mitochondrion organization and biogenesis
cell homeostasis
carbohydrate metabolism
reproduction
cell growth
viral life cycle
behavior
0
25
50
75
100
Counts
Module names
black
blue
brown
darkgrey
darkorange
darkred
darkturquoise
green
grey60

Figure S20

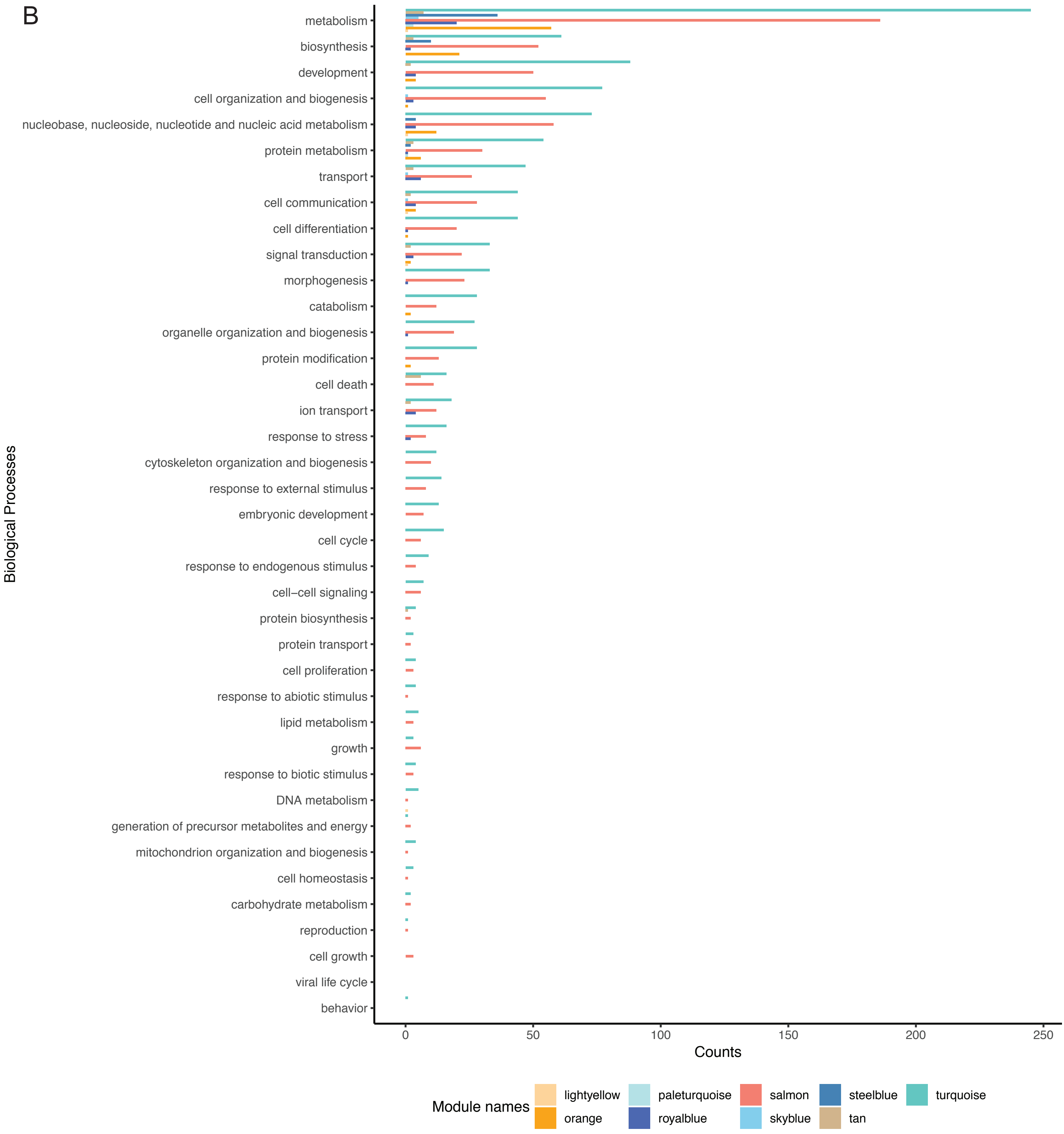
B
Biological Processes
metabolism
biosynthesis
development
cell organization and biogenesis
nucleobase, nucleoside, nucleotide and nucleic acid metabolism
protein metabolism
transport
cell communication
cell differentiation
signal transduction
morphogenesis
catabolism
organelle organization and biogenesis
protein modification
cell death
ion transport
response to stress
cytoskeleton organization and biogenesis
response to external stimulus
embryonic development
cell cycle
response to endogenous stimulus
cell–cell signaling
protein biosynthesis
protein transport
cell proliferation
response to abiotic stimulus
lipid metabolism
growth
response to biotic stimulus
DNA metabolism
generation of precursor metabolites and energy
mitochondrion organization and biogenesis
cell homeostasis
carbohydrate metabolism
reproduction
cell growth
viral life cycle
behavior
0
50
100
150
200
250
Counts
Module names
lightyellow
orange
paleturquoise
royalblue
salmon
skyblue
steelblue
tan
turquoise

Figure S20

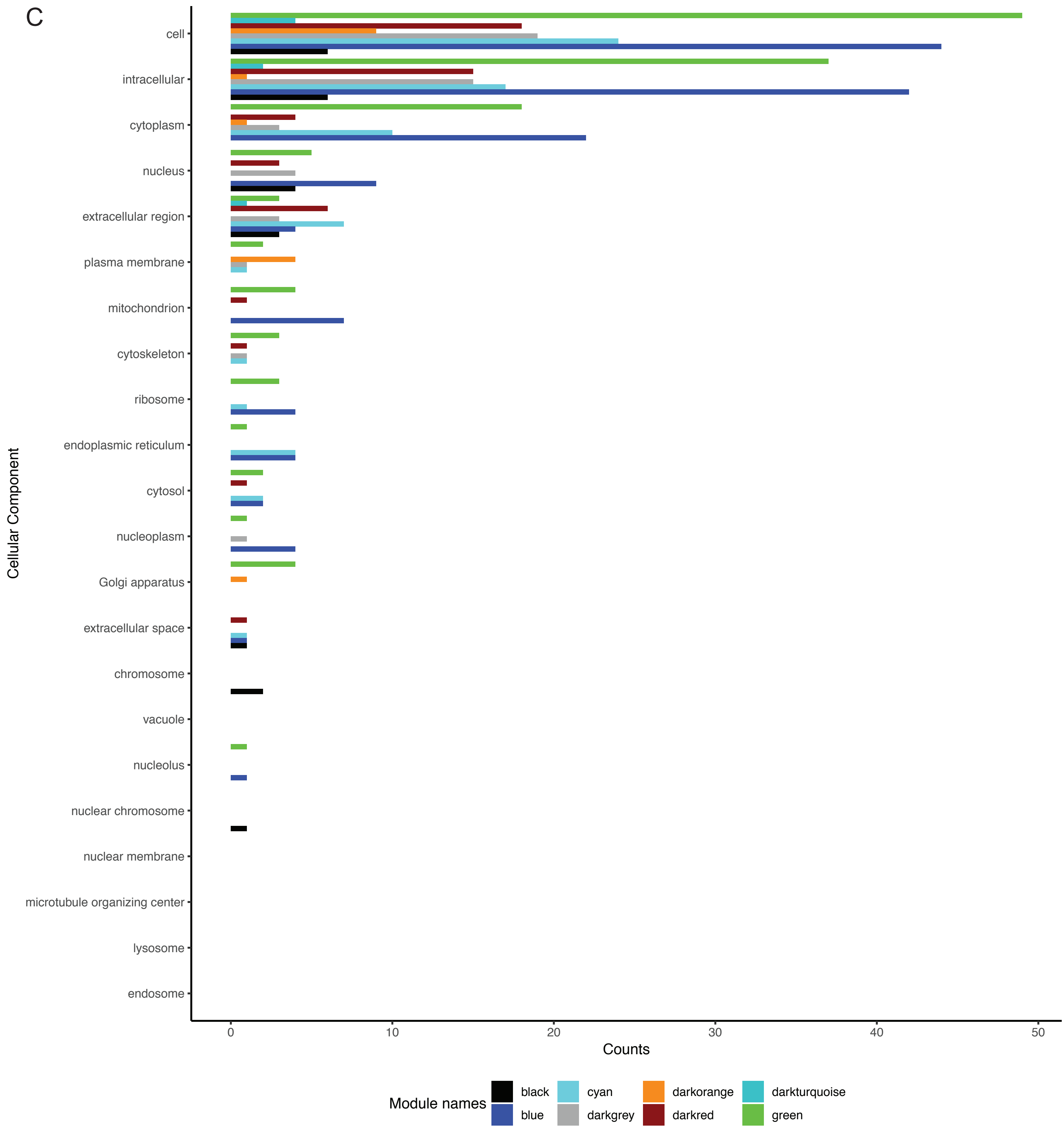
C
cell
intracellular
cytoplasm
nucleus
extracellular region
plasma membrane
mitochondrion
cytoskeleton
ribosome
endoplasmic reticulum
cytosol
nucleoplasm
Golgi apparatus
extracellular space
chromosome
vacuole
nucleolus
nuclear chromosome
nuclear membrane
microtubule organizing center
lysosome
endosome
Cellular Component
0
10
20
30
40
50
Counts
Module names
black
blue
cyan
darkgrey
darkorange
darkred
darkturquoise
green

Figure S20

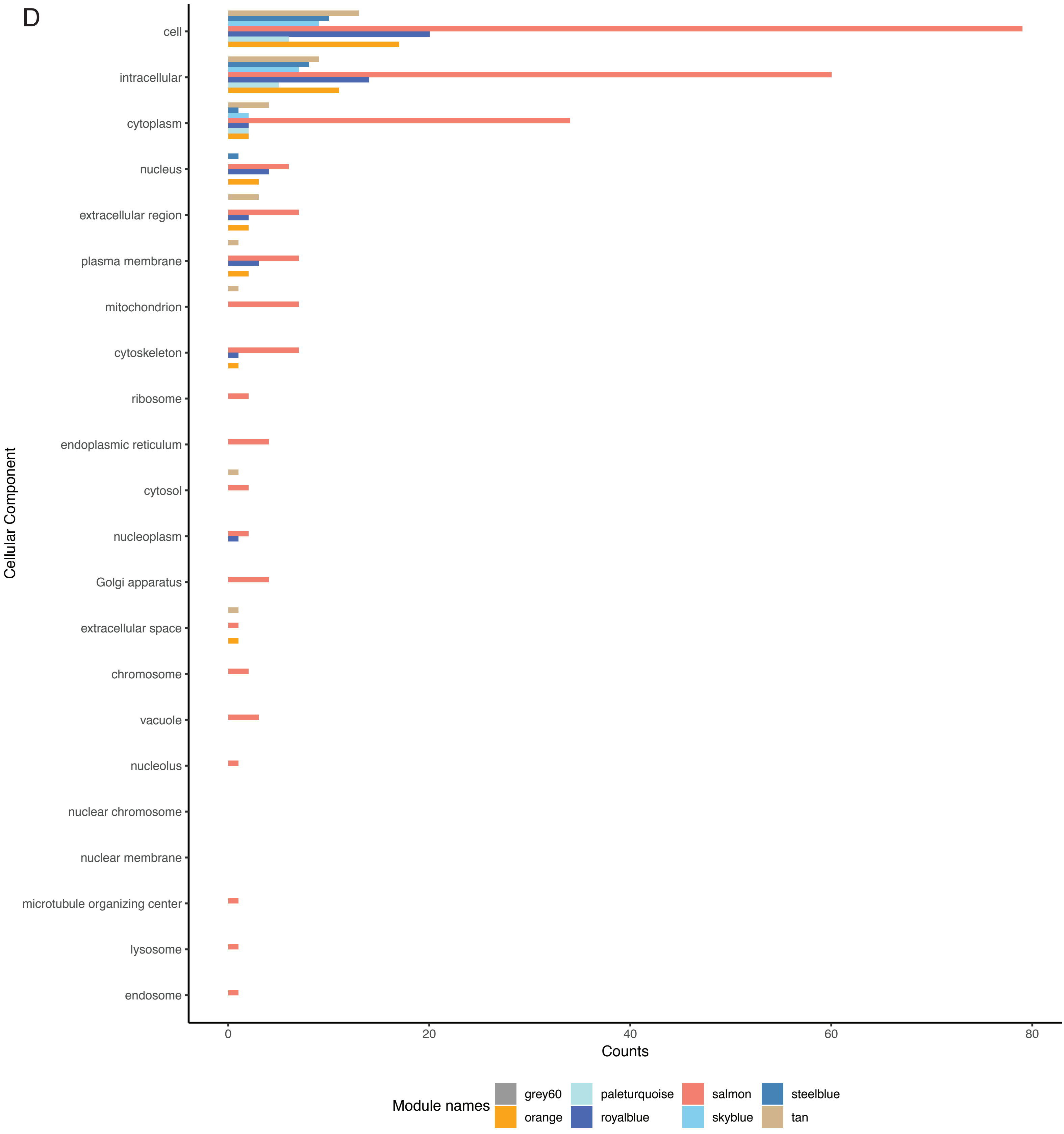
D
cell
intracellular
cytoplasm
nucleus
extracellular region
plasma membrane
mitochondrion
cytoskeleton
ribosome
endoplasmic reticulum
cytosol
nucleoplasm
Golgi apparatus
extracellular space
chromosome
vacuole
nucleolus
nuclear chromosome
nuclear membrane
microtubule organizing center
lysosome
endosome
Cellular Component
0
20
40
60
80
Counts
Module names
grey60
orange
paleturquoise
royalblue
salmon
skyblue
steelblue
tan

Figure S20

E

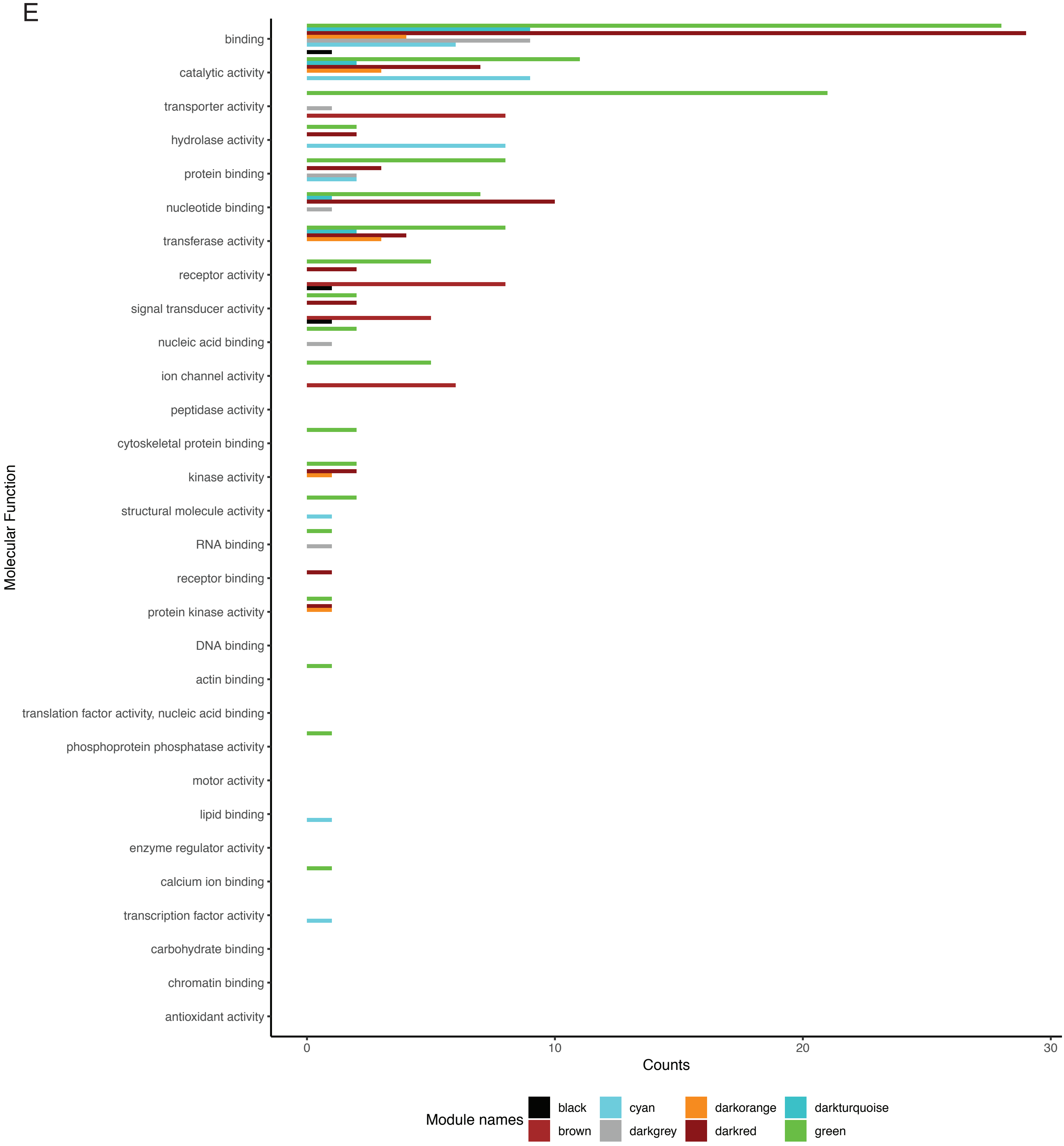
binding
catalytic activity
transporter activity
hydrolase activity
protein binding
nucleotide binding
transferase activity
receptor activity
signal transducer activity
nucleic acid binding
ion channel activity
peptidase activity
cytoskeletal protein binding
kinase activity
structural molecule activity
RNA binding
receptor binding
protein kinase activity
DNA binding
actin binding
translation factor activity, nucleic acid binding
phosphoprotein phosphatase activity
motor activity
lipid binding
enzyme regulator activity
calcium ion binding
transcription factor activity
carbohydrate binding
chromatin binding
antioxidant activity
Molecular Function
0
10
20
30
Counts
Module names
black
brown
cyan
darkgrey
darkorange
darkred
darkturquoise
green

Figure S20

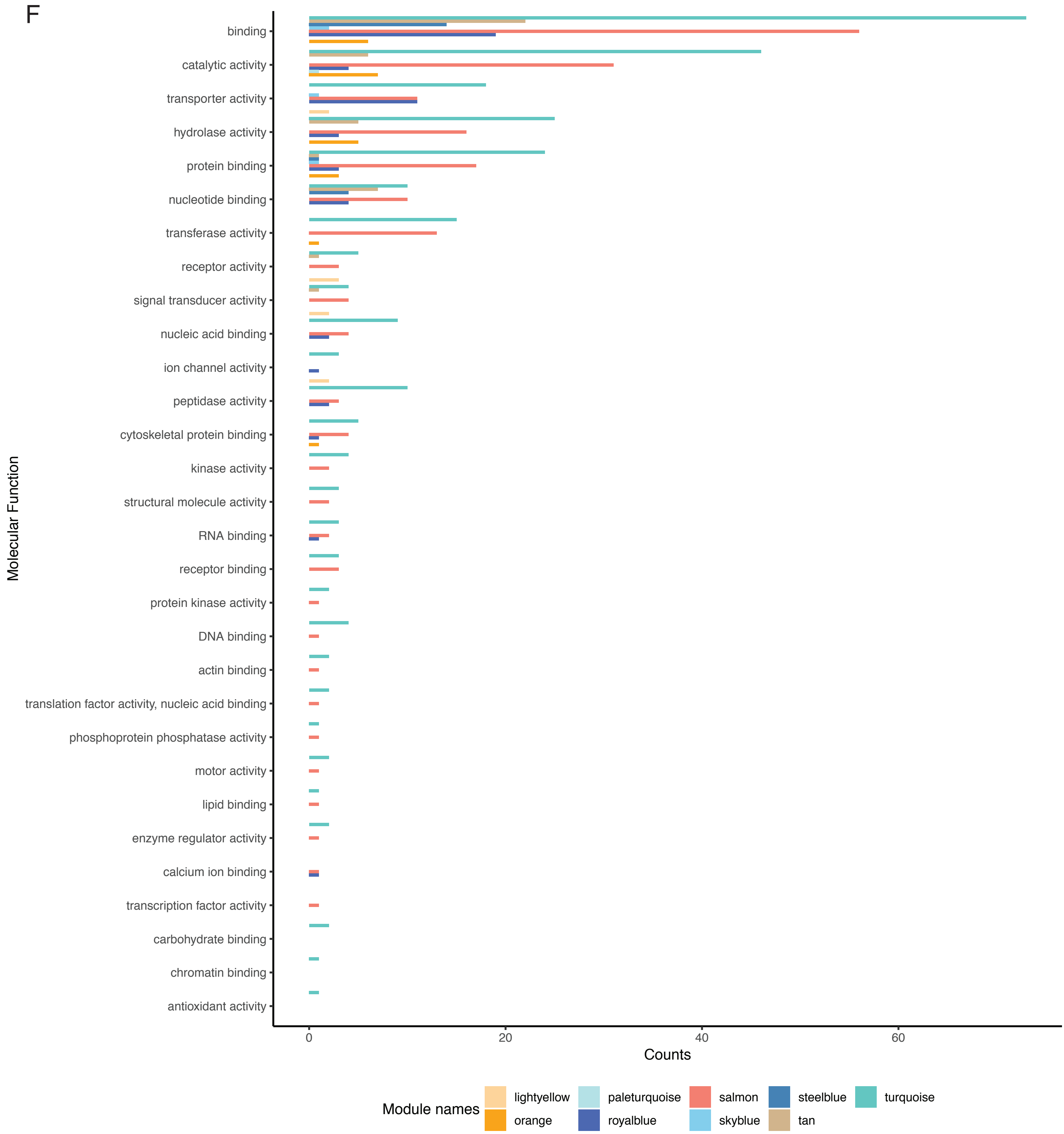
F
binding
catalytic activity
transporter activity
hydrolase activity
protein binding
nucleotide binding
transferase activity
receptor activity
signal transducer activity
nucleic acid binding
ion channel activity
peptidase activity
cytoskeletal protein binding
kinase activity
structural molecule activity
RNA binding
receptor binding
protein kinase activity
DNA binding
actin binding
translation factor activity, nucleic acid binding
phosphoprotein phosphatase activity
motor activity
lipid binding
enzyme regulator activity
calcium ion binding
transcription factor activity
carbohydrate binding
chromatin binding
antioxidant activity
Molecular Function
0
20
40
60
Counts
Module names
lightyellow
orange
paleturquoise
royalblue
salmon
skyblue
steelblue
tan
turquoise

Figure S20

A

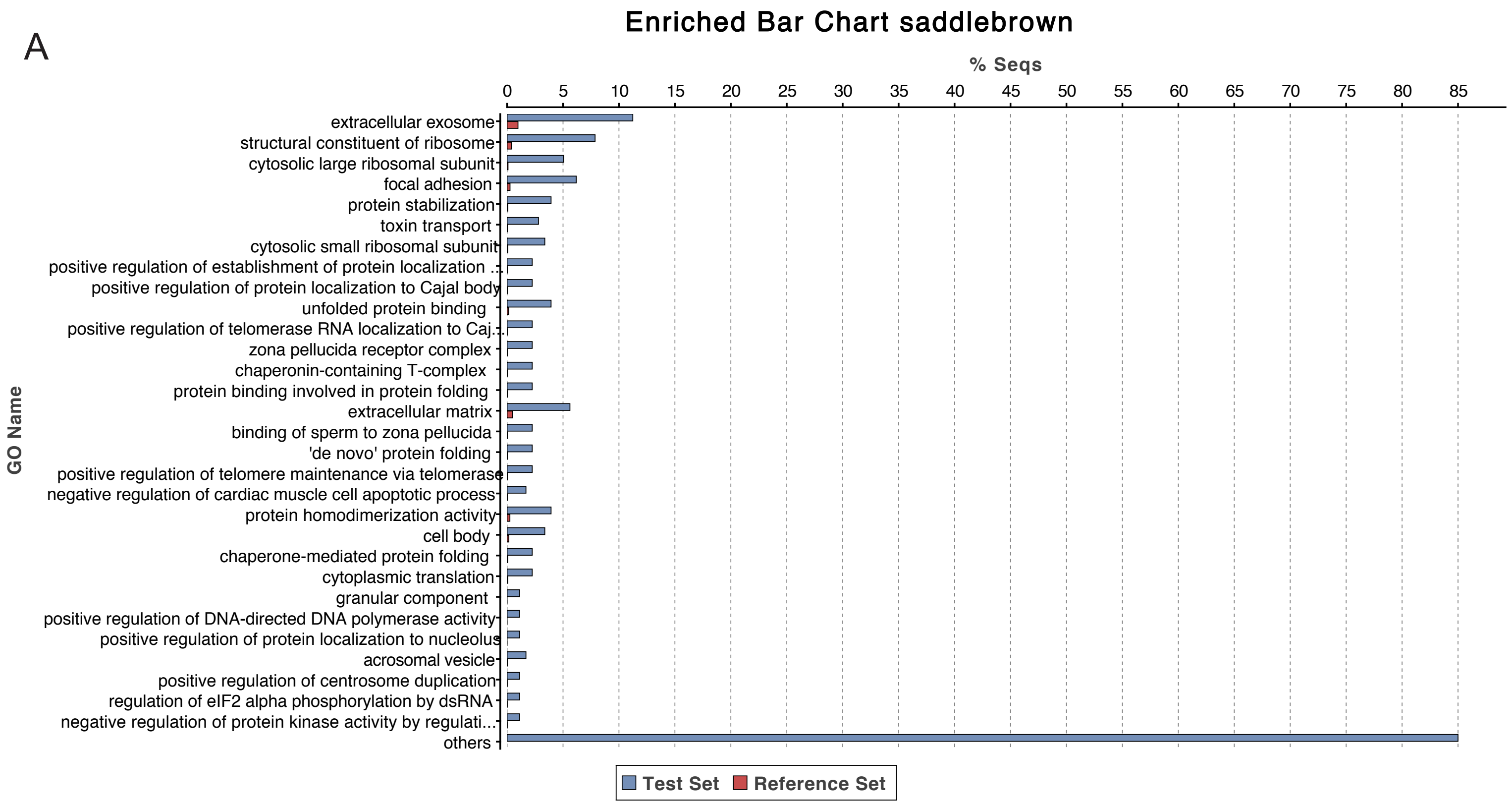

B

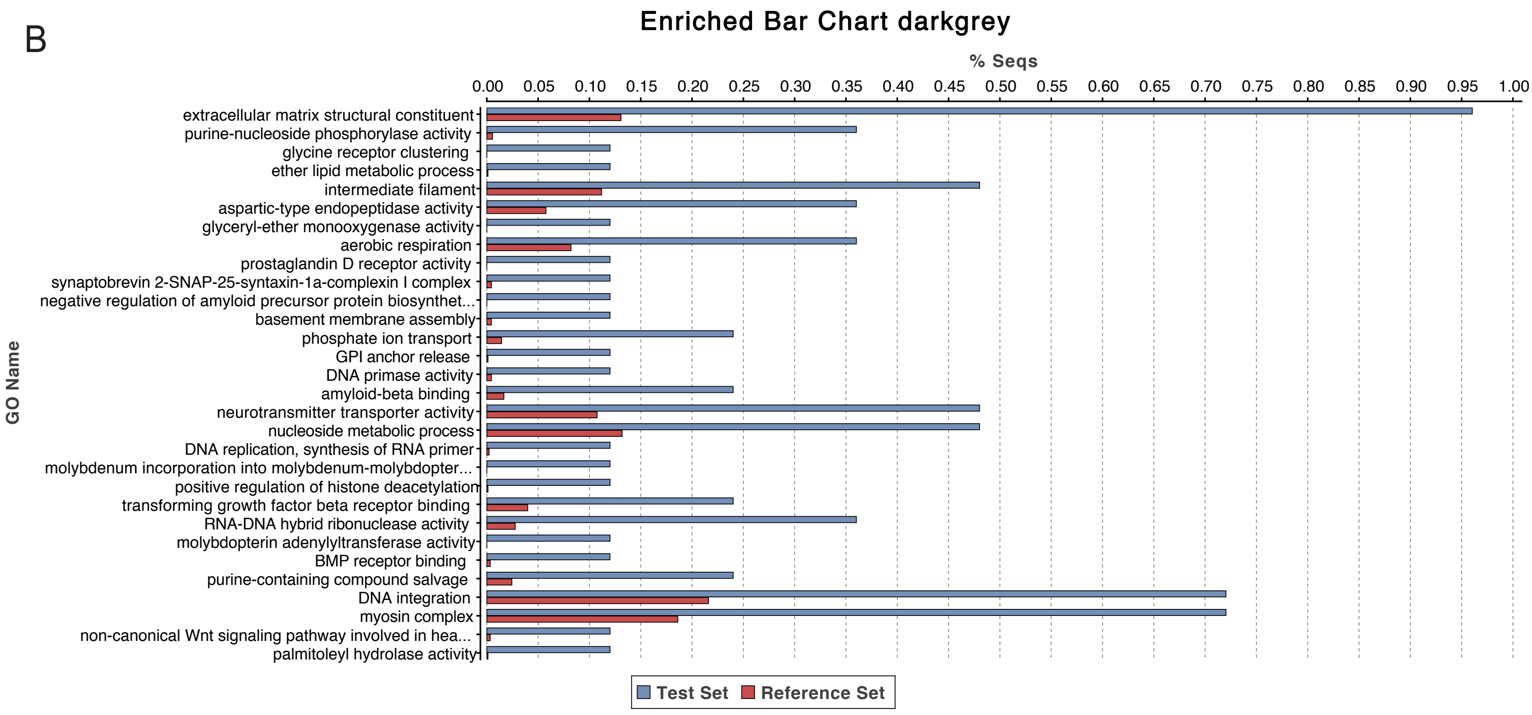

Figure S21

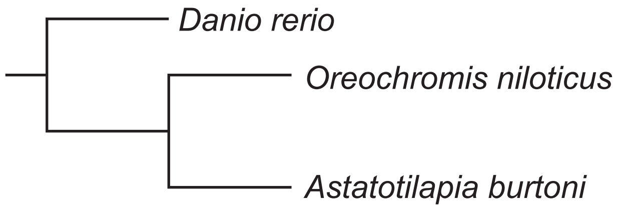
Danio rerio
Oreochromis niloticus
Astatotilapia burtoni

Figure S22

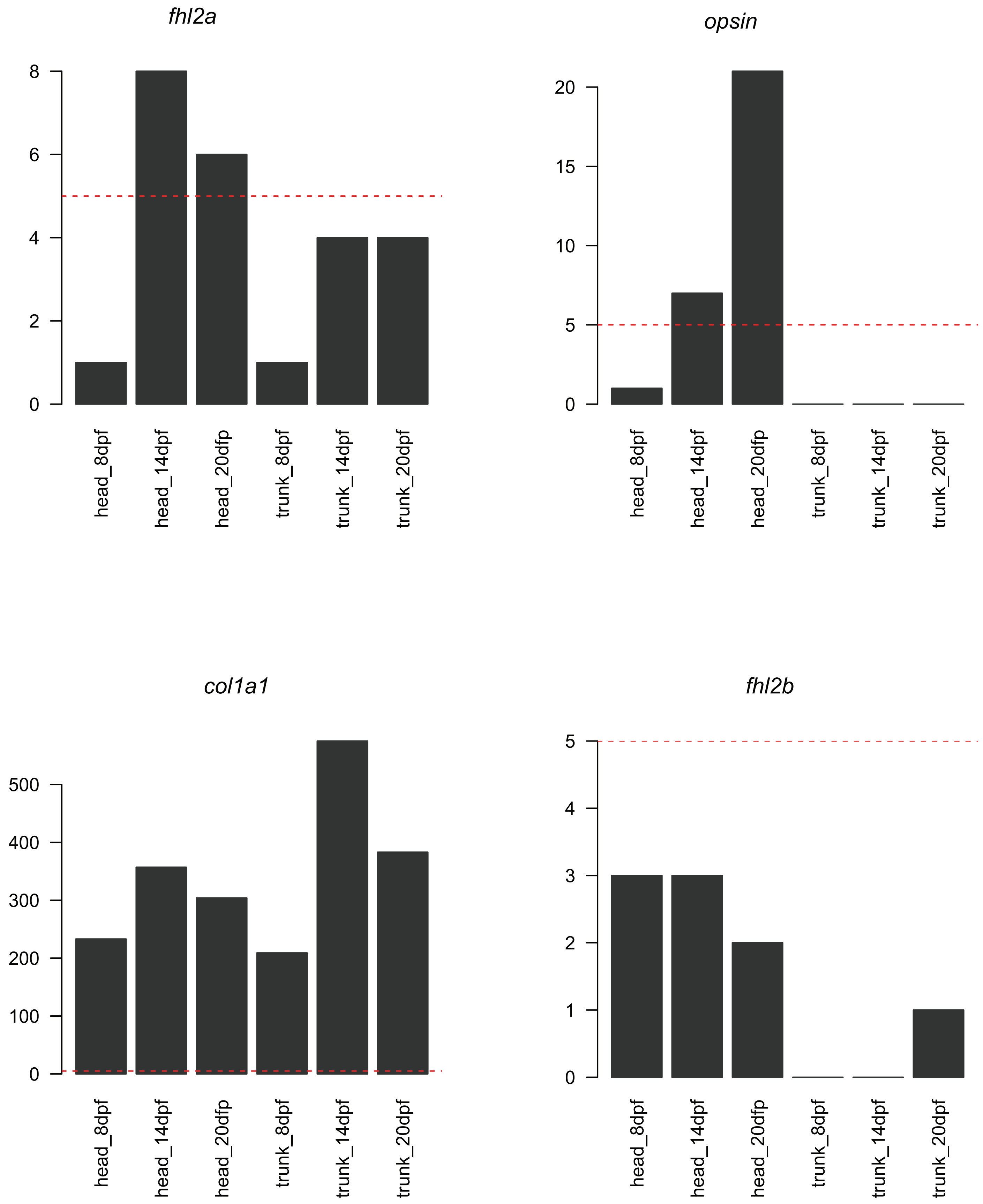

fhl2a
0
2
4
6
8
opsin
0
5
10
15
20
col1a1
0
100
200
300
400
500
fhl2b
0
1
2
3
4
5
head_8dpf
head_14dpf
head_20dfp
trunk_8dpf
trunk_14dpf
trunk_20dpf

Figure S23

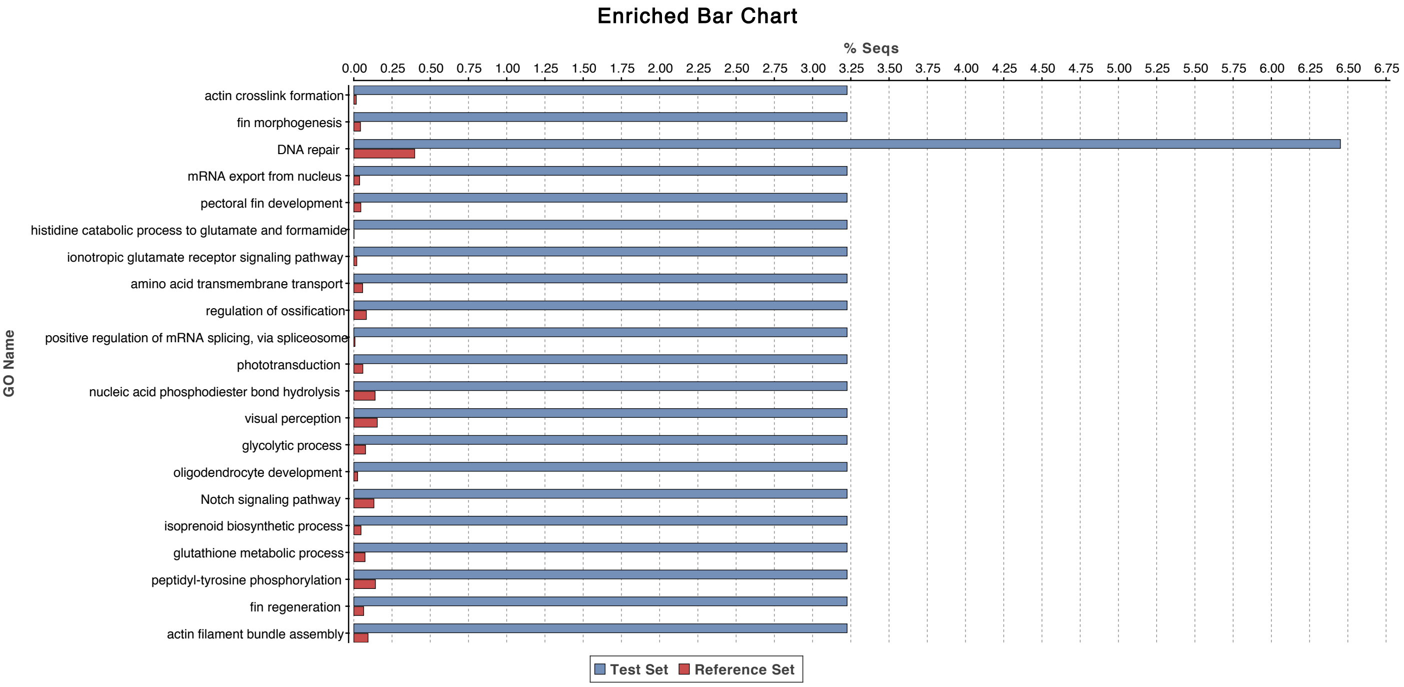
Enriched Bar Chart
% Seqs
0.00
0.25
0.50
0.75
1.00
1.25
1.50
1.75
2.00
2.25
2.50
2.75
3.00
3.25
3.50
3.75
4.00
4.25
4.50
4.75
5.00
5.25
5.50
5.75
6.00
6.25
6.50
6.75
GO Name
actin crosslink formation
fin morphogenesis
DNA repair
mRNA export from nucleus
pectoral fin development
histidine catabolic process to glutamate and formamide
ionotropic glutamate receptor signaling pathway
amino acid transmembrane transport
regulation of ossification
positive regulation of mRNA splicing, via spliceosome
phototransduction
nucleic acid phosphodiester bond hydrolysis
visual perception
glycolytic process
oligodendrocyte development
Notch signaling pathway
isoprenoid biosynthetic process
glutathione metabolic process
peptidyl-tyrosine phosphorylation
fin regeneration
actin filament bundle assembly
Test Set
Reference Set

Figure S24

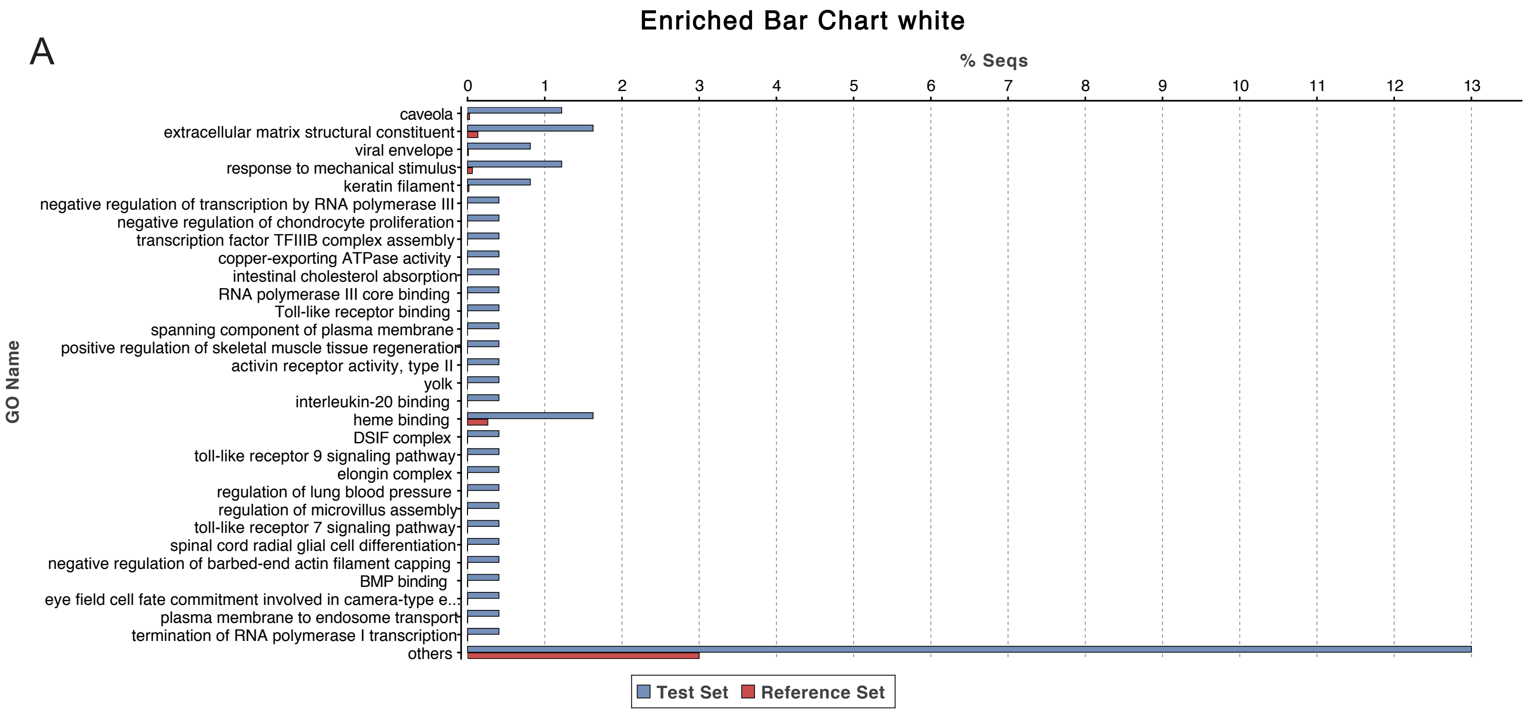
A
Enriched Bar Chart white
% Seqs
0
1
2
3
4
5
6
7
8
9
10
11
12
13
GO Name
caveola
extracellular matrix structural constituent
viral envelope
response to mechanical stimulus
keratin filament
negative regulation of transcription by RNA polymerase III
negative regulation of chondrocyte proliferation
transcription factor TFIIIB complex assembly
copper-exporting ATPase activity
intestinal cholesterol absorption
RNA polymerase III core binding
Toll-like receptor binding
spanning component of plasma membrane
positive regulation of skeletal muscle tissue regeneration
activin receptor activity, type II
yolk
interleukin-20 binding
heme binding
DSIF complex
toll-like receptor 9 signaling pathway
elongin complex
regulation of lung blood pressure
regulation of microvillus assembly
toll-like receptor 7 signaling pathway
spinal cord radial glial cell differentiation
negative regulation of barbed-end actin filament capping
BMP binding
eye field cell fate commitment involved in camera-type e...
plasma membrane to endosome transport
termination of RNA polymerase I transcription
others
Test Set
Reference Set

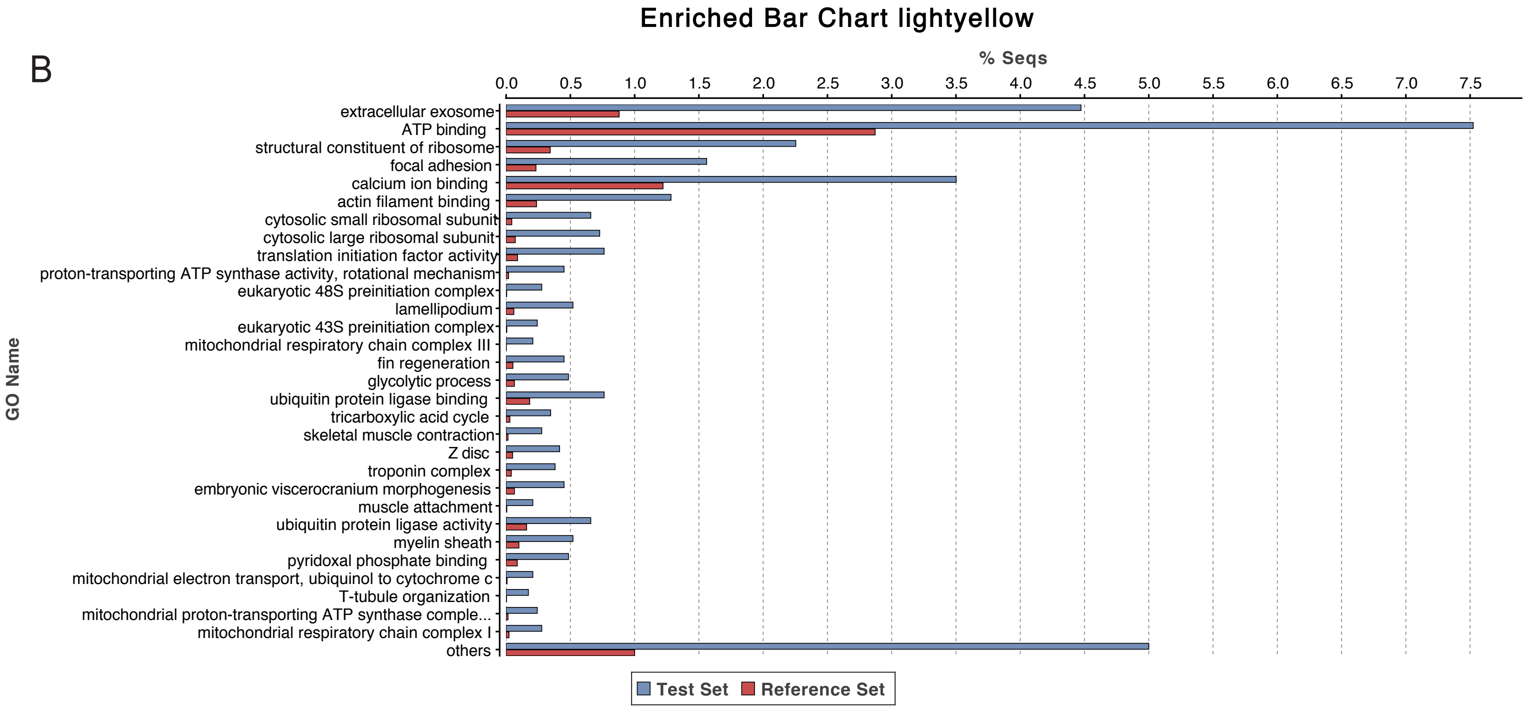
B
Enriched Bar Chart lightyellow
% Seqs
0.0
0.5
1.0
1.5
2.0
2.5
3.0
3.5
4.0
4.5
5.0
5.5
6.0
6.5
7.0
7.5
GO Name
extracellular exosome
ATP binding
structural constituent of ribosome
focal adhesion
calcium ion binding
actin filament binding
cytosolic small ribosomal subunit
cytosolic large ribosomal subunit
translation initiation factor activity
proton-transporting ATP synthase activity, rotational mechanism
eukaryotic 48S preinitiation complex
lamellipodium
eukaryotic 43S preinitiation complex
mitochondrial respiratory chain complex III
fin regeneration
glycolytic process
ubiquitin protein ligase binding
tricarboxylic acid cycle
skeletal muscle contraction
Z disc
troponin complex
embryonic viscerocranium morphogenesis
muscle attachment
ubiquitin protein ligase activity
myelin sheath
pyridoxal phosphate binding
mitochondrial electron transport, ubiquinol to cytochrome c
T-tubule organization
mitochondrial proton-transporting ATP synthase comple...
mitochondrial respiratory chain complex I
others
Test Set
Reference Set

Figure S25

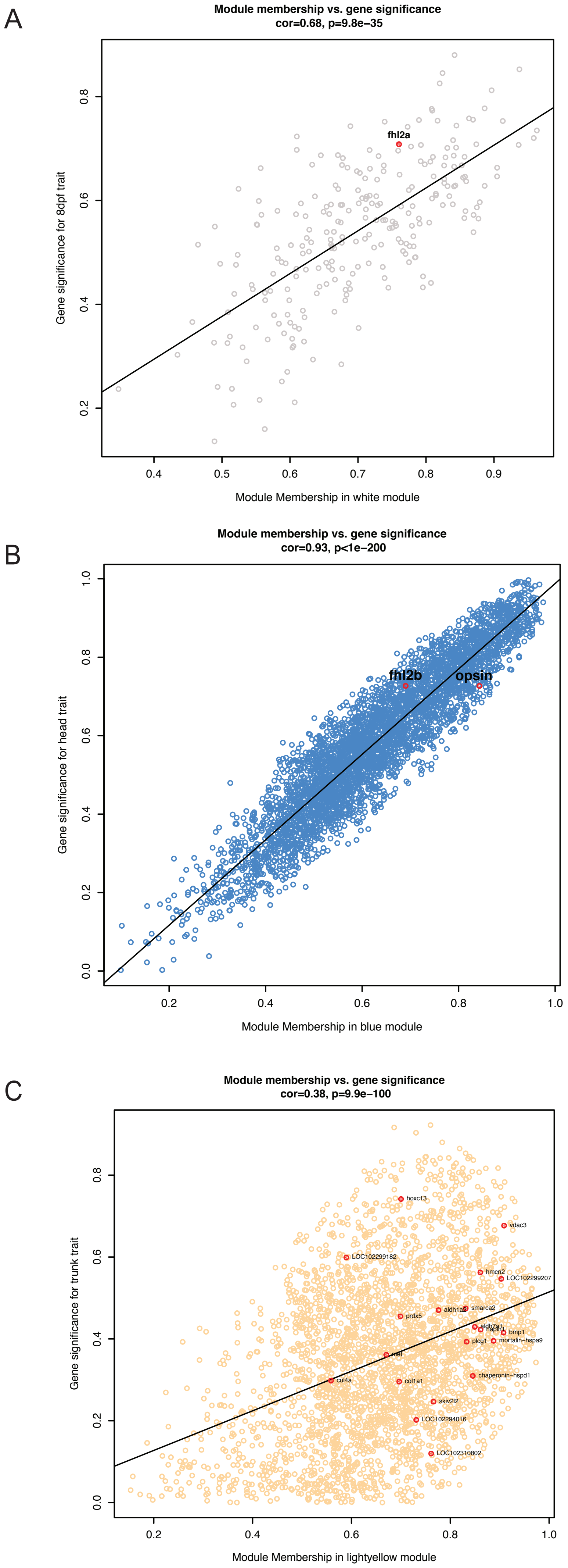
A
Module membership vs. gene significance
cor=0.68, p=9.8e−35
fhl2a
Gene significance for 8dpf trait
Module Membership in white module
B
Module membership vs. gene significance
cor=0.93, p<1e−200
fhl2b
opsin
Gene significance for head trait
Module Membership in blue module
C
Module membership vs. gene significance
cor=0.38, p=9.9e−100
hoxc13
vdac3
LOC102299182
hmcn2
LOC102299207
aldh1a2
smarca2
prdx5
aldh7a1
bmp1
plcg1
mortalin−hspa9
met
chaperonin−hspd1
cul4a
col1a1
skiv2l2
LOC102294016
LOC102310802
Gene significance for trunk trait
Module Membership in lightyellow module

FigureS26

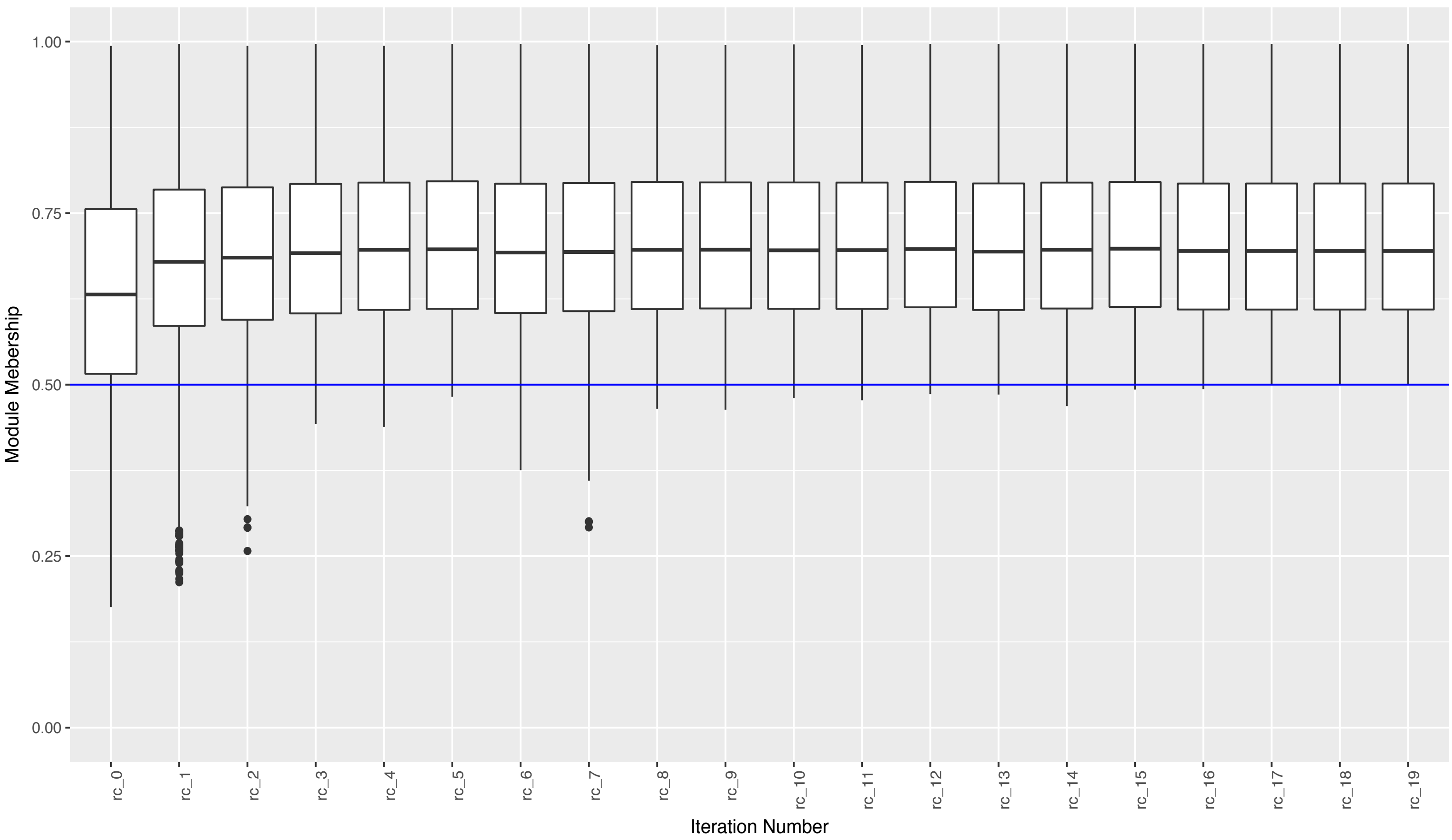

1.00
0.75
0.50
0.25
0.00
Module Mebership
rc_0
rc_1
rc_2
rc_3
rc_4
rc_5
rc_6
rc_7
rc_8
rc_9
rc_10
rc_11
rc_12
rc_13
rc_14
rc_15
rc_16
rc_17
rc_18
rc_19
Iteration Number

Figure S27

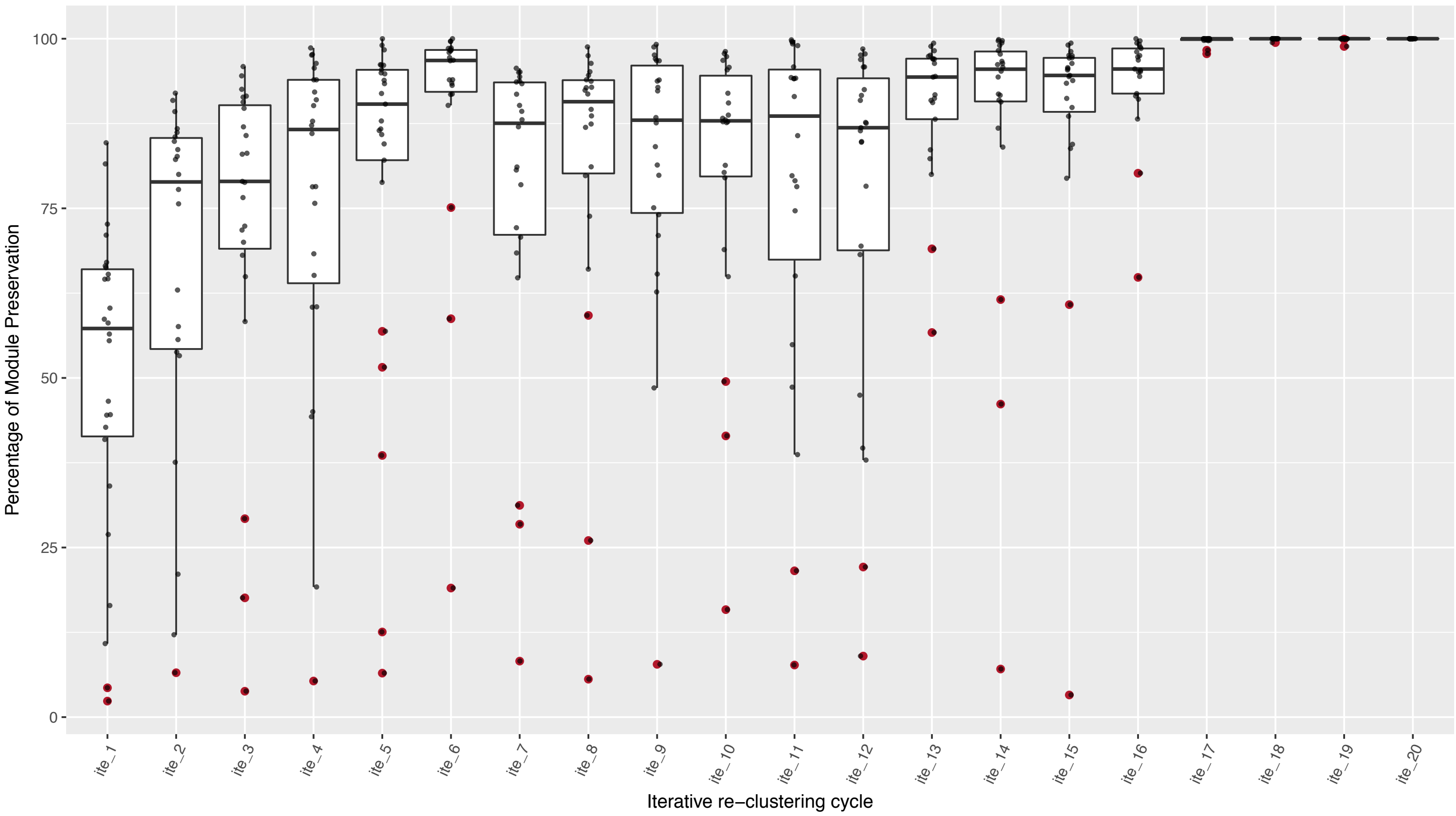
Percentage of Module Preservation
100
75
50
25
0
ite_1
ite_2
ite_3
ite_4
ite_5
ite_6
ite_7
ite_8
ite_9
ite_10
ite_11
ite_12
ite_13
ite_14
ite_15
ite_16
ite_17
ite_18
ite_19
ite_20
Iterative re−clustering cycle

Figure S28
